# Genetic Association Studies in Hepatocellular Carcinoma: Systematic Meta‐Analyses, a Comprehensive Field Synopsis, and Epidemiological Evidence

**DOI:** 10.1002/mco2.70153

**Published:** 2026-08-26

**Authors:** Yujin Shi, Bin Huang, Xiaohong Liu, Yalan Liu, Qinyong Hu, Tianyu Lei, Luqi Tang, Guanglin Xiao, Tingting Ning, Shengqin Yue, Jiahui He, Qilian Du, Xuanmei Li, Yixuan Zhou, Qiongfang Zhang, Chunyan Qi, Hong Ren, Hong‐Gang Ren

**Affiliations:** ^1^ Department of Infectious Diseases Key Laboratory of Molecular Biology for Infectious Diseases (Ministry of Education) Institute for Viral Hepatitis The Second Affiliated Hospital Chongqing Medical University Chongqing China; ^2^ Department of Critical Care Medicine The Second Affiliated Hospital of Chongqing Medical University Chongqing China; ^3^ Department of Oncology Renmin Hospital of Wuhan University Wuhan China; ^4^ Department of Pediatric Union Hospital, Tongji Medical College, Huazhong University of Science and Technology Wuhan China; ^5^ Department of Cardiology The Second Affiliated Hospital of Chongqing Medical University Chongqing China

**Keywords:** genetic, hepatocellular carcinoma, meta‐analysis, polymorphism, variant

## Abstract

Associations between various genetic variants and the risk of hepatocellular carcinoma (HCC) have been extensively explored but produced contradictory results. The aim of the present systematic meta‐analysis was to determine and validate genetic variants that are associated with HCC risk. Two‐step literature searches of PubMed, Embase, Web of Science, and Google Scholar databases and various meta‐analyses were performed, and a comprehensive field synopsis and epidemiological evidence were provided. A total of 20,081 publications were identified, of which 830 were deemed eligible for inclusion. Eventually, 36 variants in 27 genes were identified to be associated with HCC risk. Moreover, cumulative epidemiological evidence of an association was graded as moderate for nine variants in eight genes (*ESR1* rs2234693, *GRP78* rs430397, *HLA‐DP* rs3077, *HLA‐DQ* rs2856718, *MnSOD* rs4880, *TNFα* rs361525, *HFE* rs1800562 and rs1799945, and *UGT1A7* High/Low) and strong for three variants in three genes (*IL‐1B* rs1143627, *COL18A1* rs7499, and *NQO1* rs1800566); *HFE* rs1800562 was deemed to have a false‐positive association. Thus, 11 variants in 11 genes were identified to be associated with HCC risk. This synopsis helps elucidate the mechanisms of carcinogenesis of HCC and provides insights into the early diagnosis and novel treatments of HCC by targeting those potential genes.

## Graphical abstract

Based on meta‐analyses and cumulative epidemiological evidence, the present study identifies 11 genetic variants in 11 genes that are truly associated with the risk of hepatocellular carcinoma. Our work presents a systematic synopsis, which helps elucidate the mechanisms of carcinogenesis and provides insights into the early diagnosis and novel treatments of HCC by targeting these potential genes.

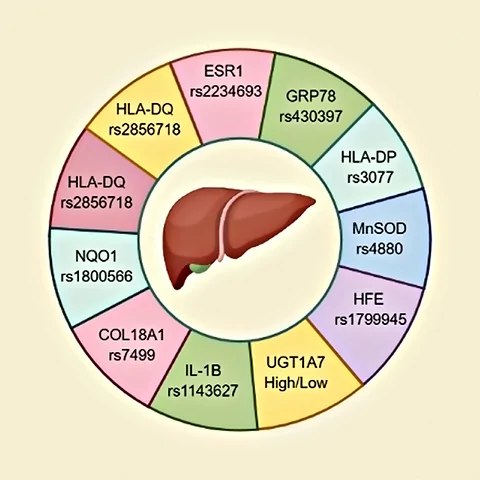

## 1 Introduction

Liver cancer is the fourth most common cause of death globally, with an estimated 866,136 new cases and 758,725 new deaths in 2023 [1]. Hepatocellular carcinoma (HCC), a primary type of liver cancer, accounting for 90% of liver cancer cases, is usually diagnosed at an advanced stage owing to its insidious onset at an early stage, leaving behind limited treatment options [2, 3]. Due to its high incidence, high drug resistance, high metastasis, and refractory nature, HCC has been receiving widespread attention from basic and clinical researchers [1, 2].

Previous studies have demonstrated that HCC is highly heterogeneous and involves the accumulation of somatic mutations and epigenetic modifications which are caused by sustained inflammatory damage due to various chronic liver diseases [1, 3]. Recently developed high‐throughput sequencing technologies have enabled the construction of a comprehensive genetic map of HCC by analyzing large genetic datasets [7]. For example, mutations in the *TERT* promoter region and *TP53* and *CTNNB1* genes have been found to be multiple foci of alterations in HCC [4, 5, 6]. Genome‐wide association studies (GWAS) were initiated in 2002, and since have led to the discovery of some common genetic risk variants for HCC (Tables 1, 2) [7]. In addition, with the evolution of molecular biology and molecular genetics, studies on polymorphisms of genes related to the pathogenesis of HCC are blooming [8, 9].

**TABLE 1:** Genes or chromosomal loci with a strong or moderate association with risk of hepatocellular carcinoma as identified in previous genome‐wide association studies.

Gene/Chr loci	Variant	OR	95%CI	*p* value	Population frequency
*1p36.33*	CNV	17.01	5.16–88.42	1.11 × 10^−10^	<1%
*15q13.3*	Duplication	12.02	NA	3.17 × 10^−8^	<1%
*DLC1*	rs77236434	9.87	3.46–28.16	2.64 × 10^−7^	1%
*CDH13*	rs16960234	7.22	1.30–40.0	NA	NA
*PNPLA3*	rs2896019	3.37	2.21–5.14	1.8 × 10^−8^	46%
*DYSF*	rs17007417	2.74	1.84–4.06	5.2 × 10^−7^	17%
*HLA‐DQB1*	rs2856723	2.68	2.32–3.09	2.58 × 10^−43^	26%
*HLA‐DQB1*	rs2858324	2.42	2.11–2.78	1.09 × 10^−36^	25%
*TLL1*	rs17047200	2.38	1.56–3.64	3.74 × 10^−5^	48%
*GLB1*	rs4678680	2.27	1.68–3.08	2 × 10^−7^	7%
*DEPDC5*	rs1012068	2.20	1.64–2.97	8.0 × 10^−8^	10%
*HLA‐DQB1*	rs9275086	2.10	1.84–2.40	7.38 × 10^−28^	26%

Abbreviations: Chr = chromosomal, CI = confidence interval, CNV = copy number variant, NA = not available, OR = odds ratio.

**TABLE 2:** Genes or chromosomal loci with a low association with risk of hepatocellular carcinoma as identified in previous genome‐wide association studies.

Gene/Chr loci	Variant	OR	95%CI	*p* value	Population frequency
*C2*	rs9267673	1.97	1.47–2.64	2 × 10^−6^	8%
*HLA‐DRB1*	rs2647073	1.94	1.40–2.69	6 × 10^−5^	6%
*GCKR*	rs1260326	1.84	1.23–2.75	0.003	57%
*PNPLA3*	rs738409	1.67	1.16–2.40	0.005	35%
*GATAD2A*	rs4808199	1.59	1.08–2.35	0.020	27%
*C14orf143*	rs12100561	1.52	1.26–1.83	4 × 10^−6^	39%
*TM6SF2*	rs58542926	1.45	1.08–1.94	0.010	8%
*8p12*	rs12682266	1.38	NA	3.76 × 10^−5^	48%
*HLA‐DQB1*	rs9275210	1.36	1.20–1.54	1.63 × 10^−6^	31%
*MICA*	rs2596542	1.34	1.16–1.53	4.5 × 10^−5^	33%
*CDK14*	rs10272859	1.28	1.18–1.38	9.46 × 10^−10^	32%
*HLA‐DQA1/DRB1*	rs9272105	1.28	1.22–1.35	5.24 × 10^−22^	45%
*HLA‐DQB1‐AS1*	rs2647046	1.26	1.11–1.43	4.14 × 10^−4^	15%
*STAT4*	rs7574865	1.22	1.15–1.29	1.66 × 10^−11^	67%
*LINC01149*	rs2844512	1.21	1.11–1.32	1.043 × 10^−5^	36%
*SAMM50*	rs3761472	1.19	1.00–1.42	0.040	24%
*GRIK1*	rs455804	0.84	0.80–0.89	5.24 × 10^−10^	34%
*HSD17B13*	rs72613567	0.78	0.69–0.89	1.22 × 10^−4^	22%
*KIF1B*	rs17401966	0.53	0.41–0.70	5.8 × 10^−6^	73%

Abbreviations: Chr = chromosomal, CI = confidence interval, NA = not available, OR = odds ratio.

Determination of candidate genetic association is now the most common approach for screening malignancy susceptible alleles and genes, and indeed, genetic association studies have been able to identify genetic polymorphisms of genes such as *IL‐8*, *XRCC3*, and *MDM2* that contribute to HCC vulnerability [10, 11, 12].

Over the past two decades, thousands of genetic association studies have been carried out, but discrepant or even contradictory results have been obtained, mainly due to small sample sizes and varied statistical significance thresholds used for subjectivity when searching for candidate genes [13].

Therefore, the aim of the present systematic meta‐analysis was to determine and validate genetic variants that are associated with HCC risk in order to provide an updated understanding of the genetic predisposition for HCC.

## 2 Results

### 2.1 Characteristics of Eligible Studies and Studied Genetic Variants

**FIGURE 1:**
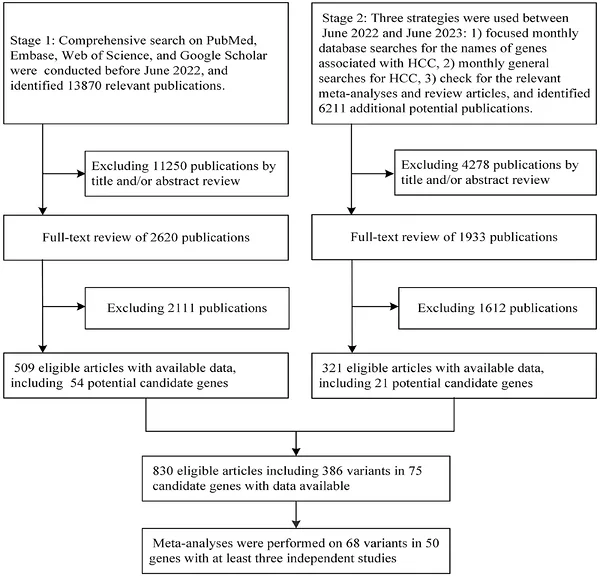
Flow diagram of literature search and selection.

We identified 830 eligible publications (including original articles, review articles, and meta‐analyses), involving 75 genes and 386 variants (Figure 1). Sixty‐eight genetic variants in 50 candidate genes present in at least three original studies were included in our study for further meta‐analyses (Table S1). A total of 67 main meta‐analyses (allelic contrast) of 539 original articles with a mean pooled sample size of 4220 (range 598–12,749) was performed. Among the 68 variants, most were single nucleotide polymorphisms (SNPs; *N* = 61) located in noncoding transcript regions (*N* = 19, including DNA upstream or downstream regions), 5′ or 3′ untranslated regions (UTRs; *N* = 6), intron regions (*N* = 14), and intergenic regions (*N* = 1); the remaining variants were missense variants (*N* = 18), nonsense mutations (*N* = 1), and synonymous variants (*N* = 2) in exon regions. In addition, six phenotype traits and one 14 bp insertion/deletion in 3′ UTRs were found. All variants had minor allele frequencies or rare phenotypes greater than 5%.

### 2.2 Variants Associated with HCC Risk as Identified in the Allelic Model

The meta‐analyses showed significant associations between 27 variants in 22 genes and HCC risk (Table 3). Strong associations with HCC risk were observed in six genetic variants; two missense variants in *XRCC1* (G > C, rs139599857) and *MnSOD* (T > C, rs4880), one intron variant in *ESR1* (T > C, rs2234693), and one non‐coding transcript variant in *TNFα* (G > A, rs1800629) were positively associated with an increased risk of HCC (all summary odds ratio [ORs] > 1.5), whereas one intergenic variant in *HLA‐DQ* (A > G, rs2856718) and one intronic variant in IL‐6 (G > C, rs1800795) showed significant protective effects against HCC (both ORs ≤ 0.67). Moderate associations with HCC risk were detected in 19 variants; 13 variants (*GRP78* rs430397, *GSTP1* rs1695, *HFE* rs1800562, *IL‐28B* rs12979860, *MDM2* rs2279744, *miR‐34b/C* rs4938723, *NQO1* rs1800566, *TGFβ1* rs1800469, *TNFα* rs1800630, *TNFα* rs361525, *TLR2* rs3804099, *XPD* rs1799793, and *XPD* rs13181) were positively associated with an increased risk of HCC (1.5 > ORs > 1.15). Six variants (*COL18A1* rs7499, *CCND1* rs9344, *HLA‐DP* rs3077, *HLA‐DP* rs9277535, *IL1B* rs1143627, and *IL8* rs2227306) showed a protective effect on HCC risk (0.67 < ORs < 0.87). Weak associations were seen for two variants, namely *HULC* rs7763881 (OR = 0.90) and *XRCC1* rs1799782 (OR = 1.14). Of the 27 variants, 21 variants were studied in all ethnic groups, five in the Asian population only and one in the Caucasian population only. Moreover, a total of 25 variants that showed non‐significant associations with HCC risk in the allelic model were further analyzed in 38 stratified meta‐analyses in terms of ethnicity; 14 were performed on the Caucasian population, including a mean of five (range 3–12) studies and 1275 (range 428–3234) participants, and 24 on the Asian populations, including a mean of seven (range 3–18) studies and 4420 (range 719–11,553) participants (Table S2). Eventually, three additional variants associated with HCC risk were identified; the phenotype variants of *GSTT1* (present/null) in Caucasians only and *UGT1A7* (high/low) in Asians only showed strong associations with HCC risk, whereas one missense variant in *XRCC1* (rs25487) had a moderate association in Asians only (Table 3). The associations of the remaining 22 variants did not differ between Asians and Caucasians.

**TABLE 3:** Genetic variants significantly associated with risk of hepatocellular carcinoma as confirmed in meta‐analyses in the allelic model.

								Liver‐cancer risk		Heterogeneity				
*Genes*	Variants	Allele^a^	Ethnicity	Studies	Cases	Controls	MAF (%)	OR (95% CI)	*p* value	*p* value	*I* ^2^	Venice criterie grade^b^	Cumulative evidence of association^c^	FPRP^d^
*COL18A1*	rs7499	C vs. T	Asian	3	808	1067	47.23	0.73(0.64–0.83)	2.32 × 10^−6^	0.784	0%	AAA	+++	<0.001
*CCND1*	rs9344	G vs. A	All	5	805	1255	48.13	0.69(0.50–0.94)	0.020	0.001	80%	ACA	+	0.223
*ESR1*	rs2234693	C vs. T	All	3	367	458	43.16	1.51(1.22–1.86)	1.40 × 10^−4^	0.145	48%	BBA	++	0.003
*GRP78*	rs430397	A vs. G	All	3	1011	1046	15.70	1.42(1.20–1.69)	5.66 × 10^−5^	0.552	0%	BAA	++	0.001
*GSTP1*	rs1695	G vs. A	All	7	1144	1444	28.36	1.45(1.08–1.95)	0.014	0.002	74%	ACA	+	0.176
*HFE*	rs1800562	A vs. G	All	14	1221	3334	5.13	1.28(1.02–1.61)	0.033	0.020	49%	BBA	++	0.241
*HLA‐DP*	rs3077	A vs. G	All	4	1865	3113	35.51	0.77(0.70–0.84)	3.38 × 10^−8^	0.172	40%	ABA	++	<0.001
*HLA‐DP*	rs9277535	A vs. G	All	6	2238	4067	41.25	0.79(0.64–0.99)	0.036	0.000	81%	ACA	+	0.257
*HLA‐DQ*	rs2856718	G vs. A	All	4	1974	2831	43.24	0.67(0.61–0.73)	1.67 × 10^−20^	0.126	48%	ABA	++	NA
*HULC*	rs7763881	C vs. A	All	3	1887	2222	47.56	0.90(0.82–0.98)	0.015	0.278	22%	AAC	+	0.118
*IL1B*	rs1143627	C vs. T	Asian	7	1331	1409	46.11	0.83(0.74–0.92)	0.001	0.230	26%	AAA	+++	0.007
*IL‐6*	rs1800795	C vs. G	All	5	346	911	49.16	0.63(0.42–0.95)	0.025	0.109	55%	ACC	+	0.373
*IL8*	rs2227306	T vs. C	Asian	3	486	698	36.40	0.78(0.66–0.94)	0.006	0.176	42%	BBC	+	0.055
*IL‐28B*	rs12979860	T vs. C	All	17	4267	4862	33.85	1.30(1.08–1.58)	0.007	0.000	70%	ACC	+	0.075
*MDM2*	rs2279744	G vs. T	All	12	3011	3219	47.88	1.39(1.22–1.60)	1.33 × 10^−6^	0.001	64%	ACA	+	<0.001
*miR‐34b/C*	rs4938723	C vs. T	Asian	6	3134	3624	33.00	1.23(1.06–1.44)	0.008	0.001	75%	ACC	+	0.067
*MnSOD*	rs4880	C vs. T	Caucasian	3	238	360	48.58	1.82(1.43–2.32)	1.45 × 10^−6^	0.474	0%	BAA	++	<0.001
*NQO1*	rs1800566	T vs. C	All	3	740	765	40.70	1.25(1.08–1.45)	0.003	0.676	0%	AAA	+++	0.028
*TGFβ1*	rs1800469	T vs. C	All	9	2160	3255	46.42	1.24(1.02–1.50)	0.030	0.000	79%	ACC	+	0.218
*TNFα*	rs1800629	A vs. G	All	18	2125	3663	11.63	1.58(1.18–2.12)	0.002	0.000	75%	ACA	+	0.050
*TNFα*	rs1800630	A vs. C	All	10	1994	2957	18.78	1.34(1.12–1.61)	0.002	0.022	55%	ACA	+	0.017
*TNFα*	rs361525	A vs. G	All	8	763	1175	12.02	1.49(1.16–1.91)	0.002	0.128	38%	BBA	++	0.030
*TLR2*	rs3804099	C vs. T	All	4	1213	2599	40.29	1.33(1.05–1.69)	0.019	0.004	78%	ACC	+	0.172
*XPD*	rs1799793	T vs. C	Asian	4	1500	1650	22.93	1.20(1.06–1.36)	0.006	0.710	0%	AAC	+	0.045
*XPD*	rs13181	A vs. T	All	8	3457	3940	27.61	1.36(1.01–1.83)	0.042	0.000	91%	ACC	+	0.336
*XRCC1*	rs139599857	C vs. G	All	3	761	733	35.04	2.61(1.20–5.69)	0.016	0.000	89%	ACA	+	0.633
*XRCC1*	rs1799782	G vs. C	All	8	1259	1385	24.24	1.14(1.00–1.31)	0.047	0.101	43%	ABC	+	0.002
Additional variants after stratification by ethnicity														
*GSTT1*	Present/Null	Null vs. present	Caucasian	7	1030	2059	24.12	1.56(1.07‐2.28)	0.022	0.000	77%	BCA	+	0.317
*UGT1A7*	High/Low	Low vs. High	Asian	4	294	425	32.62	2.11(1.54‐2.90)	4.16 × 10^−6^	0.283	21%	BAA	++	0.002
*XRCC1*	rs25487	G vs. A	Asian	11	4029	4227	26.91	1.44(1.19‐1.75)	1.70 × 10^−4^	0.000	83%	ACC	+	0.002

Abbreviations: A = adenine, C = cytosine, CI = confidence interval, FPRP = false positive report probability, G = guanine, MAF = minor allele frequency, OR = odds ratio, T = thymine.

^a^ Minor versus major allele (reference).

^b^ Venice criteria grades are for amount of evidence, replication of the association, and protection from bias.

^c^ Cumulative epidemiological evidence was graded as strong (+++), moderate (++), or weak (+) for association with HCC risk by Venice criteria.

^d^ FPRP was calculated with a prior probability of 0.1 and an OR of 1.5 (risk effects) or 0.67 (protective effects).

XPD rs1799793 and rs13181 were observed in moderate linkage disequilibrium (LD) (*R* ^2^ = 0.482).

### 2.3 Variants Associated With HCC Risk as Identified in the Recessive and Dominant Models

Seven additional variants were found to be associated with significantly increased or decreased risk for HCC in other genetic models, including two in the recessive models and five in the dominant models (Table 4 and Figure S1) although they showed nonsignificant associations in the allelic model. Of the seven variants, only an intronic variant in IL‐1B (rs16944) showed a protective effect against HCC, whereas the other six variants were associated with an increased risk of HCC. Seven meta‐analyses revealed that the strongest association with HCC was the CC genotype of EPHX rs1051740 (ORs = 1.48, 95% CI: 1.09–2.01, *p* = 0.012). In addition, XRCC1 rs25487 not only had a moderate association with HCC risk in Asians in the allelic model but was also moderately significantly associated with HCC risk in both Asians and Caucasians in the recessive model (GG vs. GA+AA).

**TABLE 4:** Additional genetic variants significantly associated with risk of hepatocellular carcinoma as confirmed in meta‐analyses in the recessive or dominant model.

							Allelic contrasts			Best genetic model						
Genes	Variants	Alleles^a^	Studies	Cases	Controls	MAF (%)	OR (95%CI)	*p* value	*I* ^2^	Model	OR (95%CI)	*p* value	*I* ^2^	Venice criteria grade^b^	Cumulative evidence of association^c^	FPRP^d^
*EGF61*	rs4444903	A vs. G	16	1943	3505	46.52	1.26(0.99–1.60)	0.052	83.50%	DOM	1.32(1.02–1.70)	0.038	66%	ACC	+	0.275
*EPHX*	rs1051740	C vs. T	11	816	1780	31.21	1.21(0.87–1.67)	0.253	71.00%	REC	1.48(1.09–2.01)	0.012	24%	BAC	+	0.162
*HFE*	rs1799945	G vs. C	16	1282	2468	15.21	1.15(0.91–1.45)	0.247	53.40%	DOM	1.22(1.05–1.43)	0.012	44 %	ABB	++	0.098
*IL‐1B*	rs16944	T vs. C	5	949	974	47.82	0.90(0.66–1.21)	0.468	73.30%	DOM	0.78(0.63–0.97)	0.024	42%	ABC	+	0.190
*IL‐28B*	rs8099917	G v vs. T	13	3864	4156	17.99	1.08(0.95–1.23)	0.245	36.10%	DOM	1.33(1.06–1.68)	0.014	59%	ACB	+	0.131
*miR‐196a2*	rs11614913	C vs. T	17	4992	5910	48.34	1.08(0.97–1.21)	0.147	70.60%	DOM	1.18(1.00–1.39)	0.047	63%	ACC	+	0.295
*XRCC1*	rs25487	G vs. A	25	5189	6362	28.85	1.18(0.99–1.41)	0.062	83.90%	REC	1.31(1.01–1.71)	0.044	680%	ACC	+	0.322

Abbreviations: A = adenine, C = cytosine, DOM = dominant, FPRP = false positive report probability, G = guanine, MAF = minor allele frequency, REC = recessive, T = thymine.

^a^ Minor allele versus major allele (reference).

^b^ Venice criteria grade was based on amount of evidence, replication of the association, and protection from bias.

^c^ Cumulative epidemiological evidence was graded as strong (+++), moderate (++), or weak (+) for association with HCC risk by Venice criteria.

^d^ FPRP was calculated with a prior probability of 0.1 and an OR of 1.5 (risk effects) or 0.67 (protective effects).

### 2.4 Variants Associated with the Risk of HCC Related to Viral Hepatitis

A stratified meta‐analysis by underlying was performed to analyze variants associated with the risk of HCC in terms of chronic viral hepatitis, mainly chronic hepatitis B (HBV) and C (HCV) infection; the number of studies on other liver diseases was insufficient for meta‐analysis. Nine genetic variants were found to be significantly associated with the risk of viral hepatitis‐related HCC; four variants (*HLA‐DP* rs3077, *HLA‐DP* rs92775353, *IL‐1B* rs16944, and*IL‐1B* rs1143627) were protective, and five variants (*MDM2* rs2279744, *HFE* rs1799945, *TNFα* rs1800630, *IL‐28B* rs12979860, and *IL‐28B* rs8099917) were harmful (Table S3). Their effects in viral hepatitis‐related HCC were consistent with those in the overall HCC.

### 2.5 Variants Without Significant Associations With HCC Risk

Meta‐analyses confirmed that 32 variants in 29 genes were not associated with the risk of HCC in any models. These meta‐analyses included a median of seven studies (range 3–24) and 4067 participants (range 825–12,749). Meta‐analyses with a minimum number of 800 cases and 800 controls confirmed the null association for 25 of the 32 variants, providing reliable evidence (Table 5).

**TABLE 5:** Genetic variants with null association with risk of hepatocellular carcinoma in a meta‐analysis with a minimum number of 800 cases and 800 controls.

Genes	Variants	Comparison^a^	Studies	Cases	Controls	Frequency^b^ (%)	Liver‐cancer risk		Heterogeneity	
							OR (95%CI)	*p* value	*p* value	*I* ^2^
*ALDH2*	rs671	A vs. G	10	2153	5051	27.45	1.06(0.90–1.24)	0.494	0.009	61%
*COX‐2*	rs20417	C vs. G	4	847	857	12.44	0.99(0.55–1.79)	0.981	0.000	87%
*COX‐2*	rs689466	A vs. G	5	1537	1547	46.14	1.15(0.95–1.39)	0.166	0.030	63%
*CTLA4*	rs231775	A vs. G	5	1971	2464	32.98	0.99(0.76–1.30)	0.982	0.000	86%
*CYP2E1*	rs2031920	T vs. C	11	1297	2355	14.11	0.99(0.71–1.39)	0.952	0.012	59%
*GSTM1*	Present/Null	Null vs. Present	24	4209	6971	49.80	1.13(0.92–1.39)	0.238	0.000	83%
*HLA‐DP*	rs7453920	GG vs. GA+AA	3	1409	1946	23.07	0.96(0.62–1.51)	0.873	0.116	54%
*HLA‐G*	rs371194629	Ins vs. del	5	1051	1397	28.70	1.02(0.76–1.37)	0.913	0.001	79%
*HOGG1*	rs1052133	G vs. C	6	919	1553	46.9	1.15(0.87–1.52)	0.336	0.000	81%
*IL‐6*	rs1800796	G vs. C	7	1664	1913	47.13	1.02(0.82–1.26)	0.893	0.038	55%
*IL8*	rs4073	A vs. T	5	1052	1249	41.74	1.07(0.86–1.32)	0.545	0.025	64%
*IL10*	rs1800872	C vs. A	9	2609	3405	44.91	1.04(0.84–1.28)	0.201	0.000	82%
*IL18*	rs1946518	C vs. A	5	805	1255	48.13	0.69(0.50–0.94)	0.020	0.001	80%
*leptin receptor*	rs1137101	A vs. G	3	1141	1617	24.08	1.16(0.76–1.76)	0.488	0.001	86%
*MALAT1*	rs619586	G vs. A	5	2872	4023	8.24	0.95(0.84–1.08)	0.449	0.256	24%
*MBOAT7*	rs641738	T vs. C	3	1413	1903	43.47	1.27(0.96–1.68)	0.098	0.018	75%
*miR‐146a*	rs2910164	C vs. G	20	5546	6638	43.14	1.01(0.91–1.11)	0.919	0.000	66%
*miR‐499*	rs3746444	G vs. A	15	2816	3771	23.90	1.14(0.94–1.37)	0.189	0.000	79%
*miR‐149*	rs2292832	C vs. T	7	1429	2068	45.07	0.95(0.86–1.06)	0.357	0.089	45%
*MTHFR*	rs1801131	C vs. A	8	1447	2664	20.59	0.96(0.75–1.29)	0.732	0.000	77 %
*MTHFR*	rs1801133	T vs. C	17	4973	6693	41.88	1.01(0.89–1.15)	0.887	0.000	78%
*NAT2*	Rapid/Slow	Slow vs. Rapid	7	1055	1302	43.28	0.99(0.81–1.20)	0.890	0.259	22%
*TNFα*	rs1799724	T vs. C	7	1334	2115	16.73	0.98(0.85–1.13)	0.783	0.874	0%
*XRCC1*	rs25489	A vs. G	7	913	1063	16.69	1.19(0.85–1.68)	0.308	0.007	69%
*XRCC3*	rs861539	T vs. C	5	2624	3532	24.22	1.34(0.83–2.14)	0.229	0.000	95%

Abbreviations: A = adenine, C = cytosine, CI = confidence interval, Del = deletion, G = guanine, Ins = insertion, OR = odds ratio, T = thymine.

^a^ Allelic contrast or phenotype for common variants, or genetic comparison with only genotype group data.

^b^ Frequency of minor allele or effect genotype.

### 2.6 Heterogeneity, Sensitivity Analysis, and Publication Bias

Heterogeneity was common among the 67 main meta‐analyses; 43 (64.7%), 11 (16.2%), and 13 (17.6%) showed high (*I* ^2^ > 50%), moderate (25% ≤ *I* ^2^ ≤ 50%), and little or no (*I* ^2^ < 25%) heterogeneity, respectively. The proportion of highly heterogeneous studies accounted for almost half of the meta‐analyses identifying 27 variants in the allelic model (Table 3, *N* = 13, 48.1%). Of the 38 ethnicity‐specific meta‐analyses for 25 variants (Table S2), 28 (73.7%) meta‐analyses for 19 genetic variants had high heterogeneity (*I* ^2^ > 50%). The heterogeneity of meta‐analyses was reduced only in four variants after stratification by ethnicity, three (*EGF61* rs4444903, *IL18* rs1946518, and *UGT1A7* High/Low) in Asians and one (*miR‐146a* rs2910164) in Caucasians. Of the 302 meta‐analyses using the other five models (Table S1), 108 (35.8%) showed no or minor heterogeneity, 47 (15.6%) showed moderate heterogeneity, and 147 (48.7%) showed high heterogeneity.

Sensitivity analyses were conducted for all variants significantly associated with HCC risk, first by excluding the first published or first positive report, and then by excluding studies in which the genotype frequency distribution in the control group was not in the Hardy‐Weinberg equilibrium (HWE) (Tables S4–S5). A sensitivity analysis of 37 meta‐analyses revealed that eight variants (*IL8* rs2227306, *TGF‐β1* rs1800469, *TLR2* rs3804099, *XPD* rs1799793 and rs13181, *EGF61* rs4444903, *EPHX* rs1051740, and *IL‐1B* rs16944) were no longer significantly associated with HCC risk after excluding the first published or positive report. No deviation from HWE was observed in any of the original studies included in eight of the 37 meta‐analyses, and HWE could not be calculated for some of the original studies included in six meta‐analyses. In the remaining 23 meta‐analyses, after excluding studies where the genotype frequency distributions in the control group did not conform to HWE, eight variants (*IL‐6* rs1800795, *TGF‐β1* rs1800469, *XPD* rs1799793 and rs13181, *XRCC1* rs1799782, *EPHX* rs1051740, *miR‐196a2* rs11614913, and *XRCC1* rs25487) became insignificant. In addition, studies on five variants (*HULC* rs7763881, *IL‐28B* rs12979860, *miR‐34b/C* rs4938723, *XPD* rs13181, and *XRCC1* rs25487) displayed a probable publication bias according to the results of Begg's test and Egger's test. Overall, there was generally high heterogeneity among the 37 meta‐analyses, with potential publication bias and unstable results in some studies.

### 2.7 Cumulative Epidemiological Evidence of Identified Variants

The cumulative epidemiological evidence of the 37 significant associations with HCC risk identified by meta‐analyses was ranked as strong (+++, *N* = 3), moderate (++, *N* = 9), or weak (+, *N* = 25) according to the scores of the three elements (i.e., the amount of evidence, replication, and protection from bias) of the Venice criteria [14] in the meta‐analyses (Tables 3, 4; Tables S4–S5). In the assessment of the amount of evidence, 28 meta‐analyzed variants were graded A, and the remaining 9 variants were graded B; none were graded C. For replication, 9, 17, and 11 associations were graded A, B, and C, respectively. Moreover, regarding protection from bias, 19 variants in the meta‐analyses were graded A, 2 were graded B because the HWE could not be calculated in some of the studies, and 16 were graded C. The main reason for Grade C classification in the criterion of protection from bias was potential publication bias or the inconsistent results of the sensitivity analysis.

In addition, we calculated false positive report probability (FPRP) to examine the true positive association for all variants. Eleven variants (*CCND1* rs9344, *HFE* rs1800562, *HLA‐DP* rs9277535, *IL‐6* rs1800795, *TGF‐β1* rs1800469, *XPD* rs13181, *XRCC1* rs139599857, *GSTT1* Present/Null, *EGF61* rs4444903, *miR‐196a2* rs11614913, and *XRCC1* rs25487) showed a high FPRP (> 0.2) at the prior probability level. The FPRP value of *HLA‐DQ* rs2856718 could not be calculated due to a very low *p* value (1.67 × 10^−20^).

Of the 36 variants, three (*COL18A1* rs7499, *IL1B* rs1143627, and *NQO1* rs1800566) showed “strong” credibility of cumulative epidemiological evidence with HCC and a low FPRP (< 0.2). Eight SNPs (*ESR1* rs2234693, *GRP78* rs430397, *HLA‐DP* rs3077, *HLA‐DQ* rs2856718, *MnSOD* rs4880, *TNFα* rs361525, *HFE* rs1800562, and *HFE*rs1799945) and one phenotype variant (*UGT1A7* High/Low) were predicted to have “moderate” credibility, yielding at least one Grade B and no Grade C among the three criteria. The FPRP values were low for these variants except for *HFE* rs1800562. Based on the Venice criteria, the remaining 24 variants with a Grade C were considered to have “weak” credibility due to the cumulative evidence, and only 12 of these variants had low FPRP values. Therefore, among the 36 genetic variants identified to be associated with HCC in meta‐analyses, only three variants showed strong credibility of association with HCC, and eight variants exhibited moderate evidence.

### 2.8 Enrichment Analysis and Protein‐Protein Interaction (PPI) Network Analysis

Eleven genes with 11 variants that were validated to possess moderate or strong credibility of associations with HCC risk were selected for subsequent enrichment analysis and PPI network analysis. In total, eight enriched Gene Ontology (GO) terms and two Kyoto Encyclopedia of Genes and Genomes (KEGG) pathways were identified. A PPI network of the 11 genes with 5 hub genes (*TNFα*, *IL1B*, *NQO1*, *MnSOD*, and *GRP78)* was established.

## 3 Discussion

HCC development has been linked to the incidence of multifactorial genetic predispositions [15, 16]. We conducted the most comprehensive research synopsis in the field of genetic susceptibility to HCC to date using a systematic literature search and extensive data analysis. There were 36 variants in 27 genes significantly associated with HCC in general or by ethnicity, as determined by our 369 meta‐analyses. In addition, our meta‐analyses involving at least 800 participants from both the case and control groups revealed that 25 identified variants previously discovered were not associated with HCC risk. Our findings provide reliable information on genetic variants and may be useful in exploring novel biomarkers for diagnosis and therapies of HCC.

The Venice criteria combined with FPRP revealed 11 variants with strong or moderate cumulative epidemiological evidence for a “true” association with HCC risk. Three genetic variants with low FPRP at a prior probability level showed strong evidence of associations with HCC. Two protective variants against HCC were found in the *IL‐1B* and *COL18A1* genes. The *IL‐1B* gene, which belongs to the interleukin 1 cytokine family, is an important mediator of the inflammatory response involved in tumor metastasis [17]. Some evidence suggests that the −31C allele mutation in the 5′‐UTR may reduce *IL‐1B* gene expression due to TATA box damage [18]. Thus, our finding that the *IL‐1B* gene ‐31 T>C polymorphism (rs1143627) reduces the risk of developing HCC is consistent with previous observations [18]. Another protective variant was a C>T polymorphism in the 3′‐UTR of *COL18A1* (rs7499). The *COL18A1* gene, which codes the precursor of endostatin, has been found to inhibit angiogenesis and tumor growth [19]. Xu et al. discovered that the rs7499 transition did not affect the expression of the *COL18A1* gene; hence, the potential function of rs7499 in HCC remains unknown [20]. The C609T polymorphism in *NQO1* (rs1800566, Pro187Ser) was strongly associated with an increased risk of HCC. This missense variant results in the loss of NAD(P)H quinone oxidoreductase 1 enzymatic activity, thereby increasing the risk of developing cancer [21].

Nine variants across eight genes were found to have moderate evidence of association with HCC, among which one variant showed a false‐positive association. Two variants were found in the *HLA‐DP* and *HLA‐DQ* genes, which encode classical MHCII proteins involved in antigen presentation. According to a meta‐analysis of four studies, both rs3077, a G>A polymorphism in the 3′‐UTR of the *HLA‐DP* gene, and rs2856718, an A>G polymorphism in the intergenic region of *HLA‐DQ*, were variants that reduced the risk of HCC. These results were deemed reliable although the FPRP value of rs2856718 could not be calculated due to the extremely low *p* value. Previous studies found that the A allele of rs3077 and the G allele of rs2856718 were associated with a lower risk of chronic HBV and HCV infection [22, 23, 24]. In the present study, *HLA‐DP* rs3077 also showed a protective effect on viral hepatitis‐related HCC, indicating a protective effect against HCC.

Three variants associated with a highly increased risk of HCC with a moderate level of evidence were located in the *UGT1A7*, *MnSOD*, and *ESR1* genes, respectively. The *UGT1A7* gene is a member of an enzyme superfamily referred to as UDP‐glucuronosyltransferases, which are mainly responsible for cellular defense and detoxification. Three missense variants of the *UGT1A7* gene, including N129K, R131K, and W208R, have been reported to decrease the activity of the coding enzyme [25]. Our meta‐analysis revealed that the low‐activity phenotype of *UGT1A7* was associated with an increased HCC risk in Asians than in Caucasians. The T>C polymorphism in *MnSOD* (rs4880), resulting in a valine (Val) to an alanine (Ala) transition in position ‐9 of the protein, also increased the risk of developing HCC. The *MnSOD* gene encodes an endogenous antioxidant that converts the superoxide anion to hydrogen peroxide (H_2_O_2_) in the mitochondrial matrix to allow its conversion to water and oxygen by other enzymes [26]. Since the Ala variant can achieve higher mitochondrial activity than the Val variant, *MnSOD* rs4880 is associated with an increased risk of HCC when this polymorphism produces an overload of H_2_O_2_ that damages cells [27]. PvuII (rs2234693), located on chromosome 6q25.1, is a common intronic variant of *ESR1* that regulates hepatocyte proliferation and induces hepatocarcinogenesis by mediating estrogen in the liver [28]. In contrast, according to Herrington et al., the PvuII variant may contribute to an increased expression of *ESR1* [30]. Our meta‐analyses revealed that the C allele of PvuII was associated with a 1.5‐fold increased risk of HCC in a population with a low FPRP.

With a moderate level of evidence, four genetic variants were associated with a moderate to high risk of HCC. The *TNF‐α* gene plays a role in inflammation, cellular proliferation, apoptosis, and morphogenesis [29]. In a meta‐analysis of eight studies, polymorphism converting a G allele to an A allele in the promoter region of *TNF‐α* (rs361525) increased the risk of HCC nearly 1.5‐fold. The other two variants in *TNF‐α* (rs1800629 and rs1800630) were also significantly associated with the risk of HCC. However, because of the high degree of heterogeneity among primary studies, the level of evidence was low. Our further stratified meta‐analysis revealed that TNF‐α rs1800630 greatly increased the risk of viral hepatitis‐related HCC with a moderate level of evidence and a low FPRP. In addition, these three SNPs were in very weak linkage disequilibrium (*R* ^2^ < 0.1). Another variant was located in the intron region of the *GRP78* gene (G>A, rs430397), which serves as an endoplasmic reticulum chaperone responding to endoplasmic reticulum stress and promoting tumorigenesis and progression [30]. Indeed, our primary meta‐analysis of three studies including 2057 subjects showed that the A allele mutation of the *GRP78* gene (G>A, rs430397) was associated with an increased risk of HCC.

The other two variants with moderate association with HCC risk were rs1800562 and rs1799945 missense mutations of *HFE*, denoted C282Y and H63D, respectively [31]. These two variants have been reported to result in iron overload and subsequent oxidative stress during multistep carcinogenesis [32, 33]. In our primary meta‐analysis of the H63D mutation (C>G, rs1799945), the OR (95% CI) for the GG+GC genotype compared with the CC genotype was 1.22 (1.05–1.43) (*p* = 0.012), with the FPRP value of 0.098 at a prior probability level. However, the C282Y mutation (G>A, rs1800562) was considered a false‐positive association due to an FPRP value exceeding 0.2 although the OR (95% CI) for the GG+GC genotype compared with the CC genotype was 1.28 (1.02–1.61) (*p* = 0.033).

Subsequent functional and pathway enrichment analyses demonstrated that the 11 genes primarily participate in various biological processes such as response to xenobiotic stimulus, leukocyte cell adhesion, nitric oxide biosynthesis, hormone metabolism, immune dysfunction, lipid metabolism disorder, and chronic inflammation. Previous studies have shown that persistent inflammation, aberrant lipid metabolism, and immune dysregulation, triggered by both internal and external factors, play a significant role in HCC development [1, 9]. Furthermore, sex hormones are implicated in multiple stages of HCC progression, while nitric oxide is linked to the induction of oxidative damage and fibrosis in the liver [34, 35]. These pathways may play vital roles in HCC development.

Due to the high prevalence of HCC in Asia, the majority of the studies we included were conducted on Asians. Consequently, ethnicity‐specific subgroup analysis only found three variants that showed differences in association with HCC risk compared to the overall study. Despite the implementation of ethnicity‐stratified analyses, substantial heterogeneity remained high in most meta‐analyses. However, a decrease in heterogeneity was observed in the viral‐hepatitis subgroup, suggesting that the high heterogeneity in HCC risk may be attributed to the diverse etiologies of underlying chronic liver disease, such as chronic HBV or HCV infection, heavy alcohol consumption, and metabolic dysfunction (e.g., metabolic associated fatty liver disease, MAFLD).

Although different etiologies of HCC share certain similar pathogenic mechanisms including persistent hepatic inflammation, immune dysregulation, and lipid metabolic disorders, the genetic variants and epigenetic modifications differ [36, 37]. Viral hepatitis‐related HCC, in particular, is strongly associated with proteins encoded by HBV and HCV, which regulate multiple inflammatory pathways and promote tumorigenesis [9]. Furthermore, the integration of HBV DNA into host chromosomes can result in genomic instability instigating hepatocarcinogenesis [9]. The genetic variants associated with viral hepatitis related‐HCC, alcoholic related‐HCC, and MAFLD‐related HCC should have been discerned independently. However, a significant proportion of the enrolled studies failed to specify the precise etiologies of HCC, and even in studies that specified the etiologies, other risk factors such as alcohol consumption and metabolic conditions were not segregated, making it impossible to exclusively analyze those patients. Despite insidious confounding factors unable to be adjusted, the sub‐group analyses in terms of viral hepatitis‐related HCC yielded results resembling those from the raw analyses of all the enrolled studies.

In addition, it is suggested that most HCC patients would have had one or more uncontrolled risk factors for HCC due to the difficulty of controlling all modifiable risk factors in real‐world settings. We did not assess gene‐gene or gene‐lifestyle interactions in the development of HCC, which is one of the limitations of the present study as described later. In fact, there is no consistency in the definitions of exposure variables (such as other common high‐risk factors) and use of covariates across the included studies, and thus the interaction analyses, combining results of other common high‐risk factors (such as viral infection, alcohol consumption, and others), are beyond the capacity and scope of the present study; however, further analyses are required to clarify this important issue.

In this synopsis, 32 variants were identified as having null associations with HCC. The associations between disease and genetic variants may change as sample size increases. Hence, to provide a robust finding for this study, we presented 25 variants (Table 5) that showed no association in meta‐analyses with a minimum of 1600 subjects. Future studies are not recommended to reassess these associations unless the sample size is much larger than that of our meta‐analyses.

The genetic architecture of HCC has been more clearly delineated with the rapid development of high‐throughput sequencing technologies [16, 38]. GWAS and meta‐analyses of candidate genetic association studies are reliable approaches to identifying potential disease‐associated genetic variants [40]. Zhang et al. [41] first performed GWAS on chronic HBV carriers and identified one intronic SNP in *KIF1B* (rs17401966) associated with HCC. In the past 10 years, many new susceptibility loci have been identified by GWAS for different etiologic‐related HCC [39, 40, 41]. These GWAS usually have been performed on individuals of a single ancestry; thus, future studies are encouraged that cover the whole population. Systematic meta‐analyses are essential for estimating gene‐disease associations because they provide a quantitative method for combining different research results across different populations [42, 43].

Dong et al. reported strong evidence of an association between *NQO1* rs1800566 and HCC in a meta‐analysis, which is consistent with our findings [44]. However, they did not include the FPRP analysis, which is necessary to confirm a true association. In comparison to their study, we discovered 14 new variants associated with HCC, one, five, and eight with strong, moderate, and weak evidence, respectively. Furthermore, the associations between six genetic variants and HCC risk became insignificant after increasing the sample size in our study. The strengths of our study lie in that we provided a list of GWAS‐identified variants, integrated all studies of candidate genes known to be associated with HCC, and performed updated meta‐analyses using six genetic models for all available genetic data. In addition, we reassessed the cumulative epidemiological evidence for these associations in accordance with international assessment standards [14, 45]. Therefore, our study provided a macro and systematic synopsis of noteworthy genetic variants for HCC.

Several limitations of our study should be acknowledged. Firstly, some relevant publications may have been omitted because non‐English articles and unpublished grey literature were not included. Secondly, crude ORs were used to estimate the associations instead of adjusted effect estimates, as few original studies adjusted for established risk factors. Thirdly, data were so sparse for other potential sources of heterogeneity, such as stage and etiology of HCC, that we were unable to take them into account. Fourthly, a single genetic variant does not determine liver tumorigenesis; however, gene‐gene and gene‐environment interactions were not assessed due to the paucity of individual data. Finally, as those HCC‐related SNPs were selected from descents from the included studies, the generalizability of the research findings to other populations should be estimated in future studies. Validation and prospective series are needed in order to prove clinical utility including their role evaluation in diagnosis or treatment as well.

In conclusion, our systematic meta‐analyses identified 11 variants in 11 genes with strong or moderate credibility of associations with HCC risk based on cumulative epidemiological evidence. This synopsis helps elucidate the mechanisms of carcinogenesis of HCC and provide insights into the early diagnosis and novel treatments of HCC by targeting those potential genes.

## 4 Materials and Methods

### 4.1 Literature Searches, Selection and Exclusion Criteria, and Data Extraction

The PRISMA [46] statement and Human Genome Epidemiology Network (HuGENet) [13] guidelines were adopted for the meta‐analyses performed in the present study. We used medical subject heading terms and text‐word terms to search for keywords such as “liver cancer,” “hepatocellular carcinoma,” “polymorphism,” “variation,” and “mutation.” The complete search strategy is presented in Supporting Information. The search strategy conducted involved two steps (Figure 1). In the first step, a comprehensive search of the PubMed, Embase, Web of Science, and Google Scholar databases before June 2022 was conducted. In the second step, three search strategies from June 2022 to June 2023 were used: (1) Focused monthly database searches for the names of genes associated with HCC, (2) monthly general searches for HCC, and (3) checking for the relevant meta‐analyses and review articles. Publications were screened step by step through a fast reading of the title, abstract, and full text. The inclusion criteria for our meta‐analyses were as follows: (1) HCC was pathologically diagnosed or clinical imaging features and serum alpha‐fetoprotein levels, (2) there were sufficient genetic data such as genotype frequencies for the cases and controls in the studies, and (3) the studies were required to be human case‐control or cohort studies written in English with the full text available. Known HCC susceptibility genes and GWAS‐identified variants were excluded from our meta‐analyses.

All data from the included articles were extracted independently and cross‐checked by two authors (YJ Shi and TY Lei). Disagreements were resolved by consensus or by consulting a third author (TT Ning). Data were extracted separately when several studies were included in a report. Only reports with the most extensive or recent analyses were included in case of redundant publications or overlapping data. The extracted data from eligible articles included the first author, publication year, ethnicity of the population, population source (population‐based or hospital‐based), sample size of cases and controls, gene names, variants, minor and major alleles, genotype or allelic counts, and methods of genotyping. Ethnicity was categorized as Asian, Caucasian, African, or mixed according to the study report or the primary ethnicity of the source population. The gene and variant names (rsIDs) were used following the National Center for Biotechnology Information databases unless the variant was not registered with an accession number [47].

### 4.2 Meta‐Analyses

At least three sources of data were required for each meta‐analysis. Six genetic models, including allelic, recessive, dominant, heterozygote, homozygote, and additive, were computed for genetic variants (suuporting information). Specific genotypes (e.g., based on phenotype) were used for suitable models. Ethnicity‐stratified meta‐analyses were conducted if a null association was found in the allelic model.

### 4.3 Statistical Analysis

The summary odds ratio (OR) and 95% confidence interval (CI) were used to estimate the association between genetic association models and HCC risk. According to Lu's study recommendations [48], a strong, moderate, or weak association with HCC risk was defined as ORs less than 0.67 or more than 1.5, 0.67–0.87, or 1.5–1.15, or 0.87–1.15, respectively. The Cochran Q test and *I* ^2^ statistic were used to assess the heterogeneity among studies in the meta‐analyses [49]. Meta‐analyses were performed by using the DerSimonian–Laird random‐effects model [50] if the heterogeneity between studies was significant. Otherwise, the fixed‐effects model was performed using the Mantel‐Haenszel method [51]. Expected genotype frequencies were tested by using HWE, and the bias of the results was compared with χ^2^ or Fisher's exact tests. Sensitivity analyses were performed to examine the robustness of the meta‐analyses by removing the initial positive report and studies with control groups deviating from HWE. Potential publication bias was examined by funnel plots with Begg's test and the modified Egger's test.

Statistical analyses were conducted with Stata software, version 17.0. A two‐sided *p* value of 0.05 or less was considered significant for all statistical tests.

### 4.4 Assessment of Cumulative Epidemiological Evidence

The cumulative epidemiological evidence of each meta‐analysis with a significant association was ranked as strong (+++), moderate (++), or weak (+) according to the scores of the three elements (i.e., the amount of evidence, replication, and protection from bias) of the Venice criteria [14]. The amount of evidence, replication, and protection from bias were graded as A, B, or C, as specified below. The sum of the minor alleles or less frequent genotypes in the cases and controls of the meta‐analysis was used to assess the amount of evidence, which was defined as Grades A, B, or C when the sums were greater than 1000, between 100 and 1000, or less than 100, respectively. The level of heterogeneity or replication of the association was defined as Grades A, B, or C for *I* ^2^ values of < 25%, 25%–50%, or C > 50%, respectively. The level of protection from bias was defined as Grades A, B, or C for unbiased observation, no obvious bias but lacking important information for the generation of evidence, or clearly biased or positive sensitivity analysis, respectively. A summary OR between 0.87 and 1.15 was considered Grade C. Eventually, evidence of cumulative epidemiological significant associations in the meta‐analyses was graded as strong if all three criteria met A, moderate if three criteria met A or B, and weak if any criteria met C. In addition, FPRP was calculated in the meta‐analyses to validate the association between genetic variants and HCC risk from statistically significant results (*p *< 0.05) [52]. FPRP values < 0.2 with a prior probability of 0.1 were considered to indicate significance.

### 4.5 Bioinformatics Analysis

Gene‐annotation enrichment analysis was performed by the GO annotation and the KEGG pathway with the web tool Metascape [50, 51, 52]. The results were analyzed at https://www.bioinformatics.com.cn, an online platform for data analysis and visualization. A PPI network was constructed by the STRING database (STRING combined score > 0.4) to predict protein‐protein association [53]. Genes with node degree > 4 were considered as hub genes.

## Author Contributions

**YJ Shi**, **B Huang**, **XH Liu**, **YL Liu**, **QY Hu**, **TY Lei**, **GL Xiao**, **QY Hu**, **TT Ning**, **CY Qi**: design and literature search. **YJ Shi**, **B Huang**, **X Liu**, **Y Liu**, **QY Hu**, **TY Lei**, **GL Xiao**, **TT Ning**, **LQ Tang**, **JH He**, **QL Du**, **XM Li**, **YX Zhou**: data extraction. **YJ Shi**, **LQ Tang**, **XM Li**, **YX Zhou**: data analysis. **YJ Shi**, **LQ Tang**: manuscript writing. **QF Zhang**: revised manuscript. **SQ Yue**, **HG Ren**, **H Ren**: design and supervise the study. **HG Ren**: guide the statistical analysis and manuscript editing. All authors have read and approved the final manuscript.

## Funding

This work is supported by the National Science and Technology Major Project of China (2017ZX10202203‐007).

## Ethics Statement

The authors have nothing to report.

## Conflicts of Interest

The authors declare no conflicts of interest.

## Supporting information




**Figure S1**. Forest plot of additional genetic variants significantly associated with risk of hepatocellular carcinoma as confirmed in meta‐analyses in the recessive or dominant model.
**Table S1**. Associations of 68 genetic variants with HCC risk in meta‐analyses using different genetic models.
**Table S2**. Included studies for the genetic variants showing a statistically significant association with risk of HCC from all available meta‐analyses.
**Table S3**. Detailed information for genetic variants significantly associated with HCC risk in meta‐analyses for all available data.
**Table S4**. Detailed information for additional genetic variants significantly associated with HCC risk in meta‐analyses using other models.
**Table S5**. Meta‐analyses of associations between 25 genetic variants and HCC risk by ethnicity.
**Table S6**. Previously published meta‐analyses compared to current study in our study.

## Data Availability

All data were collected from publicly available literatures, and all data generated or analyzed during this study are included in this published article and its supplemental materials.
