# Supplementary material for: Genetic Association Studies in Hepatocellular Carcinoma: Systematic Meta‐Analyses, a Comprehensive Field Synopsis, and Epidemiological Evidence
Source: MedComm (2020). 2026 Aug 26;7(9):e70153. doi: 10.1002/mco2.70153 (PMC13507827; doi:10.1002/mco2.70153)
Supplement: Supplementary file 1 [file MCO2-7-e70153-s001.pdf]

Genetic association studies in hepatocellular carcinoma: Systematic meta-analyses, a comprehensive field synopsis, and epidemiological evidence

Running title: Genetic variants in hepatocellular carcinoma

Yujin Shi[#1], Qinyong Hu[#2], Tianyu Lei[#2], Luqi Tang[1], Guanglin Xiao[1], Tingting Ning[2], Shengqin Yue[2], Jiahui He[2], Qilian Du[2], Xuanmei Li[1], Yixuan Zhou[1], Qiongfang Zhang[1], Hong Ren*[1], Hong-Gang Ren*[3]

Affiliations:

[1]Department of Infectious Diseases, Key Laboratory of Molecular Biology for Infectious Diseases (Ministry of Education), Institute for Viral Hepatitis, The Second Affiliated Hospital, Chongqing Medical University, Chongqing, China

[2]Department of Oncology, Renmin Hospital of Wuhan University, Wuhan, China

[3]Chongqing Medical University, Chongqing, China

Corresponding Authors:

Professors Hong-Gang Ren

E-mail: honggang_ren@163.com, or hren@cqmu.edu.cn

and Hong Ren, MD, PhD,

E-mail: renhong0531@cqmu.edu.cn

Address: Department of Infectious Diseases, Key Laboratory of Molecular Biology for Infectious Diseases (Ministry of Education), Institute for Viral Hepatitis, the Second Affiliated Hospital, Chongqing Medical University, 288 Tianwen Avenue,

Nan'an District, Chongqing, China, 401336.

Note: #Yujin Shi, Qinyong Hu, and Tianyu Lei contributed equally to this study.

Supplemental materials

Catalogue

1.Search strategy

2.Supplemental Table 1. Associations of 68 genetic variants with HCC risk in meta-analyses using different genetic models.

3.Supplemental Table 2. Included studies for the genetic variants showing a statistically significant association with risk of HCC from all available meta-analyses.

4.Supplemental Table 3. Detailed information for genetic variants significantly associated with HCC risk in meta-analyses for all available data.

5.Supplemental Table 4. Detailed information for additional genetic variants significantly associated with HCC risk in meta-analyses using other models.

6.Supplemental Table 5. Meta-analyses of associations between 25 genetic variants and HCC risk by ethnicity.

7.Supplemental Table 6. Previously published meta-analyses compared to current study in our study.

8.Reference in Supplemental Table 2

9.References for previous meta-analyses

10. PRISMA checklist

11. Forest plot of meta-analysis in Table 3

12. Figure S1 Forest plot of additional genetic variants significantly associated with risk of hepatocellular carcinoma as confirmed in meta-analyses in the recessive or dominant model.

1. Search strategy

The following medical subject heading (MeSH) terms and text-word terms were used in **PubMed, Web of Science and Google Scholar**:

MeSH:

Liver neoplasms/ carcinoma, hepatocellular/ polymorphism, genetic/ genetic variation/ mutation

Keywords:

Liver neoplasm/ liver cancer/ liver carcinoma / liver cell carcinoma/ hepatic neoplasm/ hepatic cancer/ hepatic carcinoma/ hepatoma/ hepatocellular cancer/ hepatocellular carcinoma/ variant/ genetic diversity/ mutations/ polymorphism/ genetic polymorphism/ gene polymorphism

The following strategy was used in **Embase**:

Exp: liver cancer/ liver cell carcinoma/ genetic polymorphism/ genetic variation/ mutation

Keywords:

Liver neoplasm/ liver carcinoma/ liver cell carcinoma/ hepatic neoplasm/ hepatic cancer/ hepatic carcinoma/ hepatoma/ hepatocellular cancer/ hepatocellular carcinoma/ variant/ genetic diversity/ mutations/ polymorphism /genetic polymorphism/ gene polymorphism

2.Supplemental Table 1. Associations of 68 genetic variants with HCC risk in meta-analyses using different genetic models.

NO	Genes	Variants	Category	Comparison[a]	Ethnicity	Frequency (%)[b]	Studies	Cases	Controls	Allele model		Heterogeneity		Recessive model		Heterogeneity		Dominant model		Heterogeneity		Homozygote model		Heterogeneity		Heterozygote model		Heterogeneity		Additive model		Heterogeneity	
										OR(95%CI)	p value	p value	I²	OR(95%CI)	p value	p value	I²	OR(95%CI)	p value	p value	I²	OR(95%CI)	p value	p value	I²	OR(95%CI)	p value	p value	I²	OR(95%CI)	p value	p value	I²
1	α1AT	MM/non-MM	Phenotype	non-MM vs MM	Caucasians	8.34	3	251	2375	1.32(0.83-2.11)	0.238	0.307	15.40%																				
2	ALDH2	rs671(Glu487Lys)	Missense	A vs G	Asian	27.45	10	2153	5051	1.06(0.90-1.24)	0.494	0.009	60.80%	1.10(0.89-1.35)	0.383	0.388	5.50%	1.10(0.91-1.33)	0.347	0.009	59.20%	1.10(0.89-1.36)	0.391	0.233	24.50%	1.05(0.87-1.28)	0.603	0.024	54.80%	0.98(0.88-1.10)	0.774	0.044	49.60%
3	BIRC5	rs9904341(-31G>C)	5'UTR	C vs G	All	45.09	4	533	963	0.85(0.73-1.00)	0.052	0.715	0.00%	0.79(0.59-1.05)	0.103	0.621	0.00%	0.83(0.65-1.05)	0.122	0.651	0.00%	0.75(0.53-1.05)	0.088	0.692	0.00%	0.86(0.67-1.11)	0.249	0.569	0.00%	1.02(0.81-1.27)	0.896	0.328	12.90%
4	CCND1	rs9344(G870A)	Synonymous Variant	G vs A	All	48.13	5	805	1255	0.69(0.50-0.94)	0.020	0.001	79.90%	0.61(0.40-0.93)	0.023	0.019	66.10%	0.64(0.45-0.91)	0.012	0.036	61.00%	0.50(0.29-0.86)	0.013	0.007	71.80%	0.76(0.61-0.95)	0.014	0.439	0.00%	1.00(0.83-1.21)	0.970	0.761	0.00%
5	COL18A1	rs7499	3'UTR	T vs C	Asian	47.23	3	808	1067	0.73(0.64-0.83)	2.32×10^{-6}	0.784	0.00%	0.56(0.44-0.70)	7.31×10^{-7}	0.697	0.00%	0.74(0.61-0.91)	0.005	0.948	0.00%	0.51(0.39-0.67)	1.46×10^{-6}	0.764	0.00%	0.87(0.70-1.08)	0.199	0.951	0.00%	0.86(0.72-1.03)	0.109	0.556	0.00%
6	COX-2	rs20417(G-765C)	2KB Upstream	C vs G	All	12.44	4	847	857	0.99(0.55-1.79)	0.981	0	87.00%	0.90(0.17-4.88)	0.906	0.007	79.90%	1.03(0.58-1.85)	0.917	0	83.10%	0.94(0.16-5.35)	0.940	0.005	81.00%	1.08(0.64-1.82)	0.781	0.004	77.70%	0.90(0.54-1.50)	0.688	0.005	76.30%
7	COX-2	rs689466	2KB Upstream	A vs G	All	46.14	5	1537	1547	1.15(0.95-1.39)	0.166	0.03	62.70%	1.08(0.92-1.27)	0.335	0.129	43.90%	1.16(0.97-1.37)	0.100	0.101	48.40%	1.40(0.92-2.14)	0.113	0.029	63.10%	1.14(0.95-1.37)	0.155	0.36	8.10%	0.96(0.84-1.11)	0.611	0.988	0.00%
8	CTLA4	rs231775	Missense	A vs G	Asian	32.98	5	1971	2464	1.00(0.76-1.30)	0.982	0	85.80%	1.13(0.75-1.70)	0.572	0.006	72.50%	0.97(0.71-1.32)	0.824	0	80.80%	1.08(0.64-1.83)	0.772	0	81.40%	0.95(0.74-1.22)	0.700	0.02	65.80%	1.05(0.93-1.19)	0.412	0.462	0.00%
9	CYP1A1	rs4646903(MspI site)	500B Downstream	C vs T	All	40.9	4	695	871	1.17(0.79-1.74)	0.433	0.005	76.30%	0.87(0.68-1.11)	0.258	0.333	12.00%	1.48(0.82-2.68)	0.196	0.006	75.70%	1.15(0.66-2.01)	0.623	0.085	54.70%	1.51(0.86-2.66)	0.150	0.018	70.10%	0.75(0.50-1.11)	0.146	0.074	56.80%
10	CYP2C19	PM/EM	Phenotype	EM vs PM	Asian	45.43	3	89	1093	1.44(0.89-2.34)	0.140	0.18	41.70%																				
11	CYP2E1	rs2031920	2KB Upstream Variant	T vs C	All	14.11	11	1297	2355	0.99(0.71-1.39)	0.952	0.012	59.20%	0.79(0.50-1.25)	0.316	0.599	0.00%	1.01(0.85-1.20)	0.943	0.04	47.90%	0.80(0.51-1.26)	0.329	0.553	0.00%	1.02(0.84-1.25)	0.830	0.044	49.70%	0.96(0.79-1.17)	0.690	0.054	47.70%
12	DNMT3B	rs2424913(C-149T)	Intron	T vs C	All	21.77	3	304	578	0.96(0.69-1.34)	0.813	0.273	23.00%	0.86(0.48-1.55)	0.616	0.267	24.30%					NA				NA				0.90(0.58-1.41)	0.647	0.269	23.80%
13	EGF61	rs4444903	5'UTR	A vs G	All	46.52	16	1943	3505	1.26(1.00-1.60)	0.052	0.052	83.50%	1.32(0.91-1.91)	0.147	0.147	79.50%	1.32(1.02-1.70)	0.038	0	66.20%	1.52(0.95-2.43)	0.085	0.085	79.50%	1.11(0.96-1.28)	0.150	0.187	24.10%	1.00(0.88-1.12)	0.936	0.281	15.70%
14	EPHX	rs1051740	Missense	C vs T	All	31.21	11	816	1780	1.21(0.87-1.67)	0.253	0	71.00%	1.48(1.10-2.01)	0.012	0.214	24.90%	1.28(0.86-1.89)	0.218	0	74.80%	1.53(0.85-2.76)	0.158	0.013	57.10%	1.06(0.83-1.35)	0.627	0.057	45.50%	1.06(0.84-1.32)	0.634	0.531	0.00%
15	ESR1	rs2234693(PvuII)	Intron	C vs T	All	43.16	3	367	458	1.51(1.22-1.86)	1.40×10^{-4}	0.145	48.30%	1.75(1.18-2.59)	0.005	0.301	16.60%	1.68(1.24-2.28)	9.24×10^{-4}	0.189	40.00%	2.25(1.44-3.51)	3.79×10^{-4}	0.353	3.90%	1.51(1.09-2.08)	0.014	0.191	39.60%	0.85(0.64-1.14)	0.286	0.168	44.00%
16	ESR1	rs9340799(XbaI)	Intron	G vs A	All	20.34	3	367	458	1.24(0.97-1.58)	0.0826	0.896	0.00%	1.50(0.81-2.77)	0.198	0.297	17.60%	1.26(0.93-1.60)	0.136	0.893	0.00%	1.61(0.86-3.03)	0.139	0.387	0.00%	1.20(0.88-1.64)	0.251	0.675	0.00%	0.87(0.64-1.18)	0.373	0.519	0.00%
17	GRP78	rs430397	Intron	A vs G	All	15.7	3	1011	1046	1.42(1.20-1.69)	5.66×10^{-5}	0.552	0.00%	1.79(1.04-3.09)	0.035	0.741	0.00%	1.46(1.20-1.78)	1.37×10^{-4}	0.461	0.00%	1.94(1.12-3.36)	0.018	0.752	0.00%	1.42(1.16-1.74)	0.001	0.359	2.50%	0.73(0.59-0.89)	0.002	0.288	19.60%
18	GSTP1	rs1695(313A>G)	Missense	G vs A	All	28.36	7	1143	1444	1.45(1.08-1.95)	0.014	0.002	73.80%	1.50(0.71-3.18)	0.291	0.022	61.80%	1.40(0.99-1.96)	0.051	0.002	70.90%	1.71(0.79-3.71)	0.173	0.021	62.20%	1.46(1.08-1.98)	0.014	0.037	57.80%	0.72(0.53-0.98)	0.036	0.026	60.70%
19	GSTM1	Present/Null	Phenotype	null vs present	All	49.80	24	4209	6971	1.13(0.92-1.39)	0.238	0	82.90%																				
20	GSTT1	Present/Null	Phenotype	null vs present	All	37.78	20	3801	6060	1.21(0.99-1.46)	0.052	0	74.40%																				
21	HFE	rs1800562(Cys282Tyr)	Missense	A vs G	All	5.13	14	1221	3334	1.28(1.02-1.61)	0.033	0.02	49.00%	1.92(0.47-7.89)	0.364	0.014	64.80%	1.20(0.94-1.54)	0.144	0.07	39.30%	1.89(0.45-7.97)	0.386	0.012	66.00%	1.11(0.85-1.44)	0.441	0.139	29.80%	0.91(0.70-1.19)	0.502	0.147	28.90%
22	HFE	rs1799945(187C>G)	Missense	G vs C	All	15.21	16	1282	2468	1.15(0.91-1.45)	0.247	0.008	53.40%	1.11(0.67-1.84)	0.675	0.192	26.50%	1.22(1.05-1.43)	0.012	0.03	44.10%	1.22(0.74-2.02)	0.432	0.122	34.60%	1.23(1.04-1.45)	0.017	0.081	36.00%	0.82(0.70-0.97)	0.019	0.151	27.80%
23	HLA-DPA1	rs3077	3'UTR	A vs G	All	35.51	4	1865	3113	0.77(0.70-0.84)	3.38×10^{-8}	0.172	40.00%	0.77(0.63-0.94)	0.011	0.662	0.00%	0.70(0.62-0.79)	1.15×10^{-8}	0.107	47.40%	0.63(0.51-0.79)	4.77×10^{-5}	0.467	0.00%	0.69(0.53-0.90)	0.006	0.058	60.00%	1.29(1.14-1.46)	5.83×10^{-5}	0.139	45.40%
24	HLA-DP	rs7453920	Intron	GG vs GA+AA	All	23.07	3	1409	1946					0.96(0.62-1.51)	0.873	0.116	53.60%																
25	HLA-DP	rs9277535	3'UTR	A vs G	All	41.25	6	2238	4067	0.79(0.64-0.99)	0.0362	0	81.30%	0.83(0.62-1.10)	0.189	0.019	63.00%	0.66(0.49-0.88)	0.004	0	75.80%	0.65(0.42-0.99)	0.045	0	77.80%	0.70(0.55-0.90)	0.005	0.013	65.60%	1.25(1.12-1.40)	6.00×10^{-5}	0.417	0.00%
26	HLA-DQ	rs2856718	Intergenic	G vs A	All	43.24	4	1974	2831	0.67(0.61-0.73)	1.67×10^{-20}	0.126	47.60%	0.63(0.54-0.74)	4.14×10^{-9}	0.203	34.80%	0.58(0.51-0.65)	4.26×10^{-1}	0.20	35.50%	0.48(0.40-0.57)	3.37×10^{-1}	0.157	42.40%	0.62(0.54-0.71)	3.11×10^{-1}	0.341	10.40%	1.24(1.10-1.40)	3.87×10^{-4}	0.721	0.00%
27	HLA-G	rs371194629	Insertions/deletion	Ins vs del	All	28.7	5	1051	1397	1.02(0.76-1.37)	0.913	0.001	78.90%	1.02(0.63-1.67)	0.926	0.017	66.70%	0.99(0.72-1.37)	0.966	0.017	66.80%	1.02(0.57-1.86)	0.938	0.005	73.00%	0.92(0.76-1.11)	0.377	0.176	36.80%	1.10(0.92-1.30)	0.299	0.545	0.00%
28	HOGG1	rs1052133(Ser326Cys)	Missense	G vs C	All	48.07	7	1069	1703	1.15(0.87-1.52)	0.336	0	81.00%	1.13(0.72-1.78)	0.597	0	79.70%	1.40(0.96-2.05)	0.082	0.002	70.90%	1.54(0.89-2.68)	0.124	0	77.40%	1.39(0.98-1.96)	0.066	0.016	61.50%	0.82(0.62-1.10)	0.185	0.01	64.50%
29	HULC	rs7763881	Intron Variant	C vs A	All	47.56	3	1887	2222	0.90(0.82-0.98)	0.0149	0.278	21.90%	0.97(0.83-1.12)	0.636	0.443	0.00%	0.79(0.68-0.90)	6.38×10^{-4}	0.378	0.00%	0.82(0.68-0.98)	0.025	0.293	18.60%	0.77(0.67-0.89)	0.001	0.541	0.00%	1.18(1.05-1.34)	0.008	0.799	0.00%
30	IL-1B	rs1143627(-31T>C)	5'UTR	C vs T	Asian	46.11	7	1331	1409	0.83(0.74-0.92)	0.001	0.23	26.00%	0.85(0.71-1.03)	0.098	0.102	43.40%	0.72(0.61-0.86)	1.90×10^{-4}	0.62	0.00%	0.70(0.56-0.87)	0.001	0.158	35.40%	0.73 (0.61-0.88)	0.001	0.718	0.00%	1.18(1.01-1.37)	0.041	0.593	0.00%
31	IL-1B	rs16944	Intron	T vs C	All	47.82	5	949	974	0.90(0.66-1.21)	0.468	0.005	73.30%	1.03(0.62-1.74)	0.900	0.002	76.00%	0.78(0.63-0.97)	0.0239	0.14	42.30%	0.75(0.40-1.40)	0.367	0.01	70.10%	0.75(0.60-0.95)	0.015	0.292	19.20%	1.27(1.06-1.54)	0.012	0.156	39.70%
32	IL-6	rs1800795	Intron	C vs G	All	49.16	5	436	843	0.63(0.42-0.95)	0.0253	0.109	54.90%	0.33(0.14-0.76)	0.010	0.381	0.00%	0.61(0.38-0.97)	0.0384	0.029	66.90%	0.32(0.13-0.75)	0.009	0.527	0.00%	0.69(0.30-1.58)	0.379	0.003	83.00%	1.31(0.52-3.29)	0.562	0.001	86.40%
33	IL-6	rs1800796	Intron	G vs C	All	47.13	7	1664	1913	1.02(0.82-1.26)	0.893	0.038	55.10%	0.94(0.64-1.39)	0.754	0.01	64.20%	1.03(0.86-1.22)	0.766	0.423	0.00%	0.84(0.60-1.17)	0.300	0.395	3.30%	1.21(0.79-1.87)	0.380	0.024	61.40%	0.87(0.61-1.25)	0.451	0.001	74.40%
34	IL8	rs4073(-251T>A)	2KB Upstream	A vs T	All	41.74	5	1052	1249	1.07(0.86-1.32)	0.545	0.025	64.10%	0.82(0.65-1.03)	0.09	0.164	38.60%	1.24(0.82-1.89)	0.306	0.001	79.80%	1.04(0.81-1.35)	0.750	0.111	46.70%	1.31(0.83-2.07)	0.241	0	81.10%	0.77(0.52-1.14)	0.193	0.001	79.70%
35	IL8	rs2227306(+781C>T)	Intron	T vs C	Asian	36.4	3	486	698	0.78(0.66-0.93)	0.006	0.176	42.40%	0.67(0.34-1.35)	0.261	0.047	67.20%	0.76(0.60-0.97)	0.027	0.34	6.60%	0.60(0.31-1.19)	0.143	0.074	61.50%	0.82(0.63-1.05)	0.115	0.340	7.20%	1.08(0.85-1.37)	0.526	0.181	41.60%
36	IL10	rs1800872(-592A>C)	2KB Upstream	C vs A	All	44.91	9	2609	3405	1.04(0.84-1.28)	0.722	0	81.90%	1.14(0.90-1.44)	0.295	0.047	50.80%	1.04(0.78-1.39)	0.797	0	79.90%	1.23(0.86-1.74)	0.257	0.002	68.50%	1.03(0.77-1.39)	0.835	0	77.90%	1.02(0.84-1.25)	0.837	0.008	63.40%
37	IL18	rs187238(-137G>C)	Intron	C vs G	All	15.09	6	740	1808	0.99(0.62-1.57)	0.963	0	82.20%	0.80(0.45-1.43)	0.457	0.19	32.80%	1.02(0.59-1.77)	0.946	0	82.70%	0.81(0.45-1.46)	0.484	0.121	42.70%	1.05(0.60-1.82)	0.872	0	81.10%	0.94(0.55-1.60)	0.824	0	80.10%
38	IL18	rs1946518(-607A>C)	2KB Upstream Variant	C vs A	All	49.88	6	764	1319	0.90(0.69-1.18)	0.446	0.006	69.50%	0.93(0.75-1.15)	0.492	0.686	0.00%	0.80(0.45-1.44)	0.456	0	80.60%	0.72(0.40-1.31)	0.287	0.005	70.30%	0.82(0.45-1.480	0.501	0.001	79.20%	1.02(0.74-1.40)	0.905	0.048	55.20%
39	IL-28B	rs12979860	Intron	T vs C	All	33.85	17	4267	4862	1.30(1.08-1.58)	0.007	0	70.30%	1.42(1.04-1.95)	0.028	0.009	53.40%	1.53(1.18-1.97)	0.001	0.00	75.70%	1.61(1.10-2.34)	0.014	0.003	58.60%	1.13(0.99-1.29)	0.069	0.114	32.60%	0.96(0.85-1.08)	0.488	0.439	0.90%
40	IL-28B	rs8099917	upstream variant	G vs T	All	17.99	13	3864	4156	1.08(0.95-1.23)	0.245	0.153	36.10%	1.01(0.74-1.39)	0.951	0.705	0.00%	1.33(1.06-1.68)	0.014	0.00	58.70%	1.02(0.74-1.42)	0.884	0.709	0.00%	1.12(0.95-1.33)	0.162	0.372	7.40%	0.89(0.76-1.05)	0.155	0.365	8.40%
41	leptin receptor	rs1137101(Gln233Arg)	Missense	A vs G	Asian	24.08	3	1141	1617	1.16(0.76-1.76)	0.488	0.001	85.80%	1.02(0.40-2.60)	0.972	0.1	56.60%	1.26(0.74-2.13)	0.397	0.001	86.10%	1.08(0.31-3.80)	0.901	0.023	73.60%	1.23(0.81-1.88)	0.334	0.014	76.50%	0.94(0.79-1.12)	0.492	0.705	0.00%
42	MALAT1	rs619586	Non Coding Transcript	G vs A	All	8.24	5	2872	4023	0.95(0.84-1.08)	0.449	0.256	24.80%	0.64(0.37-1.11)	0.113	0.434	0.00%	0.97(0.85-1.11)	0.688	0.308	16.80%	0.64(0.37-1.11)	0.112	0.417	0.00%	1.00(0.87-1.14)	0.948	0.362	7.90%	1.00(0.87-1.15)	0.993	0.375	5.60%
43	MBOAT7	rs641738	500B Downstream	T vs C	All	43.47	3	1413	1903	1.27(0.96-1.68)	0.098	0.018	75.10%	1.17(0.97-1.41)	0.103	0.45	0.00%	1.41(0.89-2.23)	0.139	0.008	79.30%	1.53(0.88-2.67)	0.136	0.041	68.80%	1.36(0.86-2.14)	0.185	0.014	76.70%	0.87(0.65-1.17)	0.359	0.091	58.20%
44	MDM2	rs2279744(T309G)	Intron	G vs T	All	47.88	12	3011	3219	1.39(1.22-1.60)	1.33×10^{-6}	0.001	63.80%	1.33(1.19-1.49)	5.34×10^{-7}	0.237	20.40%	1.67(1.37-2.04)	3.32×10^{-7}	0.008	55.20%	1.96(1.52-2.53)	1.98×10^{-7}	0.005	57.40%	1.43(1.26-1.62)	3.52×10^{-8}	0.101	35.20%	0.93(0.84-1.03)	0.162	0.922	0.00%
45	MTHFR	rs1801131(A1298C)	Missense	C vs A	All	20.59	8	1447	2664	0.96(0.75-1.23)	0.732	0	77.10%	0.65(0.33-1.25)	0.196	0	74.00%	1.00(0.79-1.26)	0.978	0.008	63.50%	0.66(0.33-1.31)	0.230	0	75.30%	1.02(0.88-1.18)	0.784	0.15	34.90%	0.95(0.82-1.10)	0.321	0.465	13.90%
46	MTHFR	rs1801133	Missense	T vs C	Asian	41.88	17	4973	6693	1.01(0.89-1.15)	0.887	0	78.40%	1.00(0.84-1.19)	0.966	0.003	55.60%	1.01(0.84-1.22)	0.901	0	76.10%	1.03(0.80-1.31)	0.838	0	70.40%	1.00(0.84-1.18)	0.966	0	68.30%	1.00(0.93-1.08)	0.956	0.01	49.80%
47	miR-146a	rs2910164	Non Coding Transcript	C vs G	All	44.61	21	5728	7021	1.01(0.91-1.11)	0.919	0	65.90%	1.00(0.88-1.13)	0.947	0.003	52.10%	1.01(0.87-1.17)	0.921	0.003	52.70%	1.01(0.83-1.23)	0.93	0	60.00%	1.03(0.93-1.14)	0.604	0.105	29.00%	0.96(0.90-1.03)	0.285	0.521	0.00%
48	miR-196a2	rs11614913	Non Coding Transcript	C vs T	All	48.34	17	4992	5910	1.08(0.97-1.21)	0.147	0	70.60%	1.08(0.91-1.29)	0.397	0	68.40%	1.18(1.00-1.39)	0.047	0	63.10%	1.25(0.99-1.58)	0.065	0	70.90%	1.16(1.00-1.35)	0.053	0.007	51.80%	0.96(0.89-1.04)	0.291	0.032	42.90%
49	miR-34b/C	rs4938723	2KB Upstream	C vs T	Asian	33	6	3134	3624	1.23(1.06-1.44)	0.008	0.001	74.70%	1.18(0.83-1.68)	0.369	0	81.50%	1.24(1.06-1.44)	0.007	0.016	61.40%	1.32(0.89-1.97)	0.170	0	83.50%	1.18(1.07-1.29)	0.001	0.226	26.50%	0.87(0.80-0.96)	0.003	0.591	0.00%
50	miR-499	rs3746444	Intron	G vs A	All	23.90	15	2816	3771	1.14(0.94-1.37)	0.189	0	79.20%	1.10(0.83-1.46)	0.513	0.011	51.20%	1.20(0.96-1.51)	0.118	0.00	76.20%	1.21(0.85-1.74)	0.288	0	63.90%	1.20(0.97-1.49)	0.098	0.000	69.50%	0.86(0.72-1.03)	0.098	0.002	59.40%
51	miR-499	rs2292832	Non Coding Transcript	C vs T	All	45.07	7	1429	2068	0.95(0.86-1.06)	0.357	0.089	45.40%	0.93(0.79-1.10)	0.397	0.263	21.80%	0.91(0.78-1.07)	0.269	0.31	15.60%	0.87(0.71-1.08)	0.219	0.185	31.80%	0.93(0.79-1.10)	0.407	0.564	0.00%	1.02(0.89-1.18)	0.772	0.649	0.00%
52	MnSOD	rs4880(Val-9Ala)	Missense	C vs T	Caucasian	48.58	3	238	360	1.82(1.43-2.32)	1.45×10^{-6}	0.474	0.00%	2.18(1.48-3.20)	7.71×10^{-6}	0.626	0.00%	2.13(1.42-3.19)	3.23×10^{-4}	0.57	0.00%	3.07(1.90-4.97)	5.14×10^{-6}	0.457	0.00%	1.73(1.12-2.67)	0.014	0.763	0.00%	1.03(0.73-1.45)	0.859	0.866	0.00%
53	NAT2	Rapid/Slow	Phenotype	Slow vs rapid	All	43.28	7	1055	1302	0.99(0.81-1.20)	0.890	0.259	22.30%																				
54	NQO1	rs1800566	Missense	T vs C	All	40.70	3	740	765	1.25(1.08-1.45)	0.003	0.676	0.00%	1.37(1.05-1.78)	0.022	0.896	0.00%	1.31(1.06-1.63)	0.013	0.55	0.00%	1.53(1.13-2.07)	0.006	0.71	0.00%	1.24(0.98-1.55)	0.069	0.535	0.00%	0.94(0.77-1.15)	0.552	0.456	0.00%
55	TGFβ1	rs1800469	2KB Upstream	T vs C	All	46.42	9	2160	3255	1.24(1.02-1.50)	0.030	0	79.40%	1.24(0.97-1.60)	0.090	0.003	65.20%	1.26(0.94-1.67)	0.119	0	78.80%	1.53(1.03-2.26)	0.036	0	78.80%	1.27(0.99-1.62)	0.059	0.012	59.40%	0.95(0.85-1.06)	0.338	0.706	0.00%
56	TNFα	rs1799724	2KB Upstream	T vs C	All	16.73	7	1334	2115	0.98(0.85-1.13)	0.783	0.874	0.00%	1.02(0.69-1.51)	0.930	0.613	0.00%	1.05(0.90-1.23)	0.508	0.217	27.80%	1.03(0.69-1.54)	0.882	0.681	0.00%	0.97(0.81-1.15)	0.695	0.522	0.00%	1.03(0.87-1.23)	0.721	0.441	0.00%
57	TNFα	rs1800629	2KB Upstream	A vs G	All	11.63	18	2125	3663	1.58(1.18-2.12)	0.002	0	74.50%	1.96(1.36-2.82)	2.82×10^{-4}	0.181	24.10%	1.69(1.28-2.23)	1.86×10^{-4}	0	71.30%	2.20(1.51-3.20)	4.50×10^{-5}	0.066	37.40%	1.58(1.15-2.17)	0.005	0	68.80%	0.67(0.49-0.90)	0.008	0	67.10%
58	TNFα	rs1800630(-863C>A)	2KB Upstream	A vs C	All	18.78	10	1994	2957	1.34(1.12-1.61)	0.002	0.022	55.20%	1.53(1.15-2.04)	0.003	0.609	0.00%	1.43(1.13-1.82)	0.003	0.00	62.60%	1.64(1.23-2.20)	0.001	0.43	0.40%	1.45(1.09-1.92)	0.010	0.001	70.40%	0.72(0.55-0.94)	0.016	0.001	68.90%
59	TNFα	rs361525	2KB Upstream	A vs G	All	12.02	8	763	1175	1.49(1.16-1.91)	0.002	0.128	37.80%	2.67(1.17-6.10)	0.020	0.873	0.00%	1.26(0.83-1.91)	0.284	1.914	50.70%	4.26(1.44-12.57)	0.009	0.83	0.00%	1.29(0.78-2.11)	0.322	0.027	55.70%	0.82(0.52-1.29)	0.393	0.039	52.70%
60	TLR2	rs3804099	Synonymous	C vs T	All	40.29	4	1213	2599	1.33(1.05-1.69)	0.019	0.004	77.90%	1.70(1.41-2.05)	1.92×10^{-8}	0.718	0.00%	1.04(0.61-1.77)	0.898	0	87.60%	1.38(0.85-2.26)	0.197	0.016	71.10%	0.84(0.45-1.57)	0.584	0	89.90%	1.30(0.75-2.25)	0.351	0	92.80%
61	UGT1A7	High/Low	Phenotype	Low vs High	All	54.08	7	521	936	1.48(0.72-3.07)	0.287	0	88.10%																				
62	XRCC1	rs139599857	Missense	C vs G	All	35.04	3	761	733	2.61(1.20-5.69)	0.016	0	89.30%	2.90(1.04-8.10)	0.042	0.014	76.60%	2.98(1.32-6.72)	0.009	0.006	80.60%	4.42(1.30-15.06)	0.018	0.006	80.30%	2.13(1.24-3.67)	0.006	0.122	52.50%	0.72(0.58-0.88)	0.001	0.288	19.60%
63	XRCC1	rs1799782(Arg194Trp)	Missense	G vs C	All	24.24	8	1259	1385	1.14(1.00-1.31)	0.047	0.101	43.40%	0.97(0.57-1.64)	0.904	0.025	58.40%	1.26(1.06-1.48)	0.007	0.598	0.00%	1.23(0.89-1.68)	0.209	0.259	22.30%	1.25(1.05-1.50)	0.014	0.33	13.10%	0.78(0.66-0.93)	0.005	0.095	44.40%
64	XRCC1	rs25487(Arg399Gln)	Missense	G vs A	All	28.85	25	5189	6362	1.18(0.99-1.41)	0.062	0	83.90%	1.31(1.01-1.71)	0.044	0	68.10%	1.17(0.94-1.44)	0.154	0	81.50%	1.26(0.90-1.77)	0.183	0	76.50%	1.13(0.90-1.40)	0.291	0	78.00%	0.92(0.76-1.11)	0.368	0	75.40%
65	XRCC1	rs25489(Arg280His)	Missense	A vs G	All	16.69	7	913	1063	1.19(0.85-1.68)	0.308	0.007	68.90%	1.21(0.80-1.83)	0.372	0.209	30.20%	1.13(0.72-1.78)	0.595	0.001	74.30%	1.44(0.92-2.23)	0.108	0.168	35.90%	1.22(0.69-2.15)	0.499	0	80.80%	0.87(0.51-1.50)	0.625	0	80.20%
66	XRCC3	rs861539(Thr241Met)	Missense	T vs C	All	24.22	5	2624	3532	1.34(0.83-2.14)	0.229	0	95.10%	4.07(1.22-13.56)	0.022	0	93.90%	1.63(0.78-3.41)	0.194	0	96.50%	4.54(1.11-18.49)	0.035	0	95.20%	1.37(0.73-2.55)	0.326	0	94.40%	0.85(0.56-1.29)	0.452	0	88.20%
67	XPD	rs1799793(Asp312Asn)	Missense	T vs C	Asian	22.93	4	1500	1650	1.20(1.06-1.36)	0.006	0.71	0.00%	1.27(0.98-1.65)	0.077	0.821	0.00%	1.24(1.07-1.44)	0.004	0.72	0.00%	1.33(1.02-1.74)	0.037	0.787	0.00%	1.17(0.98-1.40)	0.076	0.919	0.00%	0.89(0.75-1.05)	0.163	0.958	0.00%
68	XPD	rs13181(Lys751Gln)	Nonsense	G vs T	All	27.61	8	3457	3940	1.36(1.01-1.83)	0.042	0	90.60%	1.86(1.24-2.79)	0.003	0.001	72.40%	1.48(1.04-2.10)	0.030	0.00	88.80%	2.27(1.43-3.62)	0.001	0	77.10%	1.42(1.05-1.92)	0.022	0.000	81.90%	0.78(0.64-0.94)	0.009	0.021	57.50%

OR=odds ratio, CI= confidence interval, C=cytosine, G=guanine, A=adenine, T=thymine.

[a]Allelic contrast or phenotype for common variants, or genetic comparison with only genotype group data.

[b]Frequency of minor allele or effect genotype.

3.Supplemental Table 2. Included studies for the genetic variants showing a statistically significant association with risk of HCC from all available meta-analyses

NO	Gene	Variant	Ethnicity	Studies	References
29 genes in Table 3					
1	COL18A1	rs7499	Asian	3	[1]
2	CCND1	rs9344	All	5	[2-6]
3	ESR1	rs2234693	All	3	[7-9]
4	GRP78	rs430397	All	3	[10-12]
5	GSTP1	rs1695	All	7	[13-19]
6	HFE	rs1800562	All	14	[20-33]
7	HLA-DP	rs3077	All	4	[34-37]
8	HLA-DP	rs9277535	All	6	[34-38]
9	HLA-DQ	rs2856718	All	4	[34, 35, 39, 40]
10	HULC	rs7763881	All	3	[41-43]
11	IL1B	rs1143627	Asian	7	[44-50]
12	IL-6	rs1800795	All	5	[51-55]
13	IL8	rs2227306	Asian	3	[56-58]
14	IL-28B	rs12979860	All	18	[59-74]
15	MDM2	rs2279744	All	12	[11, 75-85]
16	miR-34b/C	rs4938723	Asian	6	[86-91]
17	MnSOD	rs4880	Caucasian	3	[92-94]
18	NQO1	rs1800566	All	3	[95-97]
19	TGFβ1	rs1800469	All	9	[53, 98-105]
20	TNFα	rs1800629	All	18	[53, 105-120]
21	TNFα	rs1800630	All	10	[47, 99, 108, 111, 115, 116, 121-124]
22	TNFα	rs361525	All	8	[106, 108, 110, 113-115, 119, 122]
23	TLR2	rs3804099	All	4	[125-127]
24	XPD	rs1799793	Asian	4	[128-131]
25	XPD	rs13181	All	8	[48, 128-130, 132-135]
26	XRCC1	rs139599857	All	3	[136-138]
27	XRCC1	rs1799782	All	8	[128, 130, 131, 133, 139-142]
28	GSTT1	Present/Null	All	20	[16, 19, 48, 143-159]

29	UGT1A7	High/Low	All	7	[50, 160-165]
7 genes in Table 4					
1	EGF61	rs4444903	All	16	[166-177]
2	EPHX	rs1051740	All	11	[150, 178-183]
3	HFE	rs1799945	All	16	[20-29, 31-33, 184-186]
4	IL-1B	rs16944	All	5	[47, 48, 187-189]
5	IL-28B	rs8099917	All	13	[59, 61, 63, 67, 69-71, 190-195]
6	miR-196a2	rs11614913	All	17	[196-210]
7	XRCC1	rs25487	All	25	[48, 130, 132, 133, 135, 139, 140, 146, 150, 183, 211-221]

4.Supplemental Table 3. Detailed information for genetic variants significantly associated with HCCr risk in meta-analyses for all available data.

NO	Gene	Variant	Alleles[a]	Ethnicity	MAF(%)	Studies	Cases	Controls	Total	Liver-cancer risk		Heterogeneity		Venice criteria grade[b]	Cumulative evidence of association[c]	FRPR	Amount of evidence		Replication	Protection from Bias	Publication bias		Sensitivity analyses		0.87<OR<1.15
										OR(95%CI)	*P* value	*P* value	I^2				Numbers	Grade			Begg's test	Egger's test	Initial study influence	Deviation from HWE	
1	COL18A1	rs7499	T vs C	Asian	47.23	3	808	1067	1875	0.73(0.64-0.83)	2.32×10^{-6}	0.784	0.00%	AAA	+++	<0.001	1,771	A	A	A	0.296	0.221	NO	None	NO
2	CCND1	rs9344	G vs A	All	48.13	5	805	1255	2060	0.69(0.50-0.94)	0.02	0.001	79.90%	ACA	+	0.223	1,983	A	C	A	0.462	0.484	NO	NO	NO
3	ESR1	rs2234693	C vs T	All	43.16	3	367	458	825	1.51(1.22-1.86)	1.40×10^{-4}	0.145	48.30%	BBA	++	0.003	712	B	B	A	1.000	0.764	NO	None	NO
4	GRP78	rs430397	A vs G	All	15.70	3	1011	1046	2057	1.42(1.20-1.69)	5.66×10^{-5}	0.552	0.00%	BAA	++	0.001	646	B	A	A	0.296	0.063	NO	None	NO
5	GSTP1	rs1695(313A>G)	G vs A	All	28.36	7	1144	1444	2588	1.45(1.08-1.95)	0.014	0.002	73.80%	ACA	+	0.176	1468	A	C	A	0.060	0.197	NO	NO	NO
6	HFE	rs1800562(Cys282Tyr	A vs G	All	5.13	14	1221	3334	4555	1.28(1.02-1.61)	0.033	0.02	49.00%	BBA	++	0.241	468	B	B	A	0.584	0.114	NO	NO	NO
7	HLA-DP	rs3077	A vs G	All	35.51	4	1865	3113	4978	0.77(0.70-0.84)	3.38×10^{-8}	0.172	40.00%	ABA	++	<0.001	3,536	A	B	A	0.734	0.735	NO	None	NO
8	HLA-DP	rs9277535	A vs G	All	41.25	6	2238	4067	6305	0.79(0.64-0.99)	0.036	0.000	81.30%	ACA	+	0.257	5,202	A	C	A	1.000	0.744	NO	None	NO
9	HLA-DQ	rs2856718	G vs A	All	43.24	4	1974	2831	4805	0.67(0.61-0.73)	1.67×10^{-20}	0.126	47.60%	ABA	++	NA	4156	A	B	A	0.734	0.649	NO	NO	NO
10	HULC	rs7763881	C vs A	All	47.56	3	1887	2222	4109	0.90(0.82-0.98)	0.015	0.278	21.90%	AAC	+	0.118	3,908	A	A	C	0.296	0.012	NO	NO	YES
11	IL1B	rs1143627(-31T>C)	C vs T	Asian	46.11	7	1331	1409	2740	0.83(0.74-0.92)	0.001	0.23	26.00%	AAA	+++	0.007	2528	A	A	A	0.368	0.186	NO	None	NO
12	IL-6	rs1800795	C vs G	All	49.16	5	346	911	1257	0.63(0.42-0.95)	0.025	0.109	54.90%	ACC	+	0.373	1,236	A	C	C	1.000	0.141	NO	YES	NO
13	IL8	rs2227306(+781C>T)	T vs C	Asian	36.40	3	486	698	1184	0.78(0.66-0.94)	0.006	0.176	42.40%	BBC	+	0.055	862	B	B	C	1.000	0.779	YES	NO	NO
14	IL-28B	rs12979860	T vs C	All	33.85	17	4267	4862	9129	1.30(1.08-1.58)	0.007	0	70.30%	ACC	+	0.075	6,180	A	C	C	0.021	0.007	NO	NA	NO
15	MDM2	rs2279744	G vs T	All	47.88	12	3011	3219	6230	1.39(1.22-1.60)	1.33×10^{-6}	0.001	63.80%	ACA	+	<0.001	5,966	A	C	A	0.451	0.066	NO	NO	NO
16	miR-34b/C	rs4938723	C vs T	Asian	33.00	6	3134	3624	6758	1.23(1.06-1.44)	0.008	0.001	74.70%	ACC	+	0.067	4,460	A	C	C	0.346	0.046	NO	NO	NO
17	MnSOD	rs4880(Val-9Ala)	C vs T	Caucasian	48.58	3	238	360	598	1.82(1.43-2.32)	1.45×10^{-6}	0.474	0.00%	BAA	++	<0.001	581	B	A	A	1.000	0.868	NO	NO	NO
18	NQO1	rs1800566	T vs C	All	40.70	3	740	765	1505	1.25(1.08-1.45)	0.003	0.676	0.00%	AAA	+++	0.028	1,226	A	A	A	0.296	0.121	NO	None	NO
19	TGFβ1	rs1800469	T vs C	All	46.42	9	2160	3255	5415	1.24(1.02-1.50)	0.030	0.000	79.40%	ACC	+	0.218	5,028	A	C	C	0.251	0.085	YES	YES	NO
20	TNFα	rs1800629	A vs G	All	11.63	18	2125	3663	5788	1.58(1.18-2.12)	0.002	0.000	74.50%	ACA	+	0.050	1,346	A	C	A	0.544	0.777	NO	NO	NO
21	TNFα	rs1800630(-863C>A)	A vs C	All	18.78	10	1994	2957	4951	1.34(1.12-1.61)	0.002	0.022	55.20%	ACA	+	0.017	1860	A	C	A	0.175	0.097	NO	NO	NO
22	TNFα	rs361525	A vs G	All	12.02	8	763	1175	1938	1.49(1.16-1.91)	0.002	0.128	37.80%	BBA	++	0.030	466	B	B	A	0.386	0.324	NO	NA	NO
23	TLR2	rs3804099	C vs T	All	40.29	4	1213	2599	3812	1.33(1.05-1.69)	0.019	0.004	77.90%	ACC	+	0.172	3,072	A	C	C	1.000	0.845	YES	NO	NO
24	XPD	rs1799793(Asp312Asn	T vs C	Asian	22.93	4	1500	1650	3150	1.20(1.06-1.36)	0.006	0.71	0.00%	AAC	+	0.045	1444	A	A	C	1.000	0.643	YES	YES	NO
25	XPD	rs13181(Lys751Gln)	A vs T	All	27.61	8	3457	3940	7397	1.36(1.01-1.83)	0.042	0	90.60%	ACC	+	0.336	4084	A	C	C	0.174	0.006	YES	YES	NO
26	XRCC1	rs139599857	C vs G	All	35.04	3	761	733	1494	2.61(1.20-5.69)	0.016	0.000	89.30%	ACA	+	0.633	1,047	A	C	A	0.296	0.204	NO	NO	NO
27	XRCC1	rs1799782	G vs C	All	24.24	8	1259	1385	2644	1.14(1.00-1.31)	0.047	0.101	43.40%	ABC	+	0.002	1,282	A	B	C	0.548	0.649	NO	YES	YES
Additional variants after stratification by ethnicity																									
1	GSTT1	Present/Null	Null vs present	Caucasian	24.12	7	1030	2059	3089	1.56(1.07-2.28)	0.022	0.000	76.60%	BCA	+	0.317	745	B	C	A	1.000	0.615	NO	NA	NO
2	UGT1A7	High/Low	Low vs High	Asian	32.62	4	294	425	719	2.11(1.54-2.90)	4.16×10^{-6}	0.283	20.90%	BAA	++	0.002	234	B	A	A	1.000	0.519	NO	NA	NO
3	XRCC1	rs25487	G vs A	Asian	26.91	11	4029	4227	8256	1.44 (1.19-1.75)	1.70×10^{-4}	0.000	82.50%	ACC	+	0.002	4,444	A	C	C	0.043	0.340	NO	NO	NO

MAF=minor allele frequency, OR=odds ratio, CI= confidence interval, C=cytosine, G=guanine, A=adenine, T=thymine, FPRP= false positive report probability.

NO: The results were not changed

YES:The results were changed

None: None of studies deviated from HWE

NA:Part of the study could not calculate HWE.

[a]Minor allele versus major allele (reference).

[b]Venice criteria grades are for amount of evidence, replication of the association, and protection from bias.

[c]Cumulative epidemiological evidence was graded as strong (+++), moderate (++), or weak (+) for association with liver cancer risk by Venice criteria.

5.Supplemental Table 4. Detailed information for additional genetic variants significantly associated with HCC risk in meta-analyses using other models.

NO	Variant		Alleles[a]	Ethnicity	MAF(%)	Studies	Cases	Controls	Total	Allelic contrasts			Best genetic model				Venice criteria grade[b]	Cumulative evidence of association[c]	FPRP	Amount of evidence		Replication	Protection from Bias	Publication bias		Sensitivity analyses		0.87<OR<1.15
										OR(95%CI)	*P* value	I^2	Model	OR(95%CI)	p value	I^2				Numbers	Grade			Begg's test	Egger's test	Initial study influence	Deviation from HWE	
1	EGF61	rs4444903	A vs G	All	46.52	16	1943	3505	5448	1.26(0.99-1.60)	0.052	83.50%	DOM	1.32(1.02-1.70)	0.038	66.20%	ACC	+	0.275	3596	A	C	C	0.322	0.182	YES	None	NO
2	EPHX	rs1051740	C vs T	All	31.21	11	816	1780	2596	1.21(0.87-1.67)	0.253	71.00%	REC	1.48(1.09-2.01)	0.012	24.90%	BAC	+	0.162	240	B	A	C	0.152	0.377	YES	YES	NO
3	HFE	rs1799945	G vs C	All	15.21	16	1282	2468	3750	1.15(0.91-1.45)	0.247	53.40%	DOM	1.22(1.05-1.43)	0.012	44.10%	ABB	++	0.098	1061	A	B	B	0.753	0.383	NO	NA	NO
4	IL-1B	rs16944	T vs C	All	47.82	5	949	974	1923	0.89(0.66-1.21)	0.468	73.30%	DOM	0.78(0.63-0.97)	0.024	42.30%	ABC	+	0.190	1404	A	B	C	0.462	0.417	YES	NO	NO
5	IL-28B	rs8099917	G vs T	All	17.99	13	3864	4156	8020	1.08(0.95-1.23)	0.245	36.10%	DOM	1.33(1.06-1.68)	0.014	58.70%	ACB	+	0.131	2146	A	C	B	0.428	0.108	NO	NA	NO
6	miR-196a2	rs11614913	C vs T	All	48.34	17	4992	5910	10902	1.08(0.97-1.21)	0.147	70.60%	DOM	1.18(1.00-1.39)	0.047	63.10%	ACC	+	0.295	8036	A	C	C	0.902	0.531	NO	YES	NO
7	XRCC1	rs25487	G vs A	All	28.85	25	5189	6362	11605	1.18(0.99-1.41)	0.062	83.90%	REC	1.31(1.01-1.71)	0.044	68.10%	ACC	+	0.322	1113	A	C	C	1.000	0.313	NO	YES	NO

MAF=minor allele frequency, OR=odds ratio, CI= confidence interval, C=cytosine, G=guanine, A=adenine, T=thymine, FPRP= false positive report probability.

NO: The results were not changed

YES:The results were changed

None: None of studies deviated from HWE

NA:Part of the study could not calculate HWE.

[a]Minor allele versus major allele (reference).

[b]Venice criteria grades are for amount of evidence, replication of the association, and protection from bias.

[c]Cumulative epidemiological evidence was graded as strong (+++), moderate (++), or weak (+) for association with liver cancer risk by Venice criteria.

6.Supplemental Table 5. Meta-analyses of associations between 25 genetic variants and HCC risk by ethnicity.

NO	Gene	Variant	Comparison[a]	Ethnicity	Frequency(%)[b]	Meta-analyses of the whole population						Meta-analyses in Asians							Meta-analyses in Caucasians						
						Studies	Cases	Controls	OR(95%CI)	*P* value	I^2	Studies	Cases	Controls	Frequency(%)	OR(95%CI)	*P* value	I^2	Studies	Cases	Controls	Frequency(%)	OR(95%CI)	*P* value	I^2
1	COX-2	rs689466	A vs G	All	46.14	5	1537	1547	1.15(0.95-1.39)	0.166	62.70%	3	1288	1290	49.46	1.12(0.90-1.40)	0.308	62.70%							
2	CYP1A1	rs4646903(MspI site/T6235C)	C vs T	All	40.9	4	695	871	1.17(0.79-1.74)	0.433	76.30%	3	655	831	46.20	1.05(0.81-1.38）	0.703	61.30%							
3	EGF61	rs4444903	A vs G	All	46.52	16	1943	3505	1.26(0.99-1.59)	0.052	83.50%	9	1759	2199	46.6	0.99(0.90-1.11)	0.966	9%	4	153	275	47.78	1.31(0.62-2.77)	0.485	82.70%
4	HOGG1	rs1052133(Ser326Cys)	G vs C	All	48.07	7	1069	1703	1.15(0.87-1.52)	0.336	81.50%	4	899	1341	49.84	1.24(0.89-1.71)	0.207	85.30%	3	170	362	40.60	0.98(0.51-1.91)	0.958	80.40%
5	IL-1B	rs16944	T vs C	All	47.82	5	949	974	0.89(0.66-1.21)	0.468	73.30%	4	890	821	45.06	0.82(0.59-1.12)	0.210	73.90%							
6	IL-6	rs1800796	G vs C	All	47.13	7	1664	1913	1.02(0.82-1.26)	0.893	55.10%	3	1373	1444	38.29	0.94(0.76-1.17)	0.567	56.20%	4	291	469	79.93	1.15(0.73-1.81)	0.539	58.70%
7	IL-8	rs4073(−251T>A)	A vs T	All	41.74	5	1052	1249	1.07(0.86-1.32)	0.545	64.10%	4	940	1144	41.17	1.05(0.81-1.35)	0.728	72.80%							
8	IL10	rs1800872(-592A>C)	C vs A	All	44.91	9	2609	3405	1.04(0.84-1.28)	0.722	81.90%	8	2549	3405	45.40	1.13(0.94-1.35)	0.188	75.10%							
9	MALAT1	rs619586	G vs A	All	8.24	5	2872	4023	0.95(0.84-1.08)	0.449	24.80%	4	2802	3953	8.00	0.97(0.85-1.09)	0.585	33.20%							
10	MTHFR	rs1801131(A1298C)	C vs A	All	20.59	8	1447	2664	0.96(0.75-1.23)	0.732	77.10%	5	1137	1971	18.40	0.85(0.64-1.13)	0.263	75.60%							
11	MTHFR	rs1801133	T vs C	All	41.88	17	4973	6693	1.01(0.89-1.15)	0.887	78.40%	10	4336	5540	42.89	0.98(0.85-1.12)	0.715	78.30%	4	329	460	39.61	1.40(0.69-2.81)	0.351	89.10%
12	miR-146a	rs2910164	C vs G	All	44.61	21	5728	7021	1.01(0.91-1.11)	0.919	65.90%	18	5224	6329	41.15	1.01(0.91-1.12)	0.910	70.70%	3	504	692	21.91	0.99(0.82-1.21)	0.722	0.00%
13	miR-196a2	rs11614913	C vs T	All	48.34	17	4992	5910	1.08(0.97-1.21)	0.147	70.60%	12	4522	5330	47.28	1.11(0.98-1.25)	0.102	74.90%	5	470	580	58.24	0.98(0.73-1.34)	0.916	61.10%
14	TNFα	rs1799724	T vs C	All	16.73	7	1334	2115	0.98(0.85-1.13)	0.783	0.00%	6	1320	2030	16.85	0.98(0.85-1.13)	0.763	0.00%							
15	XRCC1	rs25487(Arg399Gln)	G vs A	All	28.85	25	5189	6362	1.18(0.99-1.41)	0.062	83.90%	13	4332	4922	26.91	1.44(1.19-1.75)	1.70×10^{-4}	82.50%	12	806	1440	35.96	1.18(0.99-1.41)	0.648	72.10%
16	XRCC1	rs25489(Arg280His)	A vs G	All	16.69	7	913	1063	1.20(0.85-1.68)	0.308	68.90%								4	265	367	28.01	1.08(0.58-2.02)	0.816	81.10%
17	NAT2	Slow vs rapid	Slow vs rapid	All	43.28	7	1055	1302	0.99(0.81-1.20)	0.89	22.30%	3	445	561	22.27	1.23(0.87-1.70)	0.253	19.70%	4	610	741	59.07	0.88(0.69-1.13)	0.31	4.10%
18	CYP2E1	rs2031920	T vs C	All	14.11	11	1297	2355	0.99(0.71-1.39)	0.952	59.20%	7	1011	1497	20.26	0.94(0.79-1.11)	0.46	40.60%	4	268	858	2.71	1.21(0.26-5.65)	0.805	72.60%
19	MIR499	rs3746444	G vs A	All	23.90	15	2816	3771	1.14(0.94-1.37)	0.189	79.20%	11	2531	3376	21.45	1.20(0.97-1.47)	0.088	79.70%	4	285	395	45.15	0.96(0.58-1.61)	0.879	79.50%
20	MIR149	rs2292832	C vs T	All	45.07	7	1429	2068	0.95(0.86-1.06)	0.357	45.40%	6	1309	1968	45.24	0.94(0.84-1.04)	0.241	49.30%							
21	IL18	rs187238(-137G>C)	C vs G	All	15.09	6	740	1808	0.99(0.62-1.57)	0.963	82.20%	4	598	1325	11.23	0.65(0.25-1.72)	0.385	90.50%							
22	IL18	rs1946518(-607A>C)	C vs A	All	49.88	6	764	1319	0.90(0.69-1.18)	0.446	69.50%	3	542	767	49.31	1.05(0.90-1.23)	0.526	0.00%							
23	GSTM1	Present/Null	null vs present	All	49.80	24	4209	6971	1.13(0.92-1.39)	0.238	82.90%	13	2802	4205	53.90	1.04(0.80-1.36)	0.749	82.50%	8	1086	2148	45.36	1.28(0.80-2.04)	0.305	88.50%
24	GSTT1	Present/Null	null vs present	All	37.78	20	3801	6060	1.21(0.99-1.46)	0.052	74.40%	10	2459	3383	45.17	1.06(0.83-1.35)	0.643	74.90%	7	1030	2059	24.12	1.56(1.07-2.28)	0.022	76.60%
25	UGT1A7	High/Low	Low vs High	All	54.08	7	521	936	1.48(0.72-3.07)	0.287	88.10%	4	294	425	32.62	2.11(1.54-2.90)	4.16×10^{-6}	20.90%	3	227	513	75.87	1.11(0.34-3.57)	0.865	88.80%

OR=odds ratio, CI= confidence interval, C=cytosine, G=guanine, A=adenine, T=thymine.

[a]Allelic contrast or phenotype for common variants, or genetic comparison with only genotype group data.

[b]Frequency of minor allele or effect genotype.

7.Supplemental Table 6. Previously published meta-analyses compared to current study in our study

			Prior meta-analyses															Current study								
NO	Genes	Variants	PubMed ID	DOI	Reference	First author	Year	Retrieved databases	Close date	Ethinicity	Studies	Cases	Controls	Comparison	OR(95%CI)	*P* value	P_h	Ethinicity	Studies	Cases	Controls	Comparison	OR(95%CI)	*P* value	P_h	Comments for prior meta-analyses
1	ALDH2	rs671	30921184	10.1097/MD.000000000001485	[1]	Zuo	2019	PubMed and Embase	March-18	Asian	7	1522	4493	A vs G	0.96(0.83-1.11)	NA	0.155	Asian	10	2153	5051	A vs G	1.06(0.90-1.24)	0.494	0.009	
			22231434	10.1007/s13277-011-0309-8	[2]	Zhou	2012	PubMed and Embase	November-11	Asian	10	1231	1849	AG+GG vs GG	0.91(0.70-1.18)	NA	0.021	Asian	10	2153	5051	A vs G	1.06(0.90-1.24)	0.494	0.009	
			31987047	10.1186/s12957-020-1796-0	[3]	Chen	2020	MEDLINE, PubMed, Scopus, Embase, and China Academic Journals databases	July-19	Asian	8	1966	4604	AA vs GG	1.08(0.87-1.34)	0.509	0.133	Asian	10	2153	5051	AA vs GG	1.10(0.89-1.36)	0.391	0.233	
2	BIRC5	rs9904341	24098667	10.1371/journal.pone.0074778	[4]	Zhu	2013	PubMed, EMBASE, and CNKI	January-13	All	3	473	933	C vs G	0.83(0.71-0.98)	0.032	0.886	All	4	533	963	C vs G	0.85(0.73-1.00)	0.052	0.715	
3	CD95	rs1800682	25723590	10.12659/MSM.892547	[5]	Zhu	2015	PubMed and SinoMed	June-14	Asian	3	456	604	AA+AG vs GG	0.93(0.87-0.99)	0.521	0.035	Asian	3	456	604	GG vs GA+AA	1.39(1.03-1.88)	0.031	0.463	
4	COX-2	rs20417	25730260	10.1371/journal.pone.0118251	[6]	Chen	2015	PubMed, EMBASE, Google Scholar,CNKI and WanFang database	April-14	All	5	1117	1997	C vs G	1.22(0.70-2.14)	0.480	0.000	All	4	847	857	C vs G	0.99(0.55-1.79)	0.981	0.000	Included 1 studies published in Chinese
			26438136	10.1136/bmjopen-2015-008263	[7]	Lu	2015	EMBASE, PubMed, Public Library of Science,SCOPUS, Web of Knowledge, CNKI	March-15	All	6	1295	1563	C vs G	1.32(0.76-2.30)	0.330	<0.001	All	4	847	857	C vs G	0.99(0.55-1.79)	0.981	0.000	Included 2 studies published in Chinese
5	COX-2	rs689466	26438136	10.1136/bmjopen-2015-008263	[7]	Lu	2016	EMBASE, PubMed, Public Library of Science,SCOPUS, Web of Knowledge, CNKI	March-15	All	8	2060	2618	GG vs GA+AA	0.72(0.57-0.92)	0.008	0.040	All	5	1537	1547	A vs G	1.15(0.95-1.39)	0.166	0.030	Included 3 studies published in Chinese
6	CTLA4	rs231775	30539855	10.4103/0973-1482.203604	[8]	Wang	2018	PubMed and China Biological Medicine Database	June-16	Asian	4	1449	1504	G vs A	0.72(0.57-0.92)	0.007	0.020	Asian	5	1971	2464	A vs G	0.99(0.76-1.30)	0.982	0.000	
7	DNMT3B	rs2424913	21938431	10.1007/s11033-011-1231-2	[9]	Zhu	2012	MEDLINE, ISI Web of Knowledge and Embase	July-11	All	2	196	362	C vs T	1.08(0.77-1.52)	NA	0.230	All	3	304	578	T vs C	0.96(0.69-1.34)	0.813	0.273	
8	EGF61	rs4444903		NA	[10]	Zhang	2016	PubMed and Wan Fang	July-14	All	9	1874	2302	G vs A	0.93(0.88-0.98)	0.010	0.118	All	16	1943	3505	A vs G	1.26(0.99-1.60)	0.052	0.052	Included 2 studies published in Chinese
			25927412	10.1186/s12885-015-1318-6	[11]	Jiang	2015	PubMed, Embase, Cochrane Library, Web of Science, CBM, Wanfang and CNKI	December-13	All	16	2475	5381	G vs A	1.38(1.17-1.63)	<0.001	0.754	All	16	1943	3505	A vs G	1.26(0.99-1.60)	0.052	0.052	Included 1 studies published in Chinese
8	EPHX	rs1051740	23955801	10.1007/s13277-013-1090-7	[12]	Duan	2013	PubMed, EMBASE, ISI Web of Science, Wanfang database, and CNKI	NA	All	12	1333	3000	CC vs TC+TT	0.93(0.81-1.07)	0.330	0.370	All	11	816	1780	CC vs TC+TT	1.48(1.09-2.01)	0.012	0.214	Included 3 studies published in Chinese
9	ESR1	rs2234693	25778331	NA	[13]	Sun	2015	PubMed	April-14	All	2	236	351	C vs T	1.36(1.06-1.74)	NA	0.222	All	3	367	458	C vs T	1.51(1.22-1.86)	0.000	0.145	
10	ESR1	rs9340799	25778331	NA	[13]	Sun	2015	PubMed	April-14	All	2	236	349	G vs A	1.28(0.95-1.71)	NA	0.748	All	3	367	458	G vs A	1.24(0.97-1.58)	0.083	0.896	
11	GSTP1	rs1695	23765758	10.1007/s13277-013-0695-1	[14]	Zhao	2013	PubMed, Chinese Biomedical Literature, and Wanfang databases	June-12	All	6	766	1074	GG+GA vs AA	0.91(0.68-1.20)	0.498	0.089	All	7	1143	1444	G vs A	1.45(1.08-1.95)	0.014	0.002	
12	HFE	rs1799945	27657935	10.1371/journal.pone.0163423	[15]	Ye	2016	PubMed, EMBASE, Web of Science , CNKI	August-16	All	15	1745	3176	GC+GG vs CC	1.13(0.92-1.38)	0.238	0.023	All	16	1282	2468	GC+GG vs CC	1.22(1.05-1.43)	0.012	0.030	Included 1 studies published in Chinese
13		rs1800562	27657935	10.1371/journal.pone.0163423	[15]	Ye	2016	PubMed, EMBASE, Web of Science, Scopus and CNKI	August-16	All	15	NA	NA	A vs G	1.55(1.12-2.14)	0.008	0.001	All	14	1221	3334	A vs G	1.28(1.02-1.61)	0.033	0.020	Included 1 studies published in Chinese
14	HLA-DP	rs3077	25365208	10.1371/journal.pone.0111677	[16]	Liao	2014	Pubmed, Embase	NA	Asian	4	2997	3738	A vs G	0.94(0.87-1.02)	NA	0.467	All	4	1865	3113	A vs G	0.77(0.70-0.84)	0.000	0.172	
15		rs7453920	25365208	10.1371/journal.pone.0111677	[16]	Liao	2014	Pubmed, Embase	NA	All	4	2813	3347	A vs G	0.89(0.76-1.02)	NA	0.280	All	3	1409	1946	GG vs GA+AA	0.96(0.62-1.51)	0.873	0.116	
16		rs9277535	25365208	10.1371/journal.pone.0111677	[16]	Liao	2014	Pubmed, Embase	NA	Asian	5	3386	5621	A vs G	1.04(0.97-1.11)	NA	0.057	All	6	2238	4067	A vs G	0.79(0.64-0.99)	0.036	0.000	
17	HLA-DQ	rs2856718	29416599	10.18632/oncotarget.22941	[17]	Xu	2018	PubMed, EMBASE, CNKI and Chinese WanFang databases	June-17	Asian	4	NA	NA	G vs A	0.80(0.76-0.90)	< 0.01	0.900	All	4	1974	2831	G vs A	0.67(0.62-0.73)	0.000	0.126	
18	HLA-G	rs371194629	27706615	10.4238/gmr.15038263	[18]	Coelho	2016	PubMed database	NA	All	6	1114	1644	Ins vs del	1.05(0.76-1.43)	0.750	0.001	All	5	1051	1397	Ins vs del	1.02(0.76-1.37)	0.913	0.001	Included 1 studies without HCC stata
19	HOGG1	rs1052133	28415770	10.18632/oncotarget.16226	[19]	Kang	2017	Pubmed, Embase, google of scholar, and KISS	April-15	Asian	7	NA	NA	C vs G	1.09(0.88-1.34)	0.424	0.001	All	6	919	1553	GC vs CC	1.32(0.89-1.96)	0.170	0.011	Included 4 studies published in Chinese
			23577090	10.1371/journal.pone.0060178	[20]	Wang	2013	PubMed, EMBASE, ISI, BIOSIS, CNKI and Chinese WanFang databases	August-12	Asian	8	2369	2442	CT+CC vs TT	1.38(1.02-1.85)	< 0.001	0.520	All	5	729	1037	GC vs CC	1.49(1.01-2.20)	0.043	0.081	Included 5 studies published in Chinese
20	IL-1B	rs16944	21107607	10.1007/s00432-010-0959-8	[21]	Yang	2011	PubMed, EMBase, Cochrane Central Register of Controlled Trials and ISI Web of Sci	September-10	Asian	5	848	956	C vs T	1.12(0.66-1.88)	0.680	NA	All	5	949	974	CT+TT vs CC	0.78(0.63-0.97)	0.024	0.140	
21	IL-6	rs1800795	33327352	10.1097/md.0000000000023659	[22]	An	2020	PubMed, Cochrane Database, EMBASE, Web of science, and CNKI	May-20	Caucasian	3	1381	523	G vs C	1.49(0.95-2.32)	0.080	0.040	All	5	436	843	C vs G	0.63(0.43-0.95)	0.025	0.109	
			26096712	10.1038/srep11394	[23]	Tian	2015	PubMed and Embase	August-14	All	5	539	850	C vs G	0.74(0.61-0.89)	0.001	0.000	All	5	436	843	C vs G	0.63(0.43-0.95)	0.025	0.109	
22		rs1800796	33511285	10.5114/ceh.2020.102171	[24]	Aleagh	2021	Scopus, PubMed, Web of Science, and Cochrane Library	June-20	All	7	NA	NA	G vs C	0.93(0.83- 1.05)	0.250	0.150	All	7	1664	1913	G vs C	1.02(0.82-1.26)	0.893	0.038	
23	IL10	rs1800872	28977953	10.18632/oncotarget.18220	[25]	Wang	2017	PubMed, Embase,CNKI and Wanfang database	NA	Asian	5	1859	2109	C vs A	1.08(0.93-1.24)	0.094	NA	Asian	9	2609	3405	A vs C	0.87(0.70-1.08)	0.201	0.000	
24	IL-28B	rs12979860	31568008	10.1097/MD.000000000001727	[26]	Qin	2019	PubMed,EMBASE,CNKI	October-18	All	18	4513	10537	CC vs CT/TT	0.71(0.58-0.88)	0.002	0.000	All	17	4267	4862	T vs C	1.30(1.08-1.58)	0.007	0	Included 1 studies published in Chinese
25		rs8099917	31568008	10.1097/MD.000000000001727	[26]	Qin	2019	PubMed,EMBASE,CNKI	October-18	All	15	4212	9701	TT vs TG/GG	0.82(0.72-0.94)	0.005	0.307	All	13	3864	4156	GT+GG vs TT	1.33(1.06-1.68)	0.014	0.004	Included 2 studies published in Chinese
26	MALAT1	rs619586	32720826	10.1177/0300060520941969	[27]	Ni	2020	PubMed, Embase, CNKI	March-20	Asian	4	2802	3953	G vs A	0.96(0.85-1.10)	0.590	0.210	All	5	2872	4023	G vs A	0.95(0.84-1.08)	0.449	0.256	
27	MBOAT7	rs641738	32882372	10.1016/j.jhep.2020.08.027	[28]	Teo	2021	Science direct	July-20	All	4	228	2100	CC vs CT + TT	1.64(1.18-2.27)	0.003	0.950	All	3	1413	1903	T vs C	1.29(0.96-1.68)	0.098	0.018	
28	MDM2	rs2279744	31935498	10.1016/j.gene.2020.144365	[29]	Zhang	2020	PubMed, Web of Science, Embase, CNKI , and Wanfang databases	January-19	Asian	7	2703	2760	G vs C	1.37(1.18-1.60)	NA	0.002	All	12	3011	3219	G vs T	1.39(1.22-1.60)	0.000	0.001	
			28885338	10.1097/MD.000000000000785	[30]	Duan	2017	MEDLINE, Web of science, EMBASE, CochraneLibrary, and CNKI	May-17	All	15	4032	5491	G vs T	1.48(1.26-1.73)	<0.001	<0.001	All	12	3011	3219	G vs T	1.39(1.22-1.59)	0.000	0.001	Included 4 studies published in Chinese
29	MTHFR	rs1801131	30917367	10.1159/000496428	[31]	Su	2019	PubMed, Medline, and Embase	February-18	All	8	1531	2736	C vs A	1.12(1.00-1.26)	0.050	0.770	All	8	1447	2664	C vs A	0.96(0.75-1.23)	0.732	0.000	Included 1 studies published in Chinese
			26662389	10.4238/2015.December.7.9	[32]	Chang	2015	PubMed,Embase	October-14	All	5	1011	1690	CC vs AA	0.52(0.33-0.81)	NA	0.430	All	8	1447	2664	C vs A	0.96(0.75-1.23)	0.732	0.000	
			24014085	10.1007/s13277-013-1046-y	[33]	Liang	2014	PubMed and Wanfang databases	March-13	All	7	2030	3096	C vs A	0.96(0.86-1.06)	0.413	NA	All	8	1447	2664	C vs A	0.96(0.75-1.23)	0.732	0.000	
30		rs1801133	30917367	10.1159/000496428	[31]	Su	2019	PubMed, Medline, and Embase	February-18	All	16	3547	4831	T vs C	1.03(0.97-1.10)	0.320	0.780	All	17	4973	6693	T vs C	1.01(0.89-1.15)	0.887	0.000	Included 2 studies published in Chinese
			24132589	10.1007/s13277-013-1023-5	[34]	Sun	2014	PubMed and Wangfang databases	January-13	All	15	NA	NA	TT vs CC	1.19(1.03-1.37)	0.016	0.043	All	17	4973	6693	T vs C	1.01(0.89-1.15)	0.887	0.000	Included 2 studies published in Chinese
31	miR-146a	rs2910164	32132049	10.21873/anticanres.14094	[35]	Park	2020	PUBMED/MEDLINE and the Cochrane Database/EBM	July-19	All	13	4246	4128	C vs. G	1.10(0.91-1.12)	NA	0.022	All	20	5546	6671	C vs G	1.01(0.92-1.11)	0.847	0.000	
			24860819	10.1155/2014/468605	[36]	Wang	2014	PubMed, EMBASE, Cochrane, ScienceDirect, CNKI , and Wanfang databases	February-14	All	17	5689	6790	C vs G	0.88(0.84-0.93)	0.000	0.230	All	20	5546	6671	C vs G	1.01(0.92-1.11)	0.847	0.000	
			28499985	10.1016/j.clinre.2017.03.005	[37]	Zheng	2017	PubMed, EMBase,CNKI and Wanfang	October-16	All	22	7894	10221	C vs G	1.03(0.95-1.11)	0.480	NA	All	20	5546	6671	C vs G	1.01(0.92-1.11)	0.847	0.000	Included 6 studies published in Chinese
			33542895	10.3389/fonc.2020.562019	[38]	Zhang	2021	PubMed,EMBASE,Cochrane Central Register	May-19	All	17	4956	5853	GC+CC vs GG	1.00(0.90-1.11)	NA	0.090	All	20	5546	6671	C vs G	1.01(0.92-1.11)	0.847	0.000	
32	miR-196a2	rs11614913	29371991	10.18632/oncotarget.22547	[39]	Yan	2017	PubMed, ISI Web of Knowledge, EMBASE, CNKI	December-16	All	10	2232	3195	TT + TC vs. CC	1.07(0.94-1.22)	0.280	<0.001	All	17	4992	5910	CT+CC vs TT	1.18(1.00-1.39)	0.047	0.000	
			33542895	10.3389/fonc.2020.562019	[38]	Zhang	2021	PubMed、EMBASE、Cochrane Central Register	May-19	All	15	4543	4329	TC+CC vs TT	1.23(1.03-1.48)	NA	0.000	All	17	4992	5910	TC+CC vs TT	1.08(0.91-1.29)	0.397	0.000	
			28499985	10.1016/j.clinre.2017.03.005	[37]	Zheng	2017	PubMed, EMBase, CNKI and Wanfang	October-16	All	17	6937	8217	CC + CT vs TT	1.20(1.04-1.37)	0.010	NA	All	17	4992	5910	TC+CC vs TT	1.08(0.91-1.29)	0.397	0.000	
33	miR-34b/C	rs4938723	29976775	10.1042/bsr20180712	[40]	Wang	2018	PubMed and Web of Science	February-18	Asian	4	1958	2321	CC + CT vs TT	1.19(1.03-1.37)	0.016	NA	Asian	6	3134	3624	C vs T	1.23(1.06-1.44)	0.008	0.001	
34	NQO1	rs1800566	24532470	10.1007/s13277-014-1712-8	[41]	Fan	2014	PubMed, Embase, Web of Science, and Wanfang databases	NA	All	3	779	816	T vs C	1.47(1.07-2.00)	0.016	0.029	All	5	1022	1039	T vs C	1.34(1.07-1.67)	0.010	0.039	
			25773831	10.7314/apjcp.2015.16.5.1821	[42]	Zhao	2015	PubMed, EBSCO, CNKI , and the Wanfang databases	May-14	All	5	1325	1367	T vs C	1.45(1.23-1.72)	<0.01	0.080	All	5	1022	1039	T vs C	1.34(1.07-1.67)	0.010	0.039	
35	NAT2	Phenotype	22293947	10.1007/s13277-012-0340-4	[43]	Zhang	2012	PubMed, EMBASE, CNKI, and Wan Fang	December-11	All	8	1084	1682	rapid vs slow	1.03(0.86-1.24)	NA	0.110	All	7	1055	1302	Slow vs rapid	0.99(0.81-1.20)	0.890	0.259	Included 2 studies published in Chinese
36	TGFβ1	rs1800469	31935498	10.1016/j.gene.2020.144365	[44]	Zhang	2020	PubMed, Web of Science, Embase, CNKI , and Wanfang databases	January-19	Asian	4	1657	2266	C vs T	1.00(0.82-1.21)	NA	0.009	All	9	2160	3255	T vs C	1.24(1.02-1.50)	0.030	0.000	
			NA	10.4149/neo_2016_615	[45]	Toshiku	2016	PubMed	January-16	All	8	1427	3735	TT+CT vs. CC	1.44(0.99-2.10)	0.058	<0.0001	All	9	2160	3255	T vs C	1.24(1.02-1.50)	0.030	0.000	
			23653379	10.1007/s13277-013-0818-8	[46]	Li	2013	PubMed, Embase, and Web of Science	March-13	All	11	2577	4107	TT+CT vs CC	1.14(0.87-1.48)	NA	0.000	All	9	2160	3255	T vs C	1.24(1.02-1.50)	0.030	0.000	Included 2 studies published in Chinese
37	TNFα	rs1799724	28115787	10.1155/2016/4580323	[47]	Wang	2016	PubMed, Medline, Embase, Web of Science	November-15	Asian	4	807	1510	T vs C	1.16(0.89-1.51)	0.270	0.080	All	7	1334	2115	T vs C	0.98(0.85-1.13)	0.783	0.874	
38	TNFα	rs1800629	30027452	10.1007/s12029-018-0135-y	[48]	Horesta	2019	PubMed, Science Direct, and Google Scholar Databases	October-16	All	6	925	1307	A vs G	0.98(0.86-1.12)	0.764	NA	All	18	2125	3663	A vs G	1.58(1.18-2.12)	0.002	0.000	
			27977601	10.1097/MD.000000000000560	[49]	Xiao	2016	PubMed, ScienceDirect Online Wiley online library	September-16	All	8	735	1116	A vs G	1.42(1.11-1.81)	0.005	0.108	All	18	2125	3663	A vs G	1.58(1.18-2.12)	0.002	0.000	
39	TNFα	rs361525	30027452	10.1007/s12029-018-0135-y	[48]	Horesta	2019	PubMed, Science Direct, and Google Scholar Databases	October-16	All	5	541	795	A vs G	0.99(0.83-1.19)	0.983	NA	All	8	763	1175	A vs G	1.49(1.160-1.91	0.002	0.128	
			27977601	10.1097/MD.000000000000560	[49]	Xiao	2016	PubMed, ScienceDirect Online Wiley online library	September-16	All	5	578	834	A vs G	1.29(0.93-1.81)	0.130	0.053	All	8	763	1175	A vs G	1.49(1.160-1.91	0.002	0.128	
40	TNFα	rs1800630	33228594	10.1186/s12885-020-07606-6	[50]	Wungu	2020	PubMed, ProQuest, EBSCO, Science Direct, and Springer	January-20	All	7	1612	2492	A vs C	1.31(1.03-1.67)	0.030	0.007	All	10	1994	2957	A vs C	1.34(1.12-1.61)	0.002	0.022	
			21336601	10.1007/s10620-011-1617-y	[51]	Wei	2011	Medline, Embase, CNKI, and Chinese Biomedicine Database	November-10	All	5	627	1004	AA/AC vs CC	1.65(1.06-2.57)	0.026	0.006	All	10	1994	2957	A vs C	1.34(1.12-1.61)	0.002	0.022	Included 1 studies published in Chinese
41	XRCC1	rs1799782	30408066	10.1371/journal.pone.0206853	[52]	Xiong	2018	PubMed, Web of Science, Cochrane Library, CNKI , and the Wanfang database	March-18	All	13	2262	2412	Trp/Trp vs Arg/Trp	1.42(1.24-1.62)	NA	0.010	All	8	1259	1385	G vs C	1.14(1.00-1.31)	0.047	0.101	Included 5 studies published in Chinese
			25202399	10.3892/ol.2014.2351	[53]	Li	2014	PubMed,Google Scholar,and CNKI	October-13	All	6	1451	1398	Trp/Trp vs. Arg/Trp	0.94(0.59-1.51)	NA	0.030	All	8	1259	1385	G vs C	1.14(1.00-1.31)	0.047	0.101	
			23055199	10.1007/s13277-012-0546-5	[54]	Li	2013	PubMed, Embase, CNKI, and Chinese Biomedicine Database	July-12	Asian	5	980	966	GG vs CC	1.07(0.67-1.69)	NA	0.214	All	8	1259	1385	G vs C	1.14(1.00-1.31)	0.047	0.101	Included 4 studies published in Chinese
42		rs25487	30408066	10.1371/journal.pone.0206853	[52]	Xiong	2018	PubMed, Web of Science, Cochrane Library, CNKI, and the Wanfang databases	March-18	All	25	5154	6544	GG vs AG+AA	1.40(1.23-1.59)	NA	0.000	All	25	5189	6362	GG vs AG+AA	1.31(1.01-1.71)	0.044	0.000	Included 9 studies published in Chinese
			24815482	10.7314/apjcp.2014.15.7.3273	[55]	Zhang	2014	PubMed, Embase, Cochrane Library, VIP and CNKI	November-13	All	21	4170	5030	GG vs AG+AA	1.24(1.02-1.51)	0.030	0.000	All	25	5189	6362	GG vs AG+AA	1.31(1.01-1.71)	0.044	0.000	Included 8 studies published in Chinese
			24390232	10.1007/s11033-013-2929-0	[56]	Qi	2014	PubMed, Embase, Chinese biomedicine databases, CNKI, and the Wanfang databases	August-12	All	15	2554	3321	GG vs AG+AA	1.05(0.91-1.21)	NA	0.370	All	25	5189	6362	GG vs AG+AA	1.31(1.01-1.71)	0.044	0.000	
43		rs25489	26125922	10.4238/2015.June.29.5	[57]	Xu	2015	NCBI Global Cross-database	NA	All	10	1848	1969	His/His vs Arg/Arg	1.55(1.10-2.18)	0.013	0.517	All	7	913	1063	A vs G	1.19(0.85-1.68)	0.308	0.007	Included 5 studies published in Chinese
			30408066	10.1371/journal.pone.0206853	[52]	Xiong	2018	PubMed, Web of Science, Cochrane Library, CNKI , and the Wanfang database	March-18	All	13	2007	2105	His/His vs Arg/Arg	1.43(0.91-2.25)	NA	0.150	All	7	913	1063	A vs G	1.19(0.85-1.68)	0.308	0.007	Included 7 studies published in Chinese
			23679314	10.7314/apjcp.2013.14.3.2033	[58]	Li	2013	PubMed, EMBASE and CNKI	July-05	Asian	7	1448	1544	A vs G	1.46(0.99-2.13)	NA	0.820	All	7	913	1063	A vs G	1.19(0.85-1.68)	0.308	0.007	Included 3 studies published in Chinese
44	XRCC3	rs861539	27306318	10.1111/jcmm.12896	[59]	Shi	2016	PubMed and Embase	October-15	NA	6	NA	NA	Met vs Thr	1.93(1.17-3.20)	0.011	0.961	All	5	2624	3532	T vs C	1.34(0.83-2.14)	0.229	0.000	
			23873110	10.1007/s13277-013-0973-y	[60]	Wu	2013	PubMed, Embase and Wanfang databases	NA	Asian	7	2288	3249	T vs C	1.09(0.92-1.30)	0.316	0.379	All	5	2624	3532	T vs C	1.34(0.83-2.14)	0.229	0.000	Included 3 studies published in Chinese
45	XPD	rs1799793	33517857	10.1177/1533033821990046	[61]	Zhou	2021	Pubmed, Web of Science and China Academic Journals Full-text Database	NA	All	6	2225	2442	T vs C	1.23(1.04-1.46)	0.017	0.082	Asian	4	1500	1650	T vs C	1.20(1.06-1.36)	0.006	0.710	Included 2 studies published in Chinese
			27306318	10.1111/jcmm.12896	[59]	Shi	2016	PubMed and Embase	October-15	NA	4	NA	NA	T vs C	1.22(1.08-1.38)	0.001	0.000	Asian	4	1500	1650	T vs C	1.20(1.06-1.36)	0.006	0.710	
46		rs13181	33517857	10.1177/1533033821990046	[61]	Zhou	2021	Pubmed, Web of Science and China Academic Journals Full-text Database	NA	All	13	4458	5025	G vs T	1.36(1.03-1.80)	0.028	0.000	All	8	3457	3940	A vs C	1.36(1.01-1.83)	0.042	0.000	Included 5 studies published in Chinese
			27306318	10.1111/jcmm.12896	[59]	Shi	2016	PubMed and Embase	October-15	NA	6	NA	NA	A vs C	1.42(1.02-1.97)	0.038	0.927	All	8	3457	3940	A vs C	1.36(1.01-1.83)	0.042	0.000	
47	CYP2E1	rs2031920	27637154	10.1016/j.dld.2016.08.114	[62]	Fu	2016	PubMed, EMBASE, Cochrane, Web of Science, Sinomed, CNKI, Wanfang database.	NA	Mixed	16	1737	2947	CT+TT vs CC	0.99 (0.85-1.14)	0.860	0.079	All	11	1297	2355	T vs C	0.99(0.71-1.39)	0.952	0.012	Included 6 studies published in Chinese
			31987047	10.1186/s12957-020-1796-0	[63]	Chen	2020	MEDLINE, PubMed, Scopus, Embase, and China Academic Journals databases	July-19	Asian	12	1418	1701	CT+TT vs CC	0.82(0.53-1.26)	0.358	0.000	All	11	1297	2355	T vs C	0.99(0.71-1.39)	0.952	0.012	Included 5 studies published in Chinese
48	MIR499	rs3746444	31935498	10.1016/j.gene.2020.144365	[64]	Zhang	2020	PubMed, Web of Science, Embase, CNKI, and Wanfang databases	January-19	Asian	4	874	1317	C vs T	1.36(1.05-1.77)	NA	0.167	All	15	2816	3771	G vs A	1.14(0.94-1.37)	0.189	0.000	
49	MIR149	rs2292832	31935498	10.1016/j.gene.2020.144365	[64]	Zhang	2020	PubMed, Web of Science, Embase, CNKI, and Wanfang databases	January-19	Asian	4	823	1365	C vs T	1.07(0.86-1.33)	NA	0.043	All	7	1429	2068	C vs T	0.95(0.86-1.06)	0.357	0.089	
50	IL18	rs187238	28000712	10.1038/srep39404	[65]	Zhu	2016	PubMed, EMBASE, Google Scholar and CNKI	September-16	All	8	1318	2254	C vs G	0.99(0.74-1.34)	0.970	<0.001	All	6	740	1808	C vs G	0.99(0.62-1.57)	0.963	0.000	Included 2 studies published in Chinese
			31935498	10.1016/j.gene.2020.144365	[64]	Zhang	2020	PubMed, Web of Science, Embase, CNKI, and Wanfang databases	January-19	Asian	6	1150	1494	G vs C	1.09(0.74-1.56)	NA	<0.001	All	6	740	1808	C vs G	0.99(0.62-1.57)	0.963	0.000	Included 2 studies published in Chinese
51	IL18	rs1946518	28000712	10.1038/srep39404	[65]	Zhu	2016	PubMed, EMBASE, Google Scholar and CNKI	September-16	All	7	1262	1696	C vs A	0.99(0.81-1.22)	0.940	0.002	All	6	764	1319	C vs A	0.90(0.69-1.18)	0.446	0.006	Included 2 studies published in Chinese
			31935498	10.1016/j.gene.2020.144365	[64]	Zhang	2020	PubMed, Web of Science, Embase, CNKI, and Wanfang databases	January-19	Asian	6	1150	1494	C vs A	1.06 (0.88-1.29)	NA	0.019	All	6	764	1319	C vs A	0.90(0.69-1.18)	0.446	0.006	Included 2 studies published in Chinese
52	CCND1	rs9344	27499641	10.2147/OTT.S108754	[66]	Luo	2016	PubMed, EMBASE, Cochrane, Web of Science, CBL, CNKI and Google Scholar	March-16	All	6	1030	1683	A vs G	1.30(0.95-1.80)	0.100	<0.001	All	5	805	1255	G vs A	0.69(0.50-0.94)	0.020	0.001	Included 1 studies published in Chinese
53	GSTM1	Present/Null	30976200	10.1186/s12935-019-0792-3	[67]	Li	2019	PubMed, Web of Science, Embase, and CNKI	July-18	All	41	6124	9781	null vs present	1.37(1.18-1.59)	<0.001	<0.001	All	24	4209	6971	null vs present	1.13(0.92-1.39)	0.238	0.000	Included 21 studies published in Chinese
54	GSTT1	Present/Null	30976200	10.1186/s12935-019-0792-4	[67]	Li	2019	PubMed, Web of Science, Embase, and CNKI	July-18	All	30	4836	7670	null vs present	1.43(1.23-1.66)	<0.001	<0.001	All	20	3801	6060	null vs present	1.21(0.99-1.46)	0.052	0.000	Included 14 studies published in Chinese

8.Reference in Supplemental Table 2

1. Wu, X., et al., A 3' UTR SNP in COL18A1 is associated with susceptibility to HBV related hepatocellular carcinoma in Chinese: three independent case-control studies. PLoS One, 2012. 7(3): p. e33855.
2. Zhang, Y.J., et al., Polymorphisms in cyclin D1 gene and hepatocellular carcinoma. Mol Carcinog, 2002. 33(2): p. 125-9.
3. Zeng, Z., et al., Influence of CCND1 G870A polymorphism on the risk of HBV-related HCC and cyclin D1 splicing variant expression in Chinese population. Tumour Biol, 2015. 36(9): p. 6891-900.
4. Hu, Z., et al., Cyclin D1 G870A polymorphism and the risk of hepatocellular carcinoma in a Chinese population. Tumour Biol, 2014. 35(6): p. 5607-12.
5. Elkhashab, M.N., et al., Association between Cyclin D1 G870A Polymorphism and the Risk of Hepatocellular Carcinoma among Egyptian Population. Afro-Egyptian Journal of Infectious and Endemic Diseases, 2015. 5(4): p. 297-304.
6. Akkiz, H., et al., Cyclin D1 G870A polymorphism is associated with an increased risk of hepatocellular carcinoma in the Turkish population: case-control study. Cancer Epidemiol, 2010. 34(3): p. 298-302.
7. Liu, Y., et al., Association of PvuII and XbaI polymorphisms in estrogen receptor alpha gene with the risk of hepatitis B virus infection in the Guangxi Zhuang population. Infect Genet Evol, 2014. 27: p. 69-76.
8. Zhai, Y., et al., Estrogen receptor alpha polymorphisms associated with susceptibility to hepatocellular carcinoma in hepatitis B virus carriers. Gastroenterology, 2006. 130(7): p. 2001-9.
9. Anghel, A., et al., Estrogen receptor alpha polymorphisms and the risk of malignancies. Pathol Oncol Res, 2010. 16(4): p. 485-96.
10. Balasus, D., et al., The association of variants in PNPLA3 and GRP78 and the risk of developing hepatocellular carcinoma in an Italian population. Oncotarget, 2016. 7(52): p. 86791-86802.
11. Tomoda, T., et al., Genetic risk of hepatocellular carcinoma in patients with hepatitis C virus: a case control study. J Gastroenterol Hepatol, 2012. 27(4): p. 797-804.
12. Zhu, X., et al., Single nucleotide polymorphism of rs430397 in the fifth intron of GRP78 gene and clinical relevance of primary hepatocellular carcinoma in Han Chinese: risk and prognosis. Int J Cancer, 2009. 125(6): p. 1352-7.
13. Abo-Hashem, E.M., et al., Genetic Polymorphisms of Cytochrome P4501A1 (CYP1A1) and Glutathione S-Transferase P1 (GSTP1) and Risk of Hepatocellular Carcinoma Among Chronic Hepatitis C Patients in Egypt. Biochem Genet, 2016. 54(5): p. 696-713.
14. Chen, Y.L., et al., Glutathione S-Transferase P1 (GSTP1) gene

polymorphism increases age-related susceptibility to hepatocellular carcinoma. Bmc Medical Genetics, 2010. 11.
15. Ladero, J.M., et al., Glutathlone S-transferases pi 1, alpha I and mu 3 genetic polymorphisms and the risk of hepatocellular carcinoma in humans. Pharmacogenomics, 2007. 8(8): p. 895-899.
16. Li, C.G., et al., Predictive role of glutathione-S-transferase gene polymorphisms in risk and prognosis of hepatocellular carcinoma. Asian Pac J Cancer Prev, 2012. 13(7): p. 3247-52.
17. Elofey, S.G.H., et al., Relation between GSTP1 polymorphism and oxidative stress in patients with hepatocellular carcinoma. J Egypt Natl Canc Inst, 2020. 32(1): p. 38.
18. Tian, M., et al., Gene-environment interactions between GSTs polymorphisms and targeted epigenetic alterations in hepatocellular carcinoma following organochlorine pesticides (OCPs) exposure. Environ Int, 2020. 134: p. 105313.
19. Munaka, M., et al., Genetic polymorphisms of tobacco- and alcohol-related metabolizing enzymes and the risk of hepatocellular carcinoma. Journal of Cancer Research and Clinical Oncology, 2003. 129(6): p. 355-360.
20. Beckman, L.E., et al., Interaction between haemochromatosis and transferrin receptor genes in hepatocellular carcinoma. Oncology, 2000. 59(4): p. 317-22.
21. Boige, V., et al., Lack of association between HFE gene mutations and hepatocellular carcinoma in patients with cirrhosis. Gut, 2003. 52(8): p. 1178-81.
22. Cauza, E., et al., Mutations of the HFE gene in patients, with hepatocellular carcinoma. American Journal of Gastroenterology, 2003. 98(2): p. 442-447.
23. Ezzikouri, S., et al., Prevalence of common HFE and SERPINA1 mutations in patients with hepatocellular carcinoma in a Moroccan population. Arch Med Res, 2008. 39(2): p. 236-41.
24. Gharib, A.F., et al., Polymorphisms of hemochromatosis, and alpha-1 antitrypsin genes in Egyptian HCV patients with and without hepatocellular carcinoma. Gene, 2011. 489(2): p. 98-102.
25. Hellerbrand, C., et al., HFE C282Y heterozygosity in hepatocellular carcinoma: evidence for an increased prevalence. Clin Gastroenterol Hepatol, 2003. 1(4): p. 279-84.
26. Lauret, E., et al., HFE gene mutations in alcoholic and virus-related cirrhotic patients with hepatocellular carcinoma. Am J Gastroenterol, 2002. 97(4): p. 1016-21.
27. Nahon, P., et al., Liver iron, HFE gene mutations, and hepatocellular carcinoma occurrence in patients with cirrhosis. Gastroenterology, 2008. 134(1): p. 102-10.
28. Racchi, O., et al., Mutations of the HFE gene and the risk of

hepatocellular carcinoma. Blood Cells Mol Dis, 1999. 25(5-6): p. 350-3.
29. Yonal, O., et al., HFE gene mutation, chronic liver disease, and iron overload In Turkey. Dig Dis Sci, 2007. 52(11): p. 3298-302.
30. Willis, G., et al., Hepatocellular carcinoma and the penetrance of HFE C282Y mutations: a cross sectional study. BMC Gastroenterol, 2005. 5: p. 17.
31. Campo, S., et al., Analysis of haemochromatosis gene mutations in a population from the Mediterranean Basin. Liver, 2001. 21(4): p. 233-6.
32. Neghina, A.M., et al., Mutant HFE genotype leads to significant iron overload in patients with liver diseases from western Romania. J Appl Genet, 2009. 50(2): p. 173-6.
33. Ropero, P., et al., [The H63D mutation in the HFE gene is related to the risk of hepatocellular carcinoma]. Rev Esp Enferm Dig, 2007. 99(7): p. 376-81.
34. Hu, L., et al., Genetic variants in human leukocyte antigen/DP-DQ influence both hepatitis B virus clearance and hepatocellular carcinoma development. Hepatology, 2012. 55(5): p. 1426-31.
35. Al-Qahtani, A.A., et al., Association between HLA variations and chronic hepatitis B virus infection in Saudi Arabian patients. PLoS One, 2014. 9(1): p. e80445.
36. An, P., et al., A common HLA-DPA1 variant is a major determinant of hepatitis B virus clearance in Han Chinese. J Infect Dis, 2011. 203(7): p. 943-7.
37. Liao, Y., et al., Association of HLA-DP/DQ and STAT4 polymorphisms with HBV infection outcomes and a mini meta-analysis. PLoS One, 2014. 9(11): p. e111677.
38. Li, J., et al., Associations of HLA-DP variants with hepatitis B virus infection in southern and northern Han Chinese populations: a multicenter case-control study. PLoS One, 2011. 6(8): p. e24221.
39. Gao, X., et al., Genetic polymorphism of HLA-DQ confers susceptibility to hepatitis B virus-related hepatocellular carcinoma: a case-control study in Han population in China. Tumour Biol, 2016. 37(9): p. 12103-12111.
40. Zhang, X., et al., HLA-DQ polymorphisms with HBV infection: different outcomes upon infection and prognosis to lamivudine therapy. J Viral Hepat, 2014. 21(7): p. 491-8.
41. Liu, Y., et al., A genetic variant in long non-coding RNA HULC contributes to risk of HBV-related hepatocellular carcinoma in a Chinese population. PLoS One, 2012. 7(4): p. e35145.
42. Motawi, T.M.K., et al., The expression of long non coding RNA genes is associated with expression with polymorphisms of HULC rs7763881 and MALAT1 rs619586 in hepatocellular carcinoma and HBV Egyptian patients. Journal of Cellular Biochemistry, 2019. 120(9): p. 14645-14656.

43. Wang, B.G., et al., The association of lncRNA-HULC polymorphisms with hepatocellular cancer risk and prognosis. Gene, 2018. 670: p. 148-154.
44. Tak, K.H., et al., Association Between Polymorphisms of Interleukin 1 Family Genes and Hepatocellular Carcinoma. Med Sci Monit, 2018. 24: p. 3488-3495.
45. Sakamoto, T., et al., Interaction between interleukin-1beta -31T/C gene polymorphism and drinking and smoking habits on the risk of hepatocellular carcinoma among Japanese. Cancer Lett, 2008. 271(1): p. 98-104.
46. Migita, K., et al., Polymorphisms of interleukin-1beta in Japanese patients with hepatitis B virus infection. J Hepatol, 2007. 46(3): p. 381-6.
47. Wang, Y., et al., Interieukin-1 beta gene polymorphisms associated with hepatocellular carcinoma in hepatitis C virus infection. Hepatology, 2003. 37(1): p. 65-71.
48. Chen, C.C., et al., Association of cytokine and DNA repair gene polymorphisms with hepatitis B-related hepatocellular carcinoma. Int J Epidemiol, 2005. 34(6): p. 1310-8.
49. Okamoto, K., et al., The genotypes of IL-1 beta and MMP-3 are associated with the prognosis of HCV-related hepatocellular carcinoma. Intern Med, 2010. 49(10): p. 887-95.
50. Wang, Y., et al., UDP-glucuronosyltransferase 1A7 genetic polymorphisms are associated with hepatocellular carcinoma in japanese patients with hepatitis C virus infection. Clin Cancer Res, 2004. 10(7): p. 2441-6.
51. Falleti, E., et al., Interleukin-6 polymorphisms and gender: relationship with the occurrence of hepatocellular carcinoma in patients with end-stage liver disease. Oncology, 2009. 77(5): p. 304-13.
52. Ognjanovic, S., et al., Genetic polymorphisms in the cytokine genes and risk of hepatocellular carcinoma in low-risk non-Asians of USA. Carcinogenesis, 2009. 30(5): p. 758-762.
53. Migita, K., et al., Cytokine gene polymorphisms in Japanese patients with hepatitis B virus infection--association between TGF-beta1 polymorphisms and hepatocellular carcinoma. J Hepatol, 2005. 42(4): p. 505-10.
54. Badawy, A.A., et al., IL-6 -572G/C and -174G/C polymorphisms association with hepatitis C virus-induced hepatocellular carcinoma. Br J Biomed Sci, 2019. 76(4): p. 201-204.
55. Giannitrapani, L., et al., IL-6 -174G/C polymorphism and IL-6 serum levels in patients with liver cirrhosis and hepatocellular carcinoma. Omics, 2011. 15(3): p. 183-6.
56. Chien, M.H., et al., Relationship of Interleukin-8 Gene Polymorphisms With Hepatocellular Carcinoma Susceptibility and Pathological

Development. Journal of Surgical Oncology, 2011. 104(7): p. 798-803.
57. Qin, X., et al., The IL-8 gene polymorphisms and the risk of the hepatitis B virus/infected patients. DNA Cell Biol, 2012. 31(6): p. 1125-30.
58. Wang, J.L., et al., Association of interleukin-8 gene polymorphisms with the risk of hepatocellular carcinoma. Molecular Biology Reports, 2014. 41(3): p. 1483-1489.
59. Al-Qahtani, A., et al., Correlation between genetic variations and serum level of interleukin 28B with virus genotypes and disease progression in chronic hepatitis C virus infection. J Immunol Res, 2015. 2015: p. 768470.
60. Fabris, C., et al., IL-28B rs12979860 C/T allele distribution in patients with liver cirrhosis: role in the course of chronic viral hepatitis and the development of HCC. J Hepatol, 2011. 54(4): p. 716-22.
61. Chen, J., et al., Association analysis between SNPs in IL-28B gene and the progress of hepatitis B infection in Han Chinese. PLoS One, 2012. 7(12): p. e50787.
62. Eurich, D., et al., Role of IL28B polymorphism in the development of hepatitis C virus-induced hepatocellular carcinoma, graft fibrosis, and posttransplant antiviral therapy. Transplantation, 2012. 93(6): p. 644-9.
63. Moreira, J.P., et al., Interferon lambda and hepatitis C virus core protein polymorphisms associated with liver cancer. Virology, 2016. 493: p. 136-41.
64. Agúndez, J.A., et al., Relation of IL28B gene polymorphism with biochemical and histological features in hepatitis C virus-induced liver disease. PLoS One, 2012. 7(5): p. e37998.
65. Ibrahim, M.K., et al., Three Gene Signature for Predicting the Development of Hepatocellular Carcinoma in Chronically Infected Hepatitis C Virus Patients. J Interferon Cytokine Res, 2016. 36(12): p. 698-705.
66. Shi, X., et al., IL28B is associated with outcomes of chronic HBV infection. Yonsei Med J, 2015. 56(3): p. 625-33.
67. Ren, S., et al., Genetic variation in IL28B is associated with the development of hepatitis B-related hepatocellular carcinoma. Cancer Immunol Immunother, 2012. 61(9): p. 1433-9.
68. De Re, V., et al., HCV-related liver and lymphoproliferative diseases: association with polymorphisms of IL28B and TLR2. Oncotarget, 2016. 7(25): p. 37487-37497.
69. Lee, M.H., et al., Polymorphisms near the IFNL3 Gene Associated with HCV RNA Spontaneous Clearance and Hepatocellular Carcinoma Risk. Sci Rep, 2015. 5: p. 17030.
70. Kimkong, I., et al., Gene polymorphisms of interleukin 28B and the risk to chronic hepatitis B virus infection in Thai. Tissue Antigens, 2015. 85(3): p. 177-81.

71. Chang, K.C., et al., Risk factors for development of hepatocellular carcinoma in patients with chronic hepatitis C without sustained response to combination therapy. J Formos Med Assoc, 2018. 117(11): p. 1011-1018.
72. Chang, K.C., et al., A polymorphism in interferon L3 is an independent risk factor for development of hepatocellular carcinoma after treatment of hepatitis C virus infection. Clin Gastroenterol Hepatol, 2015. 13(5): p. 1017-24.
73. Bochud, P.Y., et al., IL28B alleles associated with poor hepatitis C virus (HCV) clearance protect against inflammation and fibrosis in patients infected with non-1 HCV genotypes. Hepatology, 2012. 55(2): p. 384-94.
74. de la Fuente, S., et al., Interleukin-28B TT genotype is frequently found in patients with hepatitis C virus cirrhosis but does not influence hepatocarcinogenesis. Clin Exp Med, 2017. 17(2): p. 217-223.
75. Su, C., et al., Association between polymorphisms in tumor suppressor genes and oncogenes and risk of hepatocellular carcinoma: a case-control study in an HCC epidemic area within the Han Chinese population. Med Oncol, 2014. 31(12): p. 356.
76. Dharel, N., et al., MDM2 promoter SNP309 is associated with the risk of hepatocellular carcinoma in patients with chronic hepatitis C. Clinical Cancer Research, 2006. 12(16): p. 4867-4871.
77. Yoon, Y.J., et al., MDM2 and p53 polymorphisms are associated with the development of hepatocellular carcinoma in patients with chronic hepatitis B virus infection. Carcinogenesis, 2008. 29(6): p. 1192-6.
78. Qiu, M., et al., Interaction between p53 codon 72 and MDM2 309T>G polymorphisms and the risk of hepatocellular carcinoma. Tumour Biol, 2016. 37(3): p. 3863-70.
79. Wang, X., et al., MDM2 SNP309T>G polymorphism increases susceptibility to hepatitis B virus-related hepatocellular carcinoma in a northeast Han Chinese population. Liver International, 2012. 32(7): p. 1172-1178.
80. Yang, Y., et al., Combined effects of p53 and MDM2 polymorphisms on susceptibility and surgical prognosis in hepatitis B virus-related hepatocellular carcinoma. Protein Cell, 2013. 4(1): p. 71-81.
81. Acun, T., et al., Mdm2 Snp309 G allele displays high frequency and inverse correlation with somatic P53 mutations in hepatocellular carcinoma. Mutat Res, 2010. 684(1-2): p. 106-8.
82. Akkiz, H., et al., MDM2 promoter polymorphism is associated with increased susceptibility to hepatocellular carcinoma in Turkish population. Cancer Epidemiol, 2010. 34(4): p. 448-52.
83. Di Vuolo, V., et al., TP53 and MDM2 gene polymorphisms and risk of hepatocellular carcinoma among Italian patients. Infectious Agents and Cancer, 2011. 6.

84. Ezzikouri, S., et al., MDM2 SNP309T>G polymorphism and risk of hepatocellular carcinoma: a case-control analysis in a Moroccan population. Cancer Detect Prev, 2009. 32(5-6): p. 380-5.
85. Leu, J.D., et al., Association between MDM2-SNP309 and hepatocellular carcinoma in Taiwanese population. World J Gastroenterol, 2009. 15(44): p. 5592-7.
86. Son, M.S., et al., Promoter polymorphisms of pri-miR-34b/c are associated with hepatocellular carcinoma. Gene, 2013. 524(2): p. 156-60.
87. Han, Y., et al., Associations of pri-miR-34b/c and pre-miR-196a2 polymorphisms and their multiplicative interactions with hepatitis B virus mutations with hepatocellular carcinoma risk. PLoS One, 2013. 8(3): p. e58564.
88. Xu, Y., et al., A potentially functional polymorphism in the promoter region of miR-34b/c is associated with an increased risk for primary hepatocellular carcinoma. Int J Cancer, 2011. 128(2): p. 412-7.
89. Chen, L.L., et al., Association between polymorphisms in the promoter region of pri-miR-34b/c and risk of hepatocellular carcinoma. Genet Mol Res, 2016. 15(4).
90. Liu, C.J., et al., pri-miR-34b/c rs4938723 polymorphism is associated with hepatocellular carcinoma risk: a case-control study in a Chinese population. Int J Mol Epidemiol Genet, 2017. 8(1): p. 1-7.
91. Zhao, H., et al., Association between the NQO1 C609T polymorphism with hepatocellular carcinoma risk in the Chinese population. Asian Pac J Cancer Prev, 2015. 16(5): p. 1821-5.
92. Nahon, P., et al., Genetic dimorphism in superoxide dismutase and susceptibility to alcoholic cirrhosis, hepatocellular carcinoma, and death. Clin Gastroenterol Hepatol, 2005. 3(3): p. 292-8.
93. Nahon, P., et al., Manganese superoxide dismutase dimorphism and iron overload, hepatocellular carcinoma, and death in hepatitis C virus-infected patients. Clin Gastroenterol Hepatol, 2007. 5(5): p. 630-5.
94. Ezzikouri, S., et al., Genetic polymorphism in the manganese superoxide dismutase gene is associated with an increased risk for hepatocellular carcinoma in HCV-infected Moroccan patients. Mutat Res, 2008. 649(1-2): p. 1-6.
95. Akkiz, H., et al., No association of NAD(P)H: quinone oxidoreductase 1 (NQO1) C609T polymorphism and risk of hepatocellular carcinoma development in Turkish subjects. Asian Pac J Cancer Prev, 2010. 11(4): p. 1051-8.
96. Liu, F., et al., A functional NQO1 609C > T polymorphism and risk of hepatocellular carcinoma in a Chinese population. Tumor Biology, 2013. 34(1): p. 47-53.
97. Ma, H.X., et al., Association of NQO1 and ESR1 polymorphisms with hepatocellular carcinoma susceptibility. International Journal of Clinical

and Experimental Pathology, 2016. 9(6): p. 6536-6542.
98. Ma, J., et al., TGF-β1 polymorphism 509 C>T is associated with an increased risk for hepatocellular carcinoma in HCV-infected patients. Genet Mol Res, 2015. 14(2): p. 4461-8.
99. Yang, Y., et al., TNF-alpha -863 polymorphisms and the risk of hepatocellular carcinoma. Exp Ther Med, 2012. 3(3): p. 513-518.
100. Xin, Z., et al., Polymorphisms in the potential functional regions of the TGF-β 1 and TGF-β receptor genes and disease susceptibility in HBV-related hepatocellular carcinoma patients. Mol Carcinog, 2012. 51 Suppl 1: p. E123-31.
101. Qi, P., et al., -509C>T polymorphism in the TGF-beta1 gene promoter, impact on the hepatocellular carcinoma risk in Chinese patients with chronic hepatitis B virus infection. Cancer Immunol Immunother, 2009. 58(9): p. 1433-40.
102. Wan, P.Q., et al., TGF-β1 polymorphisms and familial aggregation of liver cancer in Guangxi, China. Genet Mol Res, 2015. 14(3): p. 8147-60.
103. Saxena, R., et al., Effect of IL-12B, IL-2, TGF-β1, and IL-4 polymorphism and expression on hepatitis B progression. J Interferon Cytokine Res, 2014. 34(2): p. 117-28.
104. Falleti, E., et al., TGF-beta1 genotypes in cirrhosis: relationship with the occurrence of liver cancer. Cytokine, 2008. 44(2): p. 256-61.
105. Radwan, M.I., et al., Influence of transforming growth factor-β1 and tumor necrosis factor-α genes polymorphisms on the development of cirrhosis and hepatocellular carcinoma in chronic hepatitis C patients. Cytokine, 2012. 60(1): p. 271-6.
106. Heneghan, M.A., et al., Frequency and nature of cytokine gene polymorphisms in hepatocellular carcinoma in Hong Kong Chinese. Int J Gastrointest Cancer, 2003. 34(1): p. 19-26.
107. Jeng, J.E., et al., Tumor necrosis factor-alpha 308.2 polymorphism is associated with advanced hepatic fibrosis and higher risk for hepatocellular carcinoma. Neoplasia, 2007. 9(11): p. 987-92.
108. Kummee, P., et al., Association of HLA-DRB1*13 and TNF-alpha gene polymorphisms with clearance of chronic hepatitis B infection and risk of hepatocellular carcinoma in Thai population. J Viral Hepat, 2007. 14(12): p. 841-8.
109. Akkiz, H., et al., G-308A TNF-alpha polymorphism is associated with an increased risk of hepatocellular carcinoma in the Turkish population: case-control study. Cancer Epidemiol, 2009. 33(3-4): p. 261-4.
110. Wang, B., et al., A study of TNF-alpha-238 and -308 polymorphisms with different outcomes of persistent hepatitis B virus infection in China. Pathology, 2010. 42(7): p. 674-80.
111. Chen, X., et al., Association of TNF-alpha genetic polymorphisms with hepatocellular carcinoma susceptibility: a case-control study in a Han

Chinese population. Int J Biol Markers, 2011. 26(3): p. 181-7.
112. Shi, Z. and C. Du, Tumor necrosis factor alpha 308 G/A polymorphism and hepatocellular carcinoma risk in a Chinese population. Genet Test Mol Biomarkers, 2011. 15(7-8): p. 569-72.
113. Teixeira, A.C., et al., Alleles and genotypes of polymorphisms of IL-18, TNF-α and IFN-γ are associated with a higher risk and severity of hepatocellular carcinoma (HCC) in Brazil. Hum Immunol, 2013. 74(8): p. 1024-9.
114. Sghaier, I., et al., The relationship between TNF alpha gene polymorphisms (-238/-308), TNF RII VNTR (p75) and outcomes of hepatitis B virus infection in Tunisian population. Gene, 2015. 568(2): p. 140-5.
115. Shin, S.P., et al., Association between hepatocellular carcinoma and tumor necrosis factor alpha polymorphisms in South Korea. World J Gastroenterol, 2015. 21(46): p. 13064-72.
116. Yang, M.D., et al., Tumor Necrosis Factor-α Genotypes Are Associated with Hepatocellular Carcinoma Risk in Taiwanese Males, Smokers and Alcohol Drinkers. Anticancer Res, 2015. 35(10): p. 5417-23.
117. Tharwat, E., et al., Impact of IL-27p28 (rs153109) and TNF-α (rs1800629) Genetic Polymorphisms on the Progression of HCV Infection in Egyptian Patients. Immunol Invest, 2019. 48(3): p. 255-267.
118. Talaat, R.M., et al., Tumor necrosis factor-alpha -308G/A polymorphism and risk of hepatocellular carcinoma in hepatitis C virus-infected patients. Chin J Cancer, 2012. 31(1): p. 29-35.
119. Li, Y., et al., The ERCC1-4533/8092, TNF-α 238/308 polymorphisms and the risk of hepatocellular carcinoma in Guangxi Zhuang populations of China: Case-control study. Medicine (Baltimore), 2016. 95(44): p. e5217.
120. Yue, X., et al., Association of hepatocellular carcinoma risk with polymorphisms in tumour necrosis factor alpha gene in a Chinese Han population. Int J Immunogenet, 2020. 47(3): p. 286-293.
121. Jung, K.W., et al., TNFalpha promoter polymorphism is a risk factor for susceptibility in hepatocellular carcinoma in Korean population. Clin Chim Acta, 2009. 407(1-2): p. 16-9.
122. Panigrahi, R., et al., Association of TNF-alpha Promoter Polymorphism with HBV Associated Disease Outcome Among HBV Infected Patients from Orissa, Southern Part of East India. J Clin Exp Hepatol, 2014. 4(3): p. 202-8.
123. Qiu, B., et al., Association of TNF-alpha promoter polymorphisms with the outcome of persistent HBV infection in a northeast Chinese Han population. Acta Biochim Biophys Sin (Shanghai), 2012. 44(8): p. 712-8.
124. Niro, G.A., et al., Tumor necrosis factor gene polymorphisms and clearance or progression of hepatitis B virus infection. Liver Int, 2005.

25(6): p. 1175-81.
125. Chen, D.N., et al., Gene polymorphisms of TLR2 and TLR3 in HBV clearance and HBV-related hepatocellular carcinoma in a Chinese male population. International Journal of Biological Markers, 2017. 32(2): p. E195-E201.
126. Huo, Z.C., et al., Association of genetic polymorphisms in toll-like receptor 2 (TLR2) and susceptibility to hepatocellular carcinoma. Biomedical Research-India, 2017. 28(1): p. 369-373.
127. Neamatallah, M., et al., Impact of Toll-like Receptors 2(TLR2) and TLR 4 Gene Variations on HCV Susceptibility, Response to Treatment and Development of Hepatocellular Carcinoma in Cirrhotic HCV Patients. Immunological Investigations, 2020. 49(4): p. 462-476.
128. Wu, J.S., et al., Implication of polymorphisms in DNA repair genes with an increased risk of hepatocellular carcinoma. Genet Mol Res, 2014. 13(2): p. 3812-8.
129. Long, X.D., et al., XPD codon 312 and 751 polymorphisms, and AFB1 exposure, and hepatocellular carcinoma risk. BMC Cancer, 2009. 9: p. 400.
130. Guo, L.Y., et al., Association of XPD and XRCC1 genetic polymorphisms with hepatocellular carcinoma risk. Asian Pac J Cancer Prev, 2012. 13(9): p. 4423-6.
131. Yuan, T., et al., Relationship between XRCC1 and XPD polymorphisms and the risk of the development of hepatocellular carcinoma: A case-control study. Exp Ther Med, 2012. 4(2): p. 285-290.
132. Gulnaz, A., et al., Association of XRCC1, XRCC3, and XPD genetic polymorphism with an increased risk of hepatocellular carcinoma because of the hepatitis B and C virus. European Journal of Gastroenterology & Hepatology, 2013. 25(2): p. 166-179.
133. Krupa, R., et al., The Relationship Between Single-Nucleotide Polymorphisms, the Expression of DNA Damage Response Genes, and Hepatocellular Carcinoma in a Polish Population. DNA Cell Biol, 2017. 36(8): p. 693-708.
134. Balkan, E., et al., ERCC2 Lys751Gln rs13181 and XRCC2 Arg188His rs3218536 gene polymorphisms contribute to susceptibility of colon, gastric, liver, lung and prostate cancer. Journal of Buon, 2020. 25(1): p. 574-581.
135. Yao, J.G., X.Y. Huang, and X.D. Long, Interaction of DNA repair gene polymorphisms and aflatoxin B1 in the risk of hepatocellular carcinoma. Int J Clin Exp Pathol, 2014. 7(9): p. 6231-44.
136. Naguib, M., et al., XRCC1 Gene Polymorphism Increases the Risk of Hepatocellular Carcinoma in Egyptian Population. Asian Pac J Cancer Prev, 2020. 21(4): p. 1031-1037.
137. Arafa, M., et al., Genetic variants of XRCC1 and risk of hepatocellular carcinoma in chronic hepatitis C patients. Br J Biomed Sci, 2019. 76(2):

p. 64-69.
138. Bi, J.W., et al., Association Study of Single Nucleotide Polymorphisms in XRCC1 Gene with Risk of Hepatocellular Carcinoma in Chinese Han Population. Biomed Research International, 2013. 2013.
139. Kiran, M., et al., Polymorphism of DNA repair gene XRCC1 and hepatitis-related hepatocellular carcinoma risk in Indian population. Molecular and Cellular Biochemistry, 2009. 327(1-2): p. 7-13.
140. Mattar, M.M., et al., Polymorphisms of base-excision repair genes and the hepatocarcinogenesis. Gene, 2018. 675: p. 62-68.
141. Yang, Z. and J. Zhao, Effect of APE1 and XRCC1 gene polymorphism on susceptibility to hepatocellular carcinoma and sensitivity to cisplatin. Int J Clin Exp Med, 2015. 8(6): p. 9931-6.
142. Zeng, X., et al., DNA repair capacity, DNA-strand break repair gene polymorphisms, and the incidence of hepatocellular carcinoma in southwestern Guangxi of China. DNA Cell Biol, 2012. 31(8): p. 1384-91.
143. Kao, C.C., et al., Influence of glutathione-S-transferase theta (GSTT1) and micro (GSTM1) gene polymorphisms on the susceptibility of hepatocellular carcinoma in Taiwan. J Surg Oncol, 2010. 102(4): p. 301-7.
144. Long, X.D., et al., The polymorphisms of GSTM1, GSTT1, HYL1*2, and XRCC1, and aflatoxin B1-related hepatocellular carcinoma in Guangxi population, China. Hepatol Res, 2006. 36(1): p. 48-55.
145. Deng, Z.L., Y.P. Wei, and Y. Ma, Polymorphism of glutathione S-transferase mu 1 and theta 1 genes and hepatocellular carcinoma in southern Guangxi, China. World J Gastroenterol, 2005. 11(2): p. 272-4.
146. Yu, M.W., et al., Polymorphisms in XRCC1 and glutathione S-transferase genes and hepatitis B-related hepatocellular carcinoma. J Natl Cancer Inst, 2003. 95(19): p. 1485-8.
147. Yu, M.W., et al., Plasma carotenoids, glutathione S-transferase M1 and T1 genetic polymorphisms, and risk of hepatocellular carcinoma: independent and interactive effects. Am J Epidemiol, 1999. 149(7): p. 621-9.
148. Sun, C.A., et al., Genetic polymorphisms of glutathione S-transferases M1 and T1 associated with susceptibility to aflatoxin-related hepatocarcinogenesis among chronic hepatitis B carriers: a nested case-control study in Taiwan. Carcinogenesis, 2001. 22(8): p. 1289-94.
149. Bian, J.C., et al., Susceptibility to hepatocellular carcinoma associated with null genotypes of GSTM1 and GSTT1. World J Gastroenterol, 2000. 6(2): p. 228-230.
150. Kirk, G.D., et al., Hepatocellular carcinoma and polymorphisms in carcinogen-metabolizing and DNA repair enzymes in a population with aflatoxin exposure and hepatitis B virus endemicity. Cancer Epidemiology Biomarkers & Prevention, 2005. 14(2): p. 373-379.

151. Araujo, O.C., et al., Association of Polymorphisms in the Glutathione S-Transferase Theta-1 Gene with Cirrhosis and Hepatocellular Carcinoma in Brazilian Patients with Chronic Hepatitis C. Vaccines (Basel), 2021. 9(8).
152. Tiemersma, E.W., et al., Role of genetic polymorphism of glutathione-S-transferase T1 and microsomal epoxide hydrolase in aflatoxin-associated hepatocellular carcinoma. Cancer Epidemiol Biomarkers Prev, 2001. 10(7): p. 785-91.
153. Asim, M., et al., Genetic polymorphism of glutathione S transferases M1 and T1 in Indian patients with hepatocellular carcinoma. Dis Markers, 2010. 28(6): p. 369-76.
154. Kiran, M., Y.K. Chawla, and J. Kaur, Glutathione-S-transferase and microsomal epoxide hydrolase polymorphism and viral-related hepatocellular carcinoma risk in India. DNA Cell Biol, 2008. 27(12): p. 687-94.
155. Sarma, M.P., et al., Hepatitis C virus related hepatocellular carcinoma: a case control study from India. J Med Virol, 2012. 84(7): p. 1009-17.
156. Boccia, S., et al., The effect of CYP, GST, and SULT polymorphisms and their interaction with smoking on the risk of hepatocellular carcinoma. Biomed Res Int, 2015. 2015: p. 179867.
157. Abd El-Moneim, E., et al., Gene deletion of glutathione S-transferase M1 and T1 and risk factors of hepatocellular carcinoma in Egyptian patients. Egypt J Immunol, 2008. 15(2): p. 125-34.
158. Gelatti, U., et al., N-Acetyltransferase-2, glutathione S-transferase M1 and T1 genetic polymorphisms, cigarette smoking and hepatocellular carcinoma: a case-control study. Int J Cancer, 2005. 115(2): p. 301-6.
159. Ladero, J.M., et al., Glutathione S-transferase M1 and T1 genetic polymorphisms are not related to the risk of hepatocellular carcinoma: A study in the Spanish population. European Journal of Cancer, 2006. 42(1): p. 73-77.
160. Kong, S.Y., et al., UGT1A7 haplotype is associated with an increased risk of hepatocellular carcinoma in hepatitis B carriers. Cancer Sci, 2008. 99(2): p. 340-4.
161. Stucker, I., et al., UDP-glucuronosyltransferase UGT1A7 genetic polymorphisms in hepatocellular carcinoma: a differential impact according to seropositivity of HBV or HCV markers? BMC Cancer, 2007. 7: p. 214.
162. Borentain, P., et al., DNA-repair and carcinogen-metabolising enzymes genetic polymorphisms as an independent risk factor for hepatocellular carcinoma in Caucasian liver-transplanted patients. Eur J Cancer, 2007. 43(17): p. 2479-86.
163. Tseng, C.S., et al., UDP-glucuronosyltransferase 1A7 genetic polymorphisms are associated with hepatocellular carcinoma risk and onset age. Am J Gastroenterol, 2005. 100(8): p. 1758-63.

164. Vogel, A., et al., Genetic link of hepatocellular carcinoma with polymorphisms of the UDP-glucuronosyltransferase UGT1A7 gene. Gastroenterology, 2001. 121(5): p. 1136-44.
165. De Mattia, E., et al., UGT1A polymorphisms as genetic biomarkers for hepatocellular carcinoma risk in Caucasian population. Liver Int, 2017. 37(9): p. 1345-1353.
166. Li, Y., et al., Association between epidermal growth factor 61A/G polymorphism and hepatocellular carcinoma susceptibility in Chinese patients. Liver Int, 2010. 30(1): p. 112-8.
167. Qi, P., et al., No association of EGF 5'UTR variant A61G and hepatocellular carcinoma in Chinese patients with chronic hepatitis B virus infection. Pathology, 2009. 41(6): p. 555-60.
168. Chen, K., et al., Epidermal growth factor +61 G/A polymorphism and the risk of hepatocellular carcinoma in a Chinese population. Genet Test Mol Biomarkers, 2011. 15(4): p. 251-5.
169. Abbas, E., et al., Epidermal growth factor gene polymorphism 61A/G in patients with chronic liver disease for early detection of hepatocellular carcinoma: a pilot study. Eur J Gastroenterol Hepatol, 2012. 24(4): p. 458-63.
170. Yuan, J.M., et al., Genetic polymorphisms of epidermal growth factor in relation to risk of hepatocellular carcinoma: two case-control studies. Bmc Gastroenterology, 2013. 13.
171. Wu, J., et al., Association of epidermal growth factor and epidermal growth factor receptor polymorphisms with the risk of hepatitis B virus-related hepatocellular carcinoma in the population of North China. Genet Test Mol Biomarkers, 2013. 17(8): p. 595-600.
172. Asar, S., et al., Epidermal growth factor gene polymorphism in hepatitis C virus patients with hepatocellular carcinoma. Journal of Hepatology, 2020. 73: p. S326-S327.
173. Tanabe, K.K., et al., Epidermal growth factor gene functional polymorphism and the risk of hepatocellular carcinoma in patients with cirrhosis. Jama, 2008. 299(1): p. 53-60.
174. Abu Dayyeh, B.K., et al., A functional polymorphism in the epidermal growth factor gene is associated with risk for hepatocellular carcinoma. Gastroenterology, 2011. 141(1): p. 141-9.
175. Cmet, S., et al., Carriage of the EGF rs4444903 A > G functional polymorphism associates with disease progression in chronic HBV infection. Clinical and Experimental Immunology, 2012. 167(2): p. 296-302.
176. El-Bendary, M., et al., Epidermal growth factor genetic polymorphism predicts risk of hepatocellular carcinoma in Egyptian patients with HCV (genotype 4)-related cirrhosis. Journal of Hepatology, 2013. 58: p. S261.
177. Suenaga, M., et al., Investigation of a functional polymorphism in the

epidermal growth factor gene for pathogenesis and prognosis of hepatocellular carcinoma in Japanese patients. Cancer Research, 2013. 73(8).
178. McGlynn, K.A., et al., SUSCEPTIBILITY TO HEPATOCELLULAR-CARCINOMA IS ASSOCIATED WITH GENETIC-VARIATION IN THE ENZYMATIC DETOXIFICATION OF AFLATOXIN B-1. Proceedings of the National Academy of Sciences of the United States of America, 1995. 92(6): p. 2384-2387.
179. Wong, N.A., et al., Polymorphisms of the gene for microsomal epoxide hydrolase and susceptibility to alcoholic liver disease and hepatocellular carcinoma in a Caucasian population. Toxicol Lett, 2000. 115(1): p. 17-22.
180. Tiemersma, E.W., et al., Role of genetic polymorphism of glutathione-S-transferase T1 and microsomal epoxide hydrolase in aflatoxin-associated hepatocellular carcinoma. Cancer Epidemiology Biomarkers & Prevention, 2001. 10(7): p. 785-791.
181. Sonzogni, L., et al., Polymorphisms of microsomal epoxide hydrolase gene and severity of HCV-related liver disease. Hepatology, 2002. 36(1): p. 195-201.
182. Kiran, M., Y.K. Chawla, and J. Kaur, Glutathione-S-Transferase and Microsomal Epoxide Hydrolase Polymorphism and Viral-Related Hepatocellular Carcinoma Risk in India. DNA and Cell Biology, 2008. 27(12): p. 687-694.
183. Long, X.D., et al., The polymorphisms of GSTM1, GSTT1, HYL1*2, and XRCC1, and aflatoxin B1-related hepatocellular carcinoma in Guangxi population, China. Hepatology Research, 2006. 36(1): p. 48-55.
184. Mah, Y.H., et al., Prevalence and clinical implications of HFE gene mutations (C282Y and H63D) in patients with chronic hepatitis B and C in Taiwan. Liver Int, 2005. 25(2): p. 214-9.
185. Motawi, T.K., et al., Genetic variants associated with the progression of hepatocellular carcinoma in hepatitis C Egyptian patients. Gene, 2013. 527(2): p. 516-520.
186. Fargion, S., et al., Mutations in the HFE gene and their interaction with exogenous risk factors in hepatocellular carcinoma. Blood Cells Mol Dis, 2001. 27(2): p. 505-11.
187. Hirankarn, N., et al., Interleukin-1beta gene polymorphism associated with hepatocellular carcinoma in hepatitis B virus infection. World J Gastroenterol, 2006. 12(5): p. 776-9.
188. Tanaka, Y., et al., Impact of interleukin-1beta genetic polymorphisms on the development of hepatitis C virus-related hepatocellular carcinoma in Japan. J Infect Dis, 2003. 187(11): p. 1822-5.
189. Saxena, R., et al., Interleukin-1 polymorphism and expression in hepatitis b virus mediated disease outcome in India. Indian Journal of Clinical Biochemistry, 2015. 30: p. S106.

190. Bochud, P.Y., et al., IL28B alleles associated with poor HCV clearance are protective against liver necroinflammatory activity and fibrosis progression in patients infected with non-1 HCV genotypes. Hepatology, 2011. 54: p. 404A.
191. Joshita, S., et al., Association of IL28B gene polymorphism with development of hepatocellular carcinoma in Japanese patients with chronic hepatitis C virus infection. Hum Immunol, 2012. 73(3): p. 298-300.
192. Ma, N., et al., Role of IFN-ks, IFN-ks related genes and the DEPDC5 gene in Hepatitis B virus-related liver disease. J Viral Hepat, 2014. 21(7): p. e29-38.
193. Hagiwara, S., et al., Risk of hepatocellular carcinoma development in cases of hepatitis C treated by long-term, low-dose PEG-IFNα-2a. Dig Dis, 2012. 30(6): p. 561-7.
194. Miura, M., et al., Analysis of viral amino acids sequences and the IL28B SNP influencing the development of hepatocellular carcinoma in chronic hepatitis C. Hepatol Int, 2012. 6(1): p. 386-96.
195. Asahina, Y., et al., Genetic variation near interleukin 28B and the risk of hepatocellular carcinoma in patients with chronic hepatitis C. J Gastroenterol, 2014. 49(7): p. 1152-62.
196. Farokhizadeh, Z., et al., Association of MicroRNA Polymorphisms With Hepatocellular Carcinoma in an Iranian Population. Ann Lab Med, 2019. 39(1): p. 58-66.
197. Abdel-Hamid, M., S. Elshaer, and A. Darwish, Association of MicroRNA related single nucleotide polymorphisms 196A-2 and 499 with the risk of hepatocellular carcinoma in Egyptian patients. Meta Gene, 2018. 16: p. 139-142.
198. Zhang, L.H., et al., Contributions of polymorphisms in miR146a, miR196a, and miR499 to the development of hepatocellular carcinoma. Genetics and Molecular Research, 2016. 15(3).
199. Toraih, E.A., et al., MicroRNA-196a2 Biomarker and Targetome Network Analysis in Solid Tumors. Molecular Diagnosis & Therapy, 2016. 20(6): p. 559-577.
200. Yan, P., et al., Predictive role of miR-146a rs2910164 (C>G), miR-149 rs2292832 (T>C), miR-196a2 rs11614913 (T>C) and miR-499 rs3746444 (T>C) in the development of hepatocellular carcinoma. Int J Clin Exp Pathol, 2015. 8(11): p. 15177-83.
201. Li, X.H., K. Li, and Z.J. Wu, Association of four common SNPs in microRNA polymorphisms with the risk of hepatocellular carcinoma. International Journal of Clinical and Experimental Pathology, 2015. 8(8): p. 9560-9566.
202. Qi, J.H., et al., High-resolution melting analysis reveals genetic polymorphisms in microRNAs confer hepatocellular carcinoma risk in Chinese patients. BMC Cancer, 2014. 14: p. 643.

203. Qi, P., et al., Association of a variant in MIR 196A2 with susceptibility to hepatocellular carcinoma in male Chinese patients with chronic hepatitis B virus infection. Human Immunology, 2010. 71(6): p. 621-626.
204. Zhou, B., et al., Association between miR-146aG>C and miR-196a2C>T polymorphisms and the risk of hepatocellular carcinoma in a Chinese population. Tumour Biol, 2014. 35(8): p. 7775-80.
205. Chu, Y.H., et al., MicroRNA gene polymorphisms and environmental factors increase patient susceptibility to hepatocellular carcinoma. PLoS One, 2014. 9(2): p. e89930.
206. Hao, Y.X., J.P. Wang, and L.F. Zhao, Associations between three common MicroRNA polymorphisms and hepatocellular carcinoma risk in Chinese. Asian Pac J Cancer Prev, 2014. 14(11): p. 6601-4.
207. Zhang, J., et al., Association Between Single Nucleotide Polymorphisms in miRNA196a-2 and miRNA146a and Susceptibility to Hepatocellular Carcinoma in a Chinese Population. Asian Pacific Journal of Cancer Prevention, 2013. 14(11): p. 6427-6431.
208. Kim, H.Y., et al., Association of polymorphism in microRNA-196A-2 with occurrence of hepatocellular carcinoma. Hepatology, 2012. 56: p. 629A.
209. Akkız, H., et al., A functional polymorphism in pre-microRNA-196a-2 contributes to the susceptibility of hepatocellular carcinoma in a Turkish population: a case-control study. J Viral Hepat, 2011. 18(7): p. e399-407.
210. Han, Y.F., et al., Associations of pri-miR-34b/c and pre-miR-196a2 Polymorphisms and Their Multiplicative Interactions with Hepatitis B Virus Mutations with Hepatocellular Carcinoma Risk. Plos One, 2013. 8(3).
211. Borentain, P., et al., DNA-repair and carcinogen-metabolising enzymes genetic polymorphisms as an independent risk factor for hepatocellular carcinoma in Caucasian liver-transplanted patients. Eur J Cancer, 2007. 43(17): p. 2479-86.
212. Jia, Z.F., et al., Polymorphisms of UGT1A7 and XRCC1 are associated with an increased risk of hepatocellular carcinoma in Northeast China. Chinese Journal of Cancer Research, 2010. 22(4): p. 260-266.
213. Pan, H.Z., et al., Polymorphism of DNA repair gene XRCC1 and hepatocellular carcinoma risk in Chinese population. Asian Pac J Cancer Prev, 2011. 12(11): p. 2947-50.
214. Bose, S., et al., Genetic polymorphisms of CYP2E1 and DNA repair genes HOGG1 and XRCC1: Association with hepatitis B related advanced liver disease and cancer. Gene, 2013. 519(2): p. 231-237.
215. Mohana Devi, S., et al., Analysis of genetic damage and gene polymorphism in hepatocellular carcinoma (HCC) patients in a South Indian population. Dig Dis Sci, 2013. 58(3): p. 759-67.

216. Wu, D., et al., Association between XRCC1 Arg399Gln polymorphism and hepatitis virus-related hepatocellular carcinoma risk in Asian population. Tumour Biol, 2013. 34(5): p. 3265-9.
217. He, S., D.C. Zhang, and C. Wei, MicroRNAs as biomarkers for hepatocellular carcinoma diagnosis and prognosis. Clinics and Research in Hepatology and Gastroenterology, 2015. 39(4): p. 426-434.
218. Santonocito, C., et al., XRCC1 Arg399Gln gene polymorphism and hepatocellular carcinoma risk in the Italian population. Int J Biol Markers, 2017. 32(2): p. e190-e194.
219. Mahmoud, A.A., et al., Urinary 8-hydroxydeoxyguanosine in relation to XRCC 1 rs25487 G/A (Arg399Gln) and OGG 1 rs 1052133 C/G (Ser326Cys) DNA repair genes polymorphisms in patients with chronic hepatitis C and related hepatocellular carcinoma. Cancer Management and Research, 2019. 11: p. 5343-5351.
220. Li, Q.W., et al., Evaluation of DNA repair gene XRCC1 polymorphism in prediction and prognosis of hepatocellular carcinoma risk. Asian Pac J Cancer Prev, 2012. 13(1): p. 191-4.
221. Zeng, X.Y., et al., Meta-analysis demonstrates lack of a relationship between XRCC1-399 gene polymorphisms and susceptibility to hepatocellular carcinoma. Genet Mol Res, 2013. 12(2): p. 1916-23.

1. Zuo W, Zhan Z, Ma L, Bai W, Zeng S. Effect of ALDH2 polymorphism on cancer risk in Asians: A meta-analysis. Medicine (Baltimore). 2019;98(13):e14855. doi:10.1097/MD.0000000000014855 Add to Citavi project by DOI
2. Zhou D, Xiao L, Zhang Y, et al. Genetic polymorphisms of ALDH2 and ADH2 are not associated with risk of hepatocellular carcinoma among East Asians. Tumour Biol. 2012;33(3):841-846.
3. Chen J, Pan W, Chen Y, Wen L, Tu J, Liu K. Relationship of ALDH2 rs671 and CYP2E1 rs2031920 with hepatocellular carcinoma susceptibility in East Asians: a meta-analysis. World J Surg Oncol. 2020;18(1):21.
4. Zhu Y, Li Y, Zhu S, Tang R, Liu Y, Li J. Association of survivin polymorphisms with tumor susceptibility: a meta-analysis. PLoS One. 2013;8(9):e74778.
5. Jin C, Wu X, Gu Y, et al. CD95 rs1800682A/G variant and tumor risk in Asians: evidence from a meta-analysis of 36 case-control studies containing 22,438 samples. Med Sci Monit. 2015;21:630-637.
6. Chen Z, Zhu J, Huang C, Lian F, Wu G, Zhao Y. The association between three cyclooxygenase-2 polymorphisms and hepatocellular carcinoma risk: a meta-analysis. PLoS One. 2015;10(3):e0118251.
7. Lu SC, Zhong JH, Tan JT, et al. Association between COX-2 gene polymorphisms and risk of hepatocellular carcinoma development: a meta-analysis. BMJ Open. 2015;5(10):e008263.
8. Wang C, Liu W, Zhao L, Dong Z. Association of cytotoxic T-lymphocyte antigen-4 + 49A/G gene polymorphism with hepatocellular carcinoma risk in Chinese. J Cancer Res Ther. 2018;14(Supplement):S1117-S1120.
9. Zhu S, Zhang H, Tang Y, Liu P, Wang J. DNMT3B polymorphisms and cancer risk: a meta analysis of 24 case-control studies. Mol Biol Rep. 2012;39(4):4429-4437.
10. Zhang Y, Zhang J, Shen N, Ren K: EGF rs4444903a allele may decrease hepatocellular carcinoma risk in chinese individuals: A meta-analysis. International Journal of Clinical and Experimental Medicine 2016, **9**(3):5719-5727.
11. Jiang G, Yu K, Shao L, et al. Association between epidermal growth factor gene +61A/G polymorphism and the risk of hepatocellular carcinoma: a meta-analysis based on 16 studies. BMC Cancer. 2015;15:314.
12. Duan CY, Liu MY, Li SB, Ma KS, Bie P. Lack of association of EPHX1 gene polymorphisms with risk of hepatocellular carcinoma: a meta-analysis. Tumour Biol. 2014;35(1):659-666.
13. Sun H, Deng Q, Pan Y, et al. Association between estrogen receptor 1 (ESR1) genetic variations and cancer risk: a meta-analysis. J BUON. 2015;20(1):296-308.
14. Zhao Y, Wang Q, Deng X, Shi P, Wang Z. Quantitative assessment of the

association between GSTP1 gene Ile105Val polymorphism and susceptibility to hepatocellular carcinoma. Tumour Biol. 2013;34(4):2121-2126.
15. Ye Q, Qian BX, Yin WL, Wang FM, Han T. Association between the HFE C282Y, H63D Polymorphisms and the Risks of Non-Alcoholic Fatty Liver Disease, Liver Cirrhosis and Hepatocellular Carcinoma: An Updated Systematic Review and Meta-Analysis of 5,758 Cases and 14,741 Controls. PLoS One. 2016;11(9):e0163423.
16. Liao Y, Cai B, Li Y, et al. Association of HLA-DP/DQ and STAT4 polymorphisms with HBV infection outcomes and a mini meta-analysis. PLoS One. 2014;9(11): e111677.
17. Xu T, Zhu A, Sun M, et al. Quantitative assessment of HLA-DQ gene polymorphisms with the development of hepatitis B virus infection, clearance, liver cirrhosis, and hepatocellular carcinoma. Oncotarget. 2017;9(1):96-109.
18. Coelho AV, Moura RR, Crovella S, Celsi F. HLA-G genetic variants and hepatocellular carcinoma: a meta-analysis. Genet Mol Res. 2016;15(3):10.4238/gmr.15038263.
19. Kang SW, Kim SK, Park HJ, Chung JH, Ban JY. Human 8-oxoguanine DNA glycosylase gene polymorphism (Ser326Cys) and cancer risk: updated meta-analysis. Oncotarget. 2017;8(27):44761-44775.
20. Wang W, Dang S, Li Y, et al. hOGG1 Ser326Cys polymorphism and risk of hepatocellular carcinoma among East Asians: a meta-analysis. PLoS One. 2013;8(4):e60178.
21. Yang Y, Luo C, Feng R, Bi S. The TNF-α, IL-1B and IL-10 polymorphisms and risk for hepatocellular carcinoma: a meta-analysis. J Cancer Res Clin Oncol. 2011;137(6):947-952.
22. An PP, Feng LN, Zhang XX, Jin QL. Association of interleukin-6 gene polymorphisms with the risk of hepatocellular carcinoma: An up-to-date meta-analysis. Medicine (Baltimore). 2020;99(50):e23659.
23. Tian G, Mi J, Wei X, et al. Circulating interleukin-6 and cancer: A meta-analysis using Mendelian randomization. Sci Rep. 2015;5:11394.
24. Aleagha OE, Oltulu P, Sadeghi M. Association between interleukin 6 polymorphisms (rs1800796, rs1800795, rs2069837, rs17147230, and rs1800797) and hepatocellular carcinoma susceptibility: a meta-analysis. Clin Exp Hepatol. 2020;6(4):359-366.
25. Wang P, An J, Zhu Y, et al. Association of three promoter polymorphisms in interleukin-10 gene with cancer susceptibility in the Chinese population: a meta-analysis. Oncotarget. 2017;8(37):62382-62399.
26. Qin S, Wang J, Zhou C, et al. The influence of interleukin 28B polymorphisms on the risk of hepatocellular carcinoma among patients with HBV or HCV infection: An updated meta-analysis. Medicine (Baltimore). 2019;98(38):e17275.
27. Ni W, Wang X, Sun Y, Gao X. Meta-analysis of the association between

MALAT1 rs619586 A>G polymorphism and cancer risk. J Int Med Res. 2020;48(7):300060520941969.
28. Teo K, Abeysekera KWM, Adams L, et al. rs641738C>T near MBOAT7 is associated with liver fat, ALT and fibrosis in NAFLD: A meta-analysis. J Hepatol. 2021;74(1):20-30.
29. Zhang C, Ye Z, Zhang Z, et al. A comprehensive evaluation of single nucleotide polymorphisms associated with hepatocellular carcinoma risk in Asian populations: A systematic review and network meta-analysis. Gene. 2020;735:144365.
30. Duan X, Li J. Association between MDM2 SNP309, p53 Arg72Pro, and hepatocellular carcinoma risk: A MOOSE-compliant meta-analysis. Medicine (Baltimore). 2017;96(36):e7856.
31. Su H, Zhang G. Correlation between Methylenetetrahydrofolate Reductase Polymorphisms and Hepatocellular Carcinoma: A Meta-Analysis. Ann Nutr Metab. 2019;74(3):251-256.
32. Chang W, Meng Q, Liu JH, Wu LX, Chen Y, Chen SD. Significant association between the MTHFR A1298C polymorphism and hepatocellular carcinoma risk: a meta-analysis. Genet Mol Res. 2015;14(4):15972-15980.
33. Liang TJ, Liu H, Zhao XQ, Tan YR, Jing K, Qin CY. Quantitative assessment of the association between MTHFR rs1801131 polymorphism and risk of liver cancer. Tumour Biol. 2014;35(1):339-343.
34. Sun H, Han B, Zhai H, Cheng X, Ma K. Significant association between MTHFR C677T polymorphism and hepatocellular carcinoma risk: a meta-analysis. Tumour Biol. 2014;35(1):189-193.
35. Park R, Lopes L, Saeed A. Association Between the Functional miR-146a SNP rs2910164 and Risk of Digestive System Cancer: Updated Meta-analysis. Anticancer Res. 2020;40(3):1495-1502.
36. Wang Z, Zhang L, Shi X, Xu H, Wang T, Bian J. Association between two common polymorphisms and risk of hepatocellular carcinoma: evidence from an updated meta-analysis. Biomed Res Int. 2014;2014:468605.
37. Zheng L, Zhuang C, Zhao J, Ming L. Functional miR-146a, miR-149, miR-196a2 and miR-499 polymorphisms and the susceptibility to hepatocellular carcinoma: An updated meta-analysis. Clin Res Hepatol Gastroenterol. 2017;41(6):664-676.
38. Zhang Q, Xu X, Wu M, Qin T, Wu S, Liu H. MiRNA Polymorphisms and Hepatocellular Carcinoma Susceptibility: A Systematic Review and Network Meta-Analysis. Front Oncol. 2021;10:562019.
39. Yan W, Gao X, Zhang S. Association of miR-196a2 rs11614913 and miR-499 rs3746444 polymorphisms with cancer risk: a meta-analysis. Oncotarget. 2017;8(69):114344-114359.
40. Wang BG, Jiang LY, Xu Q. Comprehensive assessment for miRNA polymorphisms in hepatocellular cancer risk: a systematic review and meta-analysis. Biosci Rep. 2018;38(5):BSR20180712.

41. Fan Y, Hu D, Feng B, Wang W. The NQO1 C609T polymorphism and hepatocellular carcinoma risk. Tumour Biol. 2014;35(8):7343-7350.
42. Zhao H, Zou LW, Zheng SS, Geng XP. Association between the NQO1 C609T polymorphism with hepatocellular carcinoma risk in the Chinese population. Asian Pac J Cancer Prev. 2015;16(5):1821-1825.
43. Zhang J, Xu F, Ouyang C. Joint effect of polymorphism in the N-acetyltransferase 2 gene and smoking on hepatocellular carcinoma. Tumour Biol. 2012;33(4):1059-1063.
44. Zhang C, Ye Z, Zhang Z, et al. A comprehensive evaluation of single nucleotide polymorphisms associated with hepatocellular carcinoma risk in Asian populations: A systematic review and network meta-analysis. Gene. 2020;735:144365.
45. Toshikuni N, Matsue Y, Minato T, Hayashi N, Tsutsumi M: Association between transforming growth factor-beta 1-509 C > T variants and hepatocellular carcinoma susceptibility: a meta-analysis. Neoplasma 2016, 63(6):961-966.
46. Li W, Wu H, Song C. TGF-β1 -509C/T (or +869T/C) polymorphism might be not associated with hepatocellular carcinoma risk. Tumour Biol. 2013;34(5):2675-2681.
47. Wang P, Wang J, Yu M, Li Z. Tumor Necrosis Factor-α T-857C (rs1799724) Polymorphism and Risk of Cancers: A Meta-Analysis. Dis Markers. 2016;2016:4580323.
48. Heidari Horestani M, Atri Roozbahani G, Sheidai M. The Potential Role of TNF-α (rs361525 and rs1800629) in Hepatocellular Carcinoma: Multivariate Analysis (Meta-Analysis). J Gastrointest Cancer. 2019;50(4):744-749.
49. Xiao Q, Fu B, Chen P, Liu ZZ, Wang W, Ye Q. Three polymorphisms of tumor necrosis factor-alpha and hepatitis B virus related hepatocellular carcinoma: A meta-analysis. Medicine (Baltimore). 2016;95(50):e5609.
50. Wungu CDK, Ariyanto FC, Prabowo GI, Soetjipto, Handajani R. Association between five types of Tumor Necrosis Factor-α gene polymorphism and hepatocellular carcinoma risk: a meta-analysis. BMC Cancer. 2020;20(1):1134. Published 2020 Nov 23.
51. Wei Y, Liu F, Li B, et al. Polymorphisms of tumor necrosis factor-alpha and hepatocellular carcinoma risk: a HuGE systematic review and meta-analysis. Dig Dis Sci. 2011;56(8):2227-2236.
52. Xiong Y, Zhang Q, Ye J, Pan S, Ge L. Associations between three XRCC1 polymorphisms and hepatocellular carcinoma risk: A meta-analysis of case-control studies. PLoS One. 2018;13(11):e0206853. Published 2018 Nov 8.
53. Li W, Yang F, Gui Y, Bian J. DNA repair gene XRCC1 Arg194Trp polymorphism and susceptibility to hepatocellular carcinoma: A meta-analysis. Oncol Lett. 2014;8(4):1725-1730.
54. Li J, Li Z, Feng L, Guo W, Zhang S. Polymorphisms of DNA repair gene

XRCC1 and hepatocellular carcinoma risk among East Asians: a meta-analysis. Tumour Biol. 2013;34(1):261-269.
55. Zhang XL, Lu Y, Yang S, et al. An updated meta-analysis between the association of XRCC1 Arg399Gln polymorphism and hepatocellular carcinoma risk. Asian Pac J Cancer Prev. 2014;15(7):3273-3278.
56. Qi Y, Cui L, Song Y, Li N. XRCC1 Arg399Gln genetic polymorphism and the risk of hepatocellular carcinoma: a meta-analysis. Mol Biol Rep. 2014;41(2):879-887.
57. Xu W, Liu SA, Li L, Shen ZY, Wu YL. Association between XRCC1 Arg280His polymorphism and risk of hepatocellular carcinoma: a systematic review and meta-analysis. Genet Mol Res. 2015;14(2):7122-7129.
58. Li LP, Wu W, Li XH, Song SS. The XRCC1 Arg280His gene polymorphism and hepatocellular carcinoma risk: a meta-analysis. Asian Pac J Cancer Prev. 2013;14(3):2033-2036.
59. Shi YH, Wang B, Xu BP, et al. The association of six non-synonymous variants in three DNA repair genes with hepatocellular carcinoma risk: a meta-analysis. J Cell Mol Med. 2016;20(11):2056-2063.
60. Wu D, Jiang H, Yu H, Xu D, Liang J, Jin J. Significant association between XRCC3 C241T polymorphism and increased risk of hepatocellular carcinoma: a meta-analysis. Tumour Biol. 2013;34(6):3865-3869.
61. Zhou Q, Fu Y, Wen L, Deng Y, Chen J, Liu K. XPD Polymorphisms and Risk of Hepatocellular Carcinoma and Gastric Cancer: A Meta-Analysis. Technol Cancer Res Treat. 2021; 20:1533033821990046.
62. Fu P, Yang F, Li B, et al. Meta-analysis of CYP2E1 polymorphisms in liver carcinogenesis. Dig Liver Dis. 2017;49(1):77-83. doi:10.1016/j.dld.2016.08.114
63. Chen J, Pan W, Chen Y, Wen L, Tu J, Liu K. Relationship of ALDH2 rs671 and CYP2E1 rs2031920 with hepatocellular carcinoma susceptibility in East Asians: a meta-analysis. World J Surg Oncol. 2020;18(1):21. Published 2020 Jan 27. doi:10.1186/s12957-020-1796-0
64. Zhang C, Ye Z, Zhang Z, et al. A comprehensive evaluation of single nucleotide polymorphisms associated with hepatocellular carcinoma risk in Asian populations: A systematic review and network meta-analysis. Gene. 2020;735:144365. doi:10.1016/j.gene.2020.144365
65. Zhu SL, Zhao Y, Hu XY, et al. Genetic polymorphisms -137 (rs187238) and -607 (rs1946518) in the interleukin-18 promoter may not be associated with development of hepatocellular carcinoma. Sci Rep. 2016;6:39404. Published 2016 Dec 21. doi:10.1038/srep39404
66. Luo T, Chen J, Liu JJ, et al. Association between cyclin D1 G870A polymorphism and hepatocellular carcinoma risk: a meta-analysis. Onco Targets Ther. 2016;9:4483-4489. Published 2016 Jul 21. doi:10.2147/OTT.S108754

67. Li S, Xue F, Zheng Y, et al. GSTM1 and GSTT1 null genotype increase the risk of hepatocellular carcinoma: evidence based on 46 studies. Cancer Cell Int. 2019;19:76. Published 2019 Mar 29. doi:10.1186/s12935-019-0792-3

10.PRISMA checklist

Section and Topic	Item #	Checklist item	Location where item is reported
**TITLE**			
Title	1	Identify the report as a systematic review.	Page 1
**ABSTRACT**			
Abstract	2	See the PRISMA 2020 for Abstracts checklist.	Page 3
**INTRODUCTION**			
Rationale	3	Describe the rationale for the review in the context of existing knowledge.	Page 4
Objectives	4	Provide an explicit statement of the objective(s) or question(s) the review addresses.	Page5
**METHODS**			
Eligibility criteria	5	Specify the inclusion and exclusion criteria for the review and how studies were grouped for the syntheses.	Page 20
Information sources	6	Specify all databases, registers, websites, organisations, reference lists and other sources searched or consulted to identify studies. Specify the date when each source was last searched or consulted.	Page 20
Search strategy	7	Present the full search strategies for all databases, registers and websites, including any filters and limits used.	Page 20
Selection process	8	Specify the methods used to decide whether a study met the inclusion criteria of the review, including how many reviewers screened each record and each report retrieved, whether they worked independently, and if applicable, details of automation tools used in the process.	Page 20
Data collection process	9	Specify the methods used to collect data from reports, including how many reviewers collected data from each report, whether they worked independently, any processes for obtaining or confirming data from study investigators, and if applicable, details of automation tools used in the process.	Page 21
Data items	10a	List and define all outcomes for which data were sought. Specify whether all results that were compatible with each outcome domain in each study were sought (e.g. for all measures, time points, analyses), and if not, the methods used to decide which results to collect.	Page 21
	10b	List and define all other variables for which data were sought (e.g. participant and intervention characteristics, funding sources). Describe any	Page 21

Section and Topic	Item #	Checklist item	Location where item is reported
		assumptions made about any missing or unclear information.	
Study risk of bias assessment	11	Specify the methods used to assess risk of bias in the included studies, including details of the tool(s) used, how many reviewers assessed each study and whether they worked independently, and if applicable, details of automation tools used in the process.	Page 22
Effect measures	12	Specify for each outcome the effect measure(s) (e.g. risk ratio, mean difference) used in the synthesis or presentation of results.	Page 22
Synthesis methods	13a	Describe the processes used to decide which studies were eligible for each synthesis (e.g. tabulating the study intervention characteristics and comparing against the planned groups for each synthesis (item #5)).	Page 22
	13b	Describe any methods required to prepare the data for presentation or synthesis, such as handling of missing summary statistics, or data conversions.	Page 22
	13c	Describe any methods used to tabulate or visually display results of individual studies and syntheses.	Page 22
	13d	Describe any methods used to synthesize results and provide a rationale for the choice(s). If meta-analysis was performed, describe the model(s), method(s) to identify the presence and extent of statistical heterogeneity, and software package(s) used.	Page 21
	13e	Describe any methods used to explore possible causes of heterogeneity among study results (e.g. subgroup analysis, meta-regression).	Page 16
	13f	Describe any sensitivity analyses conducted to assess robustness of the synthesized results.	Page 22
Reporting bias assessment	14	Describe any methods used to assess risk of bias due to missing results in a synthesis (arising from reporting biases).	Page 22
Certainty assessment	15	Describe any methods used to assess certainty (or confidence) in the body of evidence for an outcome.	Page 22-23
**RESULTS**			
Study selection	16a	Describe the results of the search and selection process, from the number of records identified in the search to the number of studies included in the review, ideally using a flow diagram.	Page 5
	16b	Cite studies that might appear to meet the inclusion criteria, but which were excluded, and explain why they were excluded.	Figure1

Section and Topic	Item #	Checklist item	Location where item is reported
Study characteristics	17	Cite each included study and present its characteristics.	Page 5-6
Risk of bias in studies	18	Present assessments of risk of bias for each included study.	Page 9
Results of individual studies	19	For all outcomes, present, for each study: (a) summary statistics for each group (where appropriate) and (b) an effect estimate and its precision (e.g. confidence/credible interval), ideally using structured tables or plots.	Page 6-9
Results of syntheses	20a	For each synthesis, briefly summarise the characteristics and risk of bias among contributing studies.	Page 6-9
	20b	Present results of all statistical syntheses conducted. If meta-analysis was done, present for each the summary estimate and its precision (e.g. confidence/credible interval) and measures of statistical heterogeneity. If comparing groups, describe the direction of the effect.	Page 6-9
	20c	Present results of all investigations of possible causes of heterogeneity among study results.	Page 9
	20d	Present results of all sensitivity analyses conducted to assess the robustness of the synthesized results.	Page 9
Reporting biases	21	Present assessments of risk of bias due to missing results (arising from reporting biases) for each synthesis assessed.	Page 9
Certainty of evidence	22	Present assessments of certainty (or confidence) in the body of evidence for each outcome assessed.	Page 10-11
**DISCUSSION**			
Discussion	23a	Provide a general interpretation of the results in the context of other evidence.	Page 11-16
	23b	Discuss any limitations of the evidence included in the review.	Page 19
	23c	Discuss any limitations of the review processes used.	Page 19
	23d	Discuss implications of the results for practice, policy, and future research.	Page 19
**OTHER INFORMATION**			
Registration and	24a	Provide registration information for the review, including register name and registration number, or state that the review was not registered.	Not

Section and Topic	Item #	Checklist item	Location where item is reported
protocol			available
	24b	Indicate where the review protocol can be accessed, or state that a protocol was not prepared.	Not available
	24c	Describe and explain any amendments to information provided at registration or in the protocol.	Not available
Support	25	Describe sources of financial or non-financial support for the review, and the role of the funders or sponsors in the review.	Page 1
Competing interests	26	Declare any competing interests of review authors.	Page 24
Availability of data, code and other materials	27	Report which of the following are publicly available and where they can be found: template data collection forms; data extracted from included studies; data used for all analyses; analytic code; any other materials used in the review.	Page 24

11. Forest plot of meta-analysis in Table 3

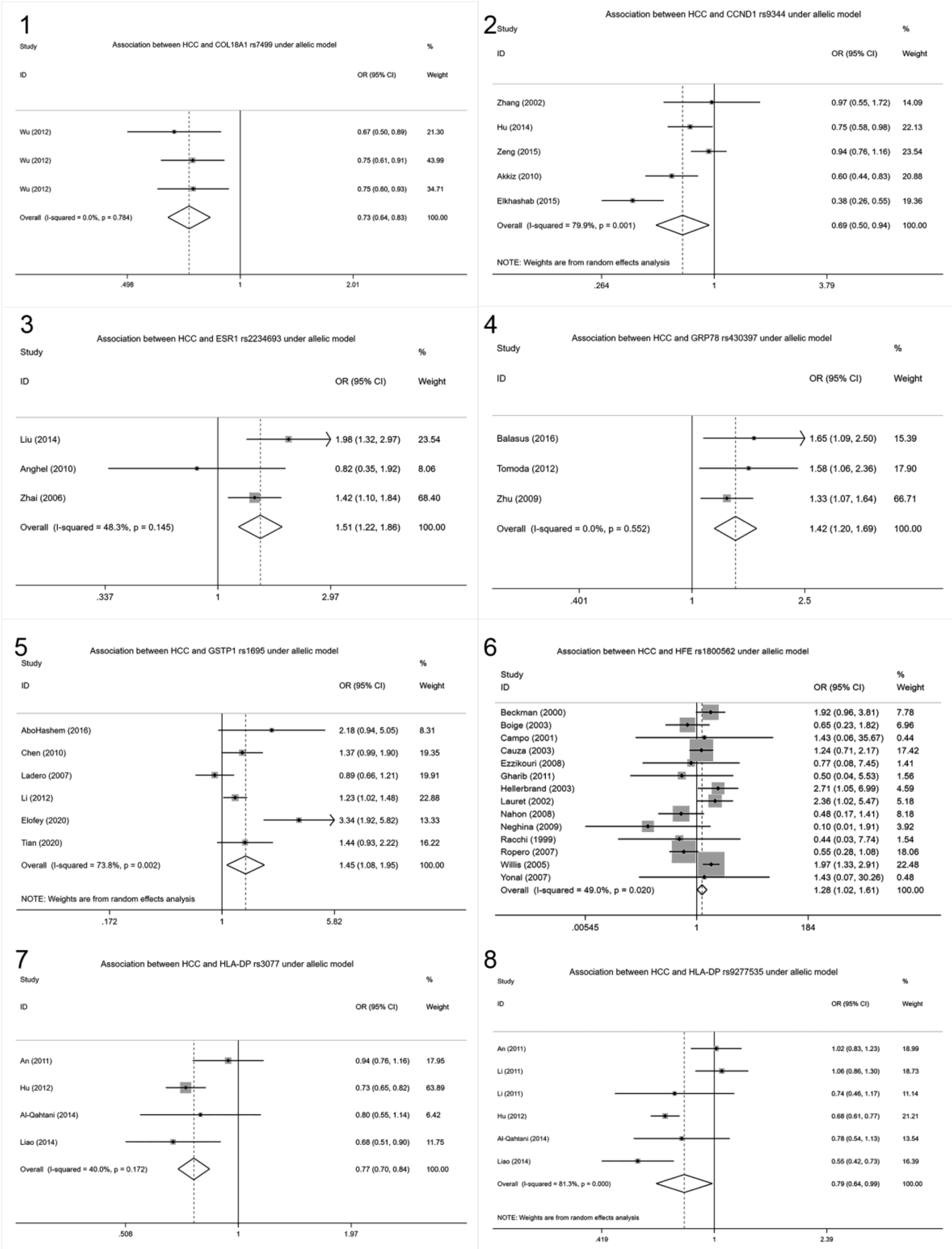

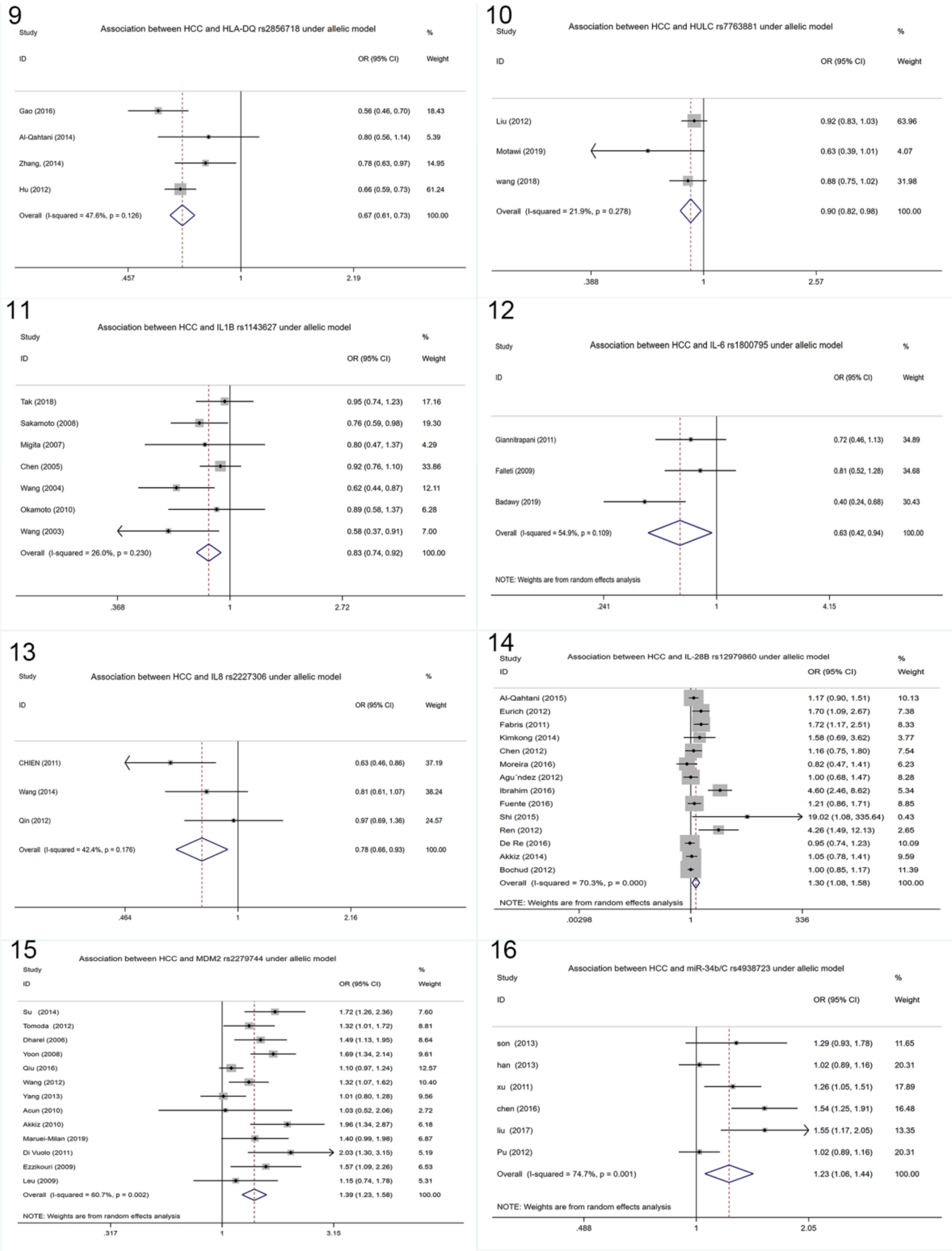
9
Association between HCC and HLA-DQ rs2856718 under allelic model
Study ID
OR (95% CI)
% Weight
Gao (2016) 0.56 (0.46, 0.70) 18.43
Al-Qahtani (2014) 0.80 (0.56, 1.14) 5.39
Zhang, (2014) 0.78 (0.63, 0.97) 14.95
Hu (2012) 0.66 (0.59, 0.73) 61.24
Overall (I-squared = 47.6%, p = 0.126) 0.67 (0.61, 0.73) 100.00
.457
1
2.19
10
Association between HCC and HULC rs7763881 under allelic model
Study ID
OR (95% CI)
% Weight
Liu (2012) 0.92 (0.83, 1.03) 63.96
Motawi (2019) 0.63 (0.39, 1.01) 4.07
wang (2018) 0.88 (0.75, 1.02) 31.98
Overall (I-squared = 21.9%, p = 0.278) 0.90 (0.82, 0.98) 100.00
.388
1
2.57
11
Association between HCC and IL1B rs1143627 under allelic model
Study ID
OR (95% CI)
% Weight
Tak (2018) 0.95 (0.74, 1.23) 17.16
Sakamoto (2008) 0.76 (0.59, 0.98) 19.30
Migita (2007) 0.80 (0.47, 1.37) 4.29
Chen (2005) 0.92 (0.76, 1.10) 33.86
Wang (2004) 0.62 (0.44, 0.87) 12.11
Okamoto (2010) 0.89 (0.58, 1.37) 6.28
Wang (2003) 0.58 (0.37, 0.91) 7.00
Overall (I-squared = 26.0%, p = 0.230) 0.83 (0.74, 0.92) 100.00
.368
1
2.72
12
Association between HCC and IL-6 rs1800795 under allelic model
Study ID
OR (95% CI)
% Weight
Giannitrapani (2011) 0.72 (0.46, 1.13) 34.89
Falleti (2009) 0.81 (0.52, 1.28) 34.68
Badawy (2019) 0.40 (0.24, 0.68) 30.43
Overall (I-squared = 54.9%, p = 0.109) 0.63 (0.42, 0.94) 100.00
NOTE: Weights are from random effects analysis
.241
1
4.15
13
Association between HCC and IL8 rs2227306 under allelic model
Study ID
OR (95% CI)
% Weight
CHIEN (2011) 0.63 (0.46, 0.86) 37.19
Wang (2014) 0.81 (0.61, 1.07) 38.24
Qin (2012) 0.97 (0.69, 1.36) 24.57
Overall (I-squared = 42.4%, p = 0.176) 0.78 (0.66, 0.93) 100.00
.464
1
2.16
14
Association between HCC and IL-28B rs12979860 under allelic model
Study ID
OR (95% CI)
% Weight
Al-Qahtani (2015) 1.17 (0.90, 1.51) 10.13
Eurich (2012) 1.70 (1.09, 2.67) 7.38
Fabris (2011) 1.72 (1.17, 2.51) 8.33
Kimkong (2014) 1.58 (0.69, 3.62) 3.77
Chen (2012) 1.16 (0.75, 1.80) 7.54
Moreira (2016) 0.82 (0.47, 1.41) 6.23
Agu´ndez (2012) 1.00 (0.68, 1.47) 8.28
Ibrahim (2016) 4.60 (2.46, 8.62) 5.34
Fuente (2016) 1.21 (0.86, 1.71) 8.85
Shi (2015) 19.02 (1.08, 335.64) 0.43
Ren (2012) 4.26 (1.49, 12.13) 2.65
De Re (2016) 0.95 (0.74, 1.23) 10.09
Akkiz (2014) 1.05 (0.78, 1.41) 9.59
Bochud (2012) 1.00 (0.85, 1.17) 11.39
Overall (I-squared = 70.3%, p = 0.000) 1.30 (1.08, 1.58) 100.00
NOTE: Weights are from random effects analysis
.00298
1
336
15
Association between HCC and MDM2 rs2279744 under allelic model
Study ID
OR (95% CI)
% Weight
Su (2014) 1.72 (1.26, 2.36) 7.60
Tomoda (2012) 1.32 (1.01, 1.72) 8.81
Dharel (2006) 1.49 (1.13, 1.95) 8.64
Yoon (2008) 1.69 (1.34, 2.14) 9.61
Qiu (2016) 1.10 (0.97, 1.24) 12.57
Wang (2012) 1.32 (1.07, 1.62) 10.40
Yang (2013) 1.01 (0.80, 1.28) 9.56
Acun (2010) 1.03 (0.52, 2.06) 2.72
Akkiz (2010) 1.96 (1.34, 2.87) 6.18
Maruei-Milan (2019) 1.40 (0.99, 1.98) 6.87
Di Vuolo (2011) 2.03 (1.30, 3.15) 5.19
Ezzikouri (2009) 1.57 (1.09, 2.26) 6.53
Leu (2009) 1.15 (0.74, 1.78) 5.31
Overall (I-squared = 60.7%, p = 0.002) 1.39 (1.23, 1.58) 100.00
NOTE: Weights are from random effects analysis
.317
1
3.15
16
Association between HCC and miR-34b/C rs4938723 under allelic model
Study ID
OR (95% CI)
% Weight
son (2013) 1.29 (0.93, 1.78) 11.65
han (2013) 1.02 (0.89, 1.16) 20.31
xu (2011) 1.26 (1.05, 1.51) 17.89
chen (2016) 1.54 (1.25, 1.91) 16.48
liu (2017) 1.55 (1.17, 2.05) 13.35
Pu (2012) 1.02 (0.89, 1.16) 20.31
Overall (I-squared = 74.7%, p = 0.001) 1.23 (1.06, 1.44) 100.00
NOTE: Weights are from random effects analysis
.488
1
2.05

17

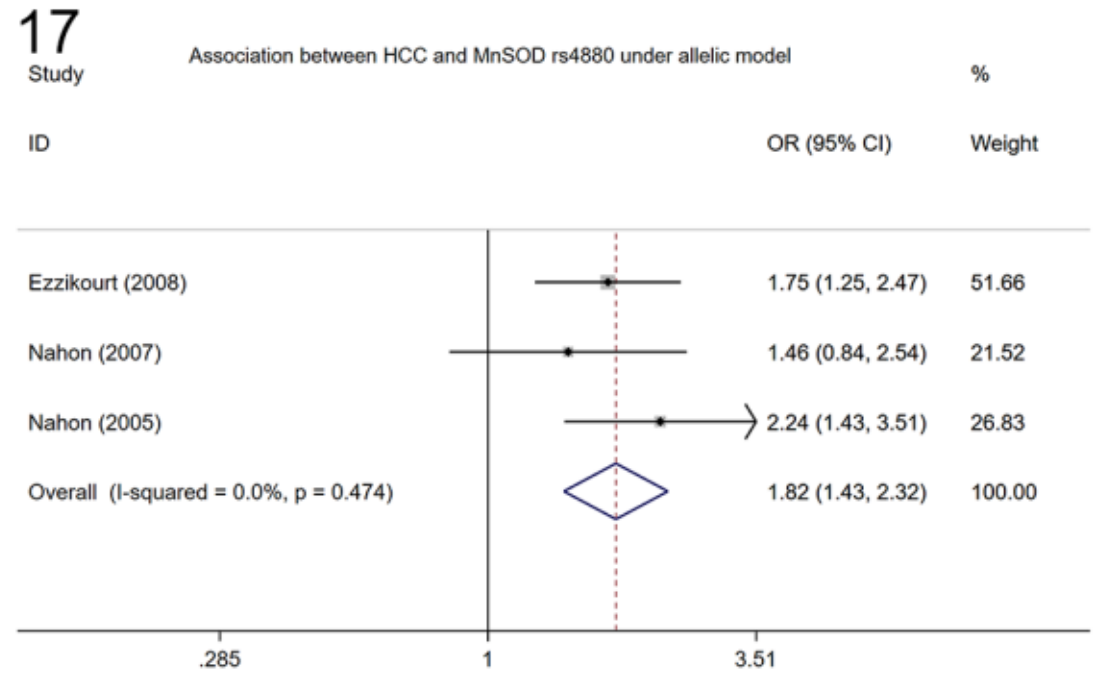

18

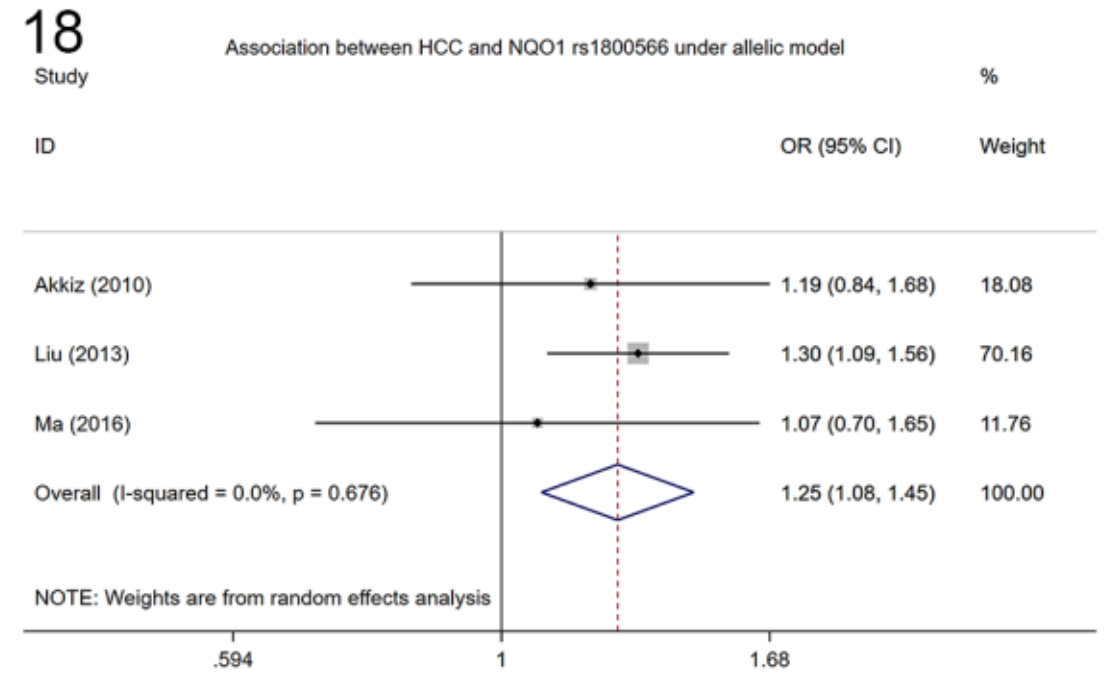

19

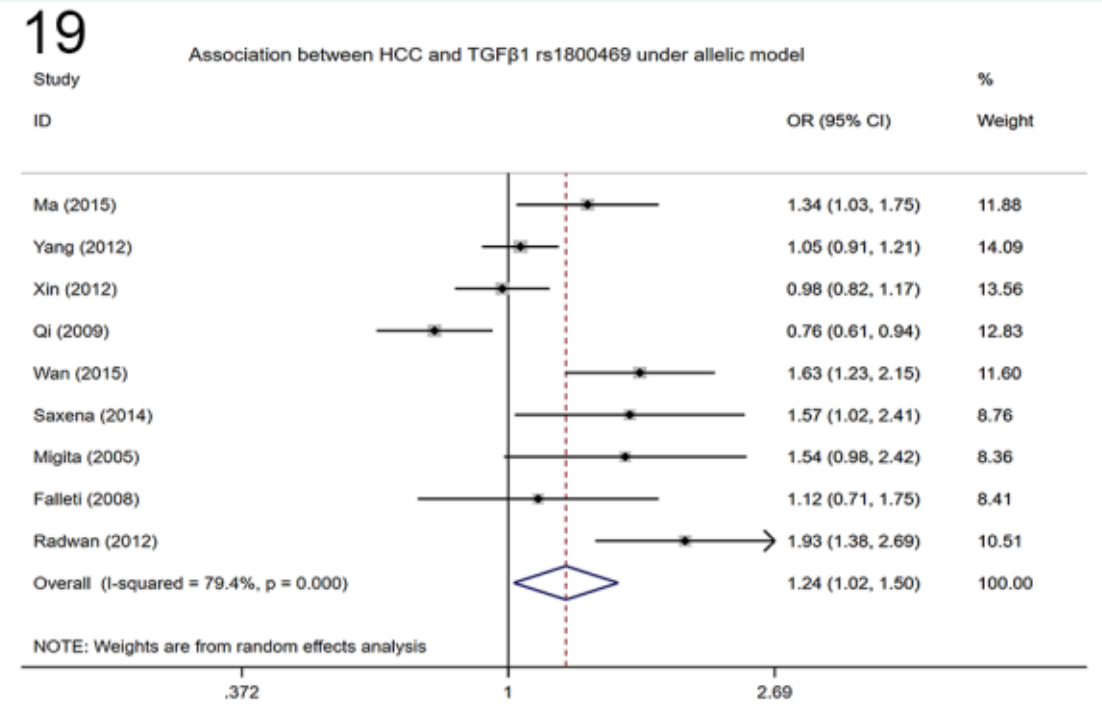

20

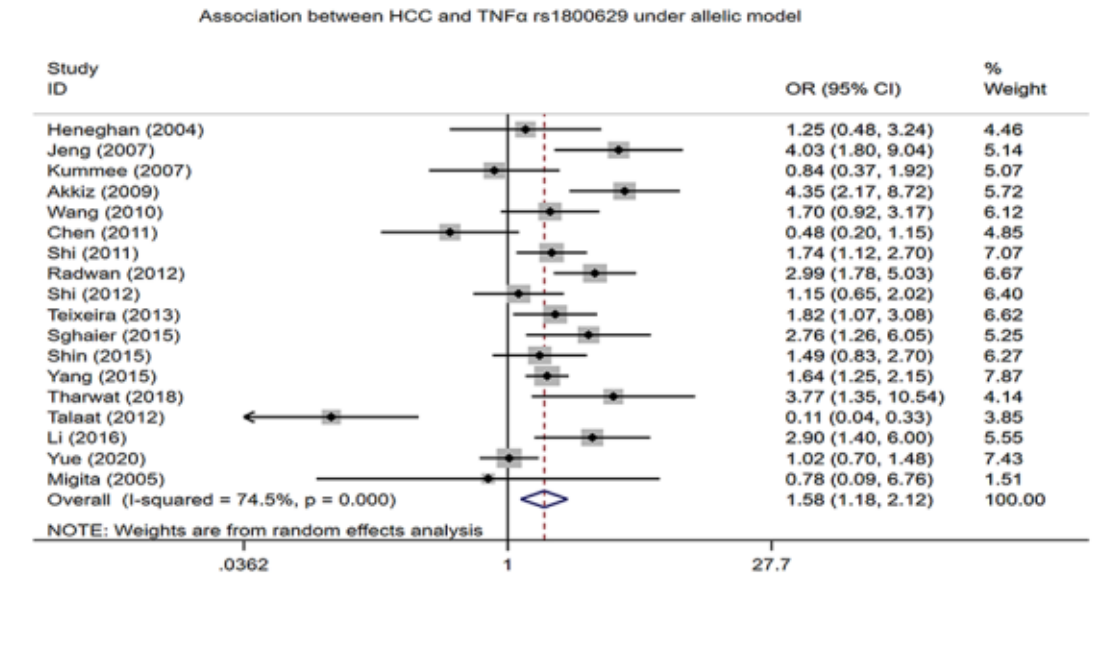

21

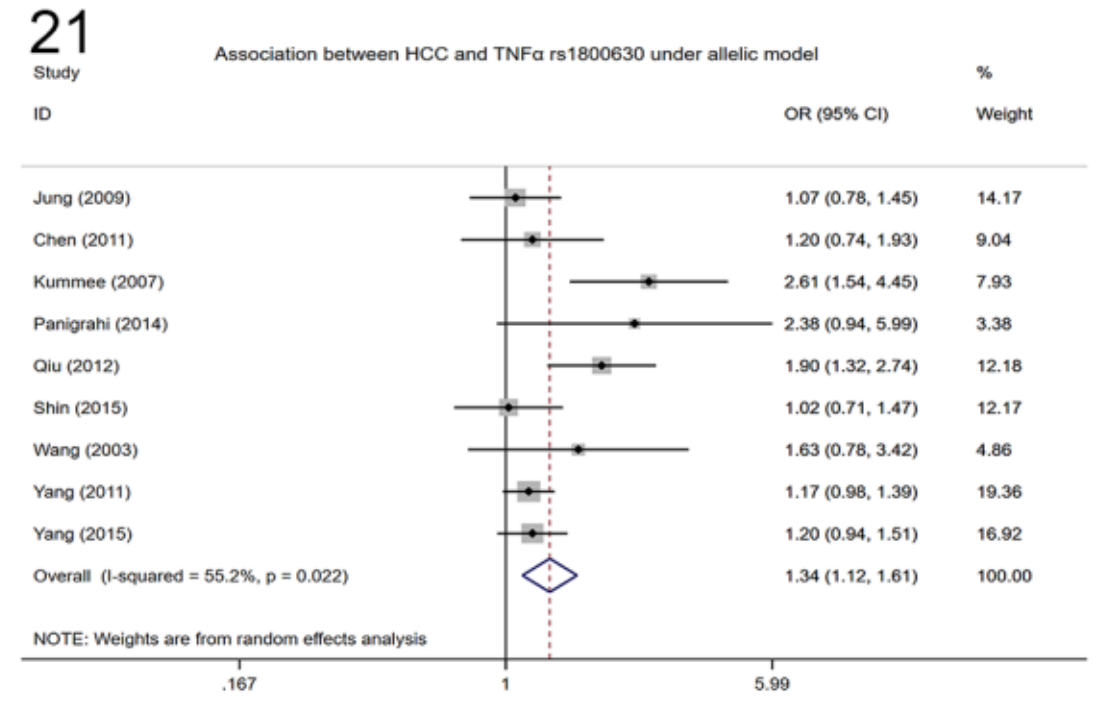

22

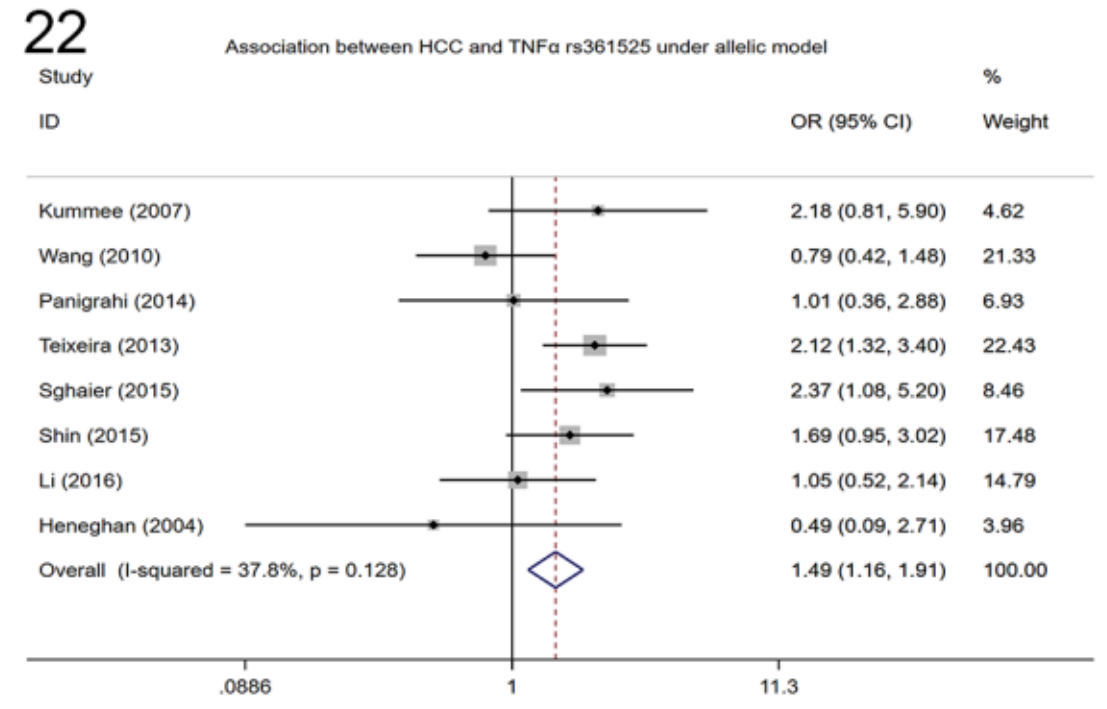

23

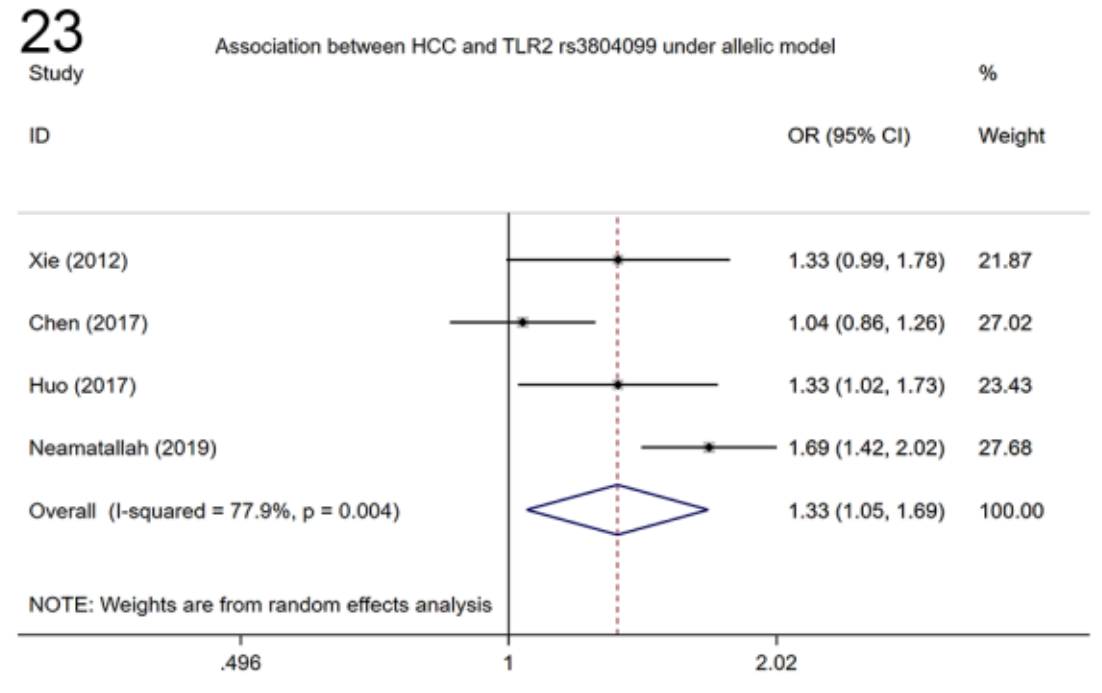

24

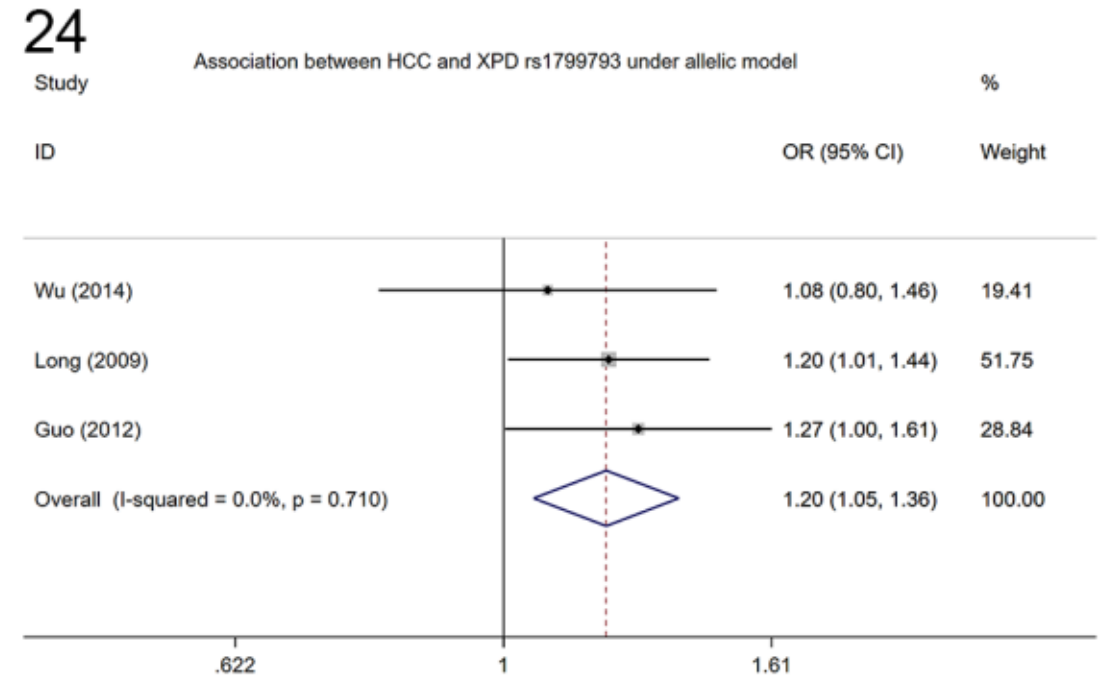

25

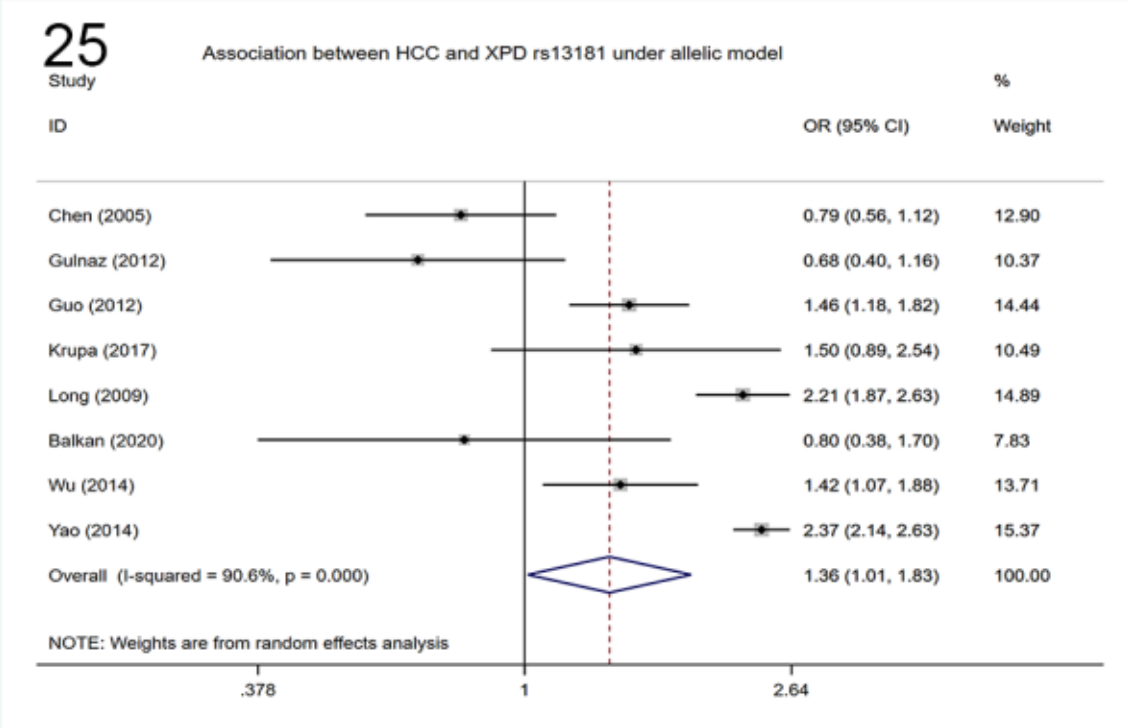

26

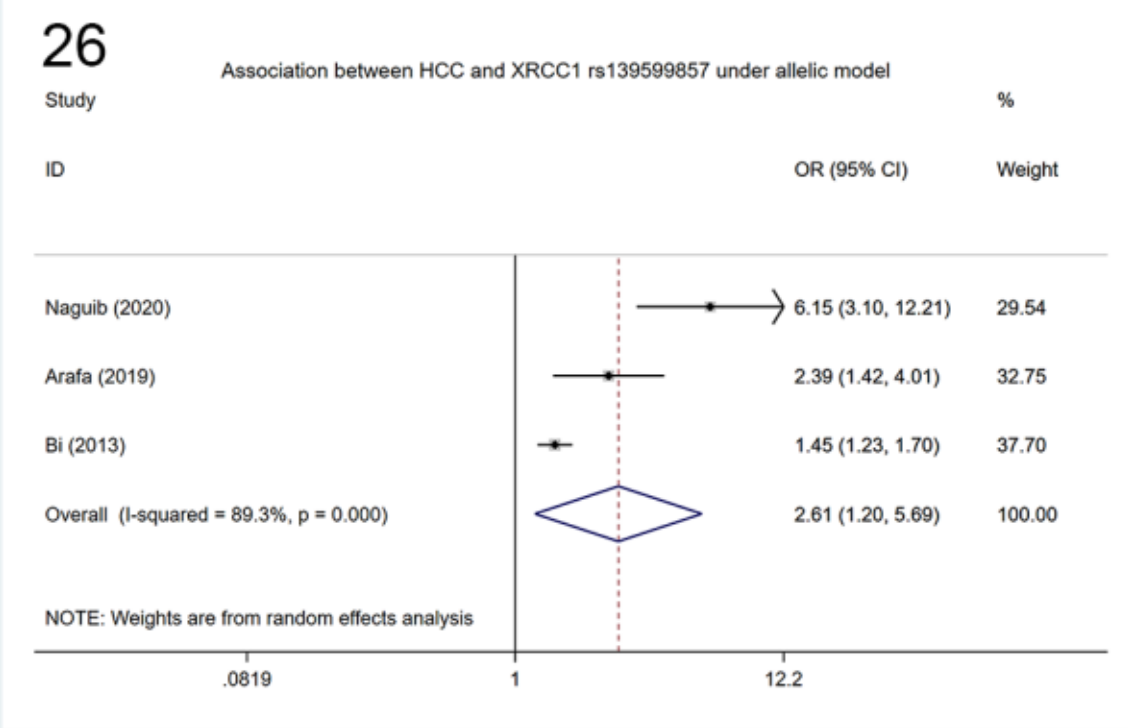

27

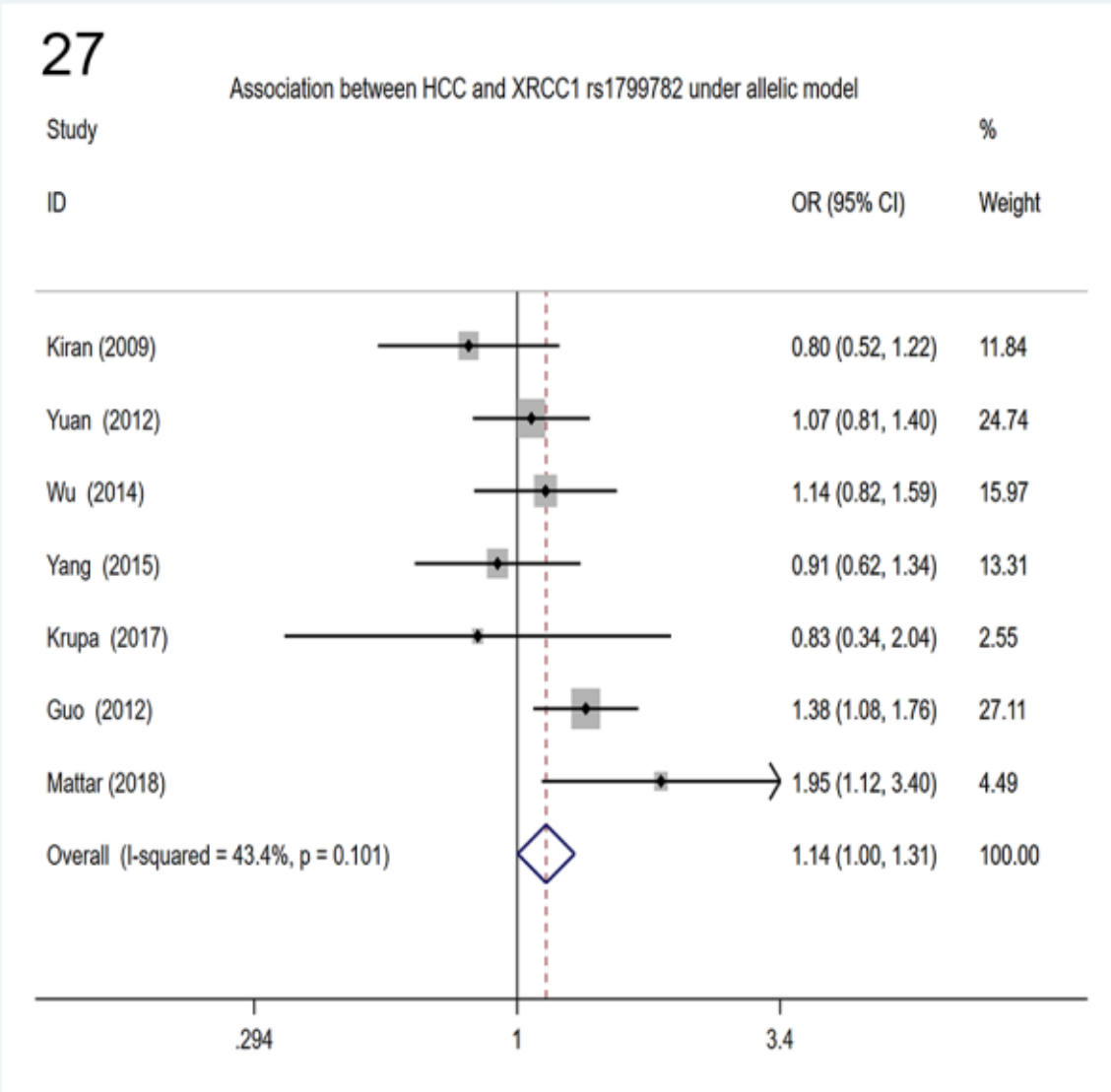

28.1

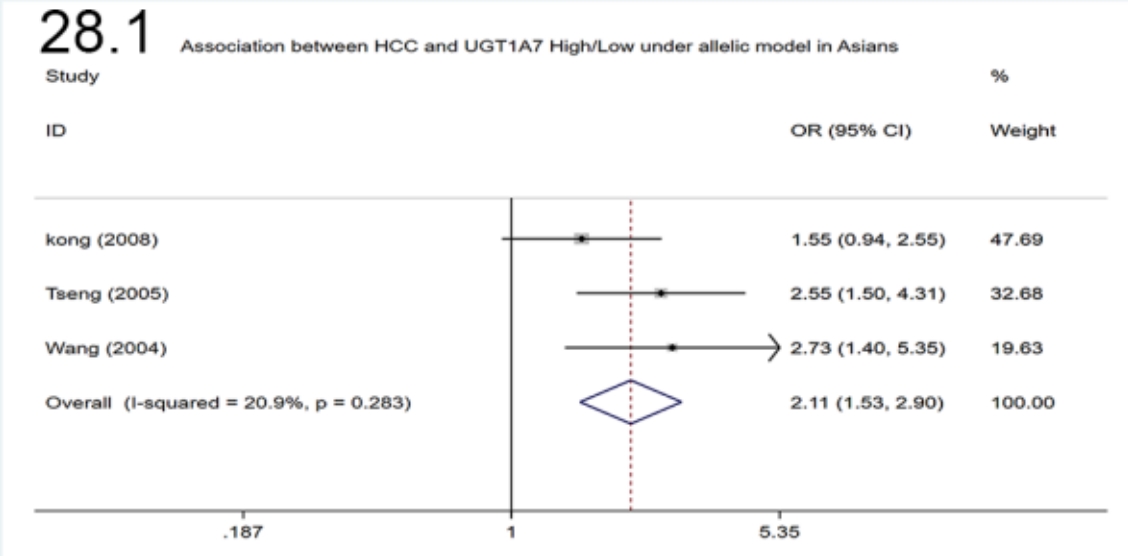

28.2

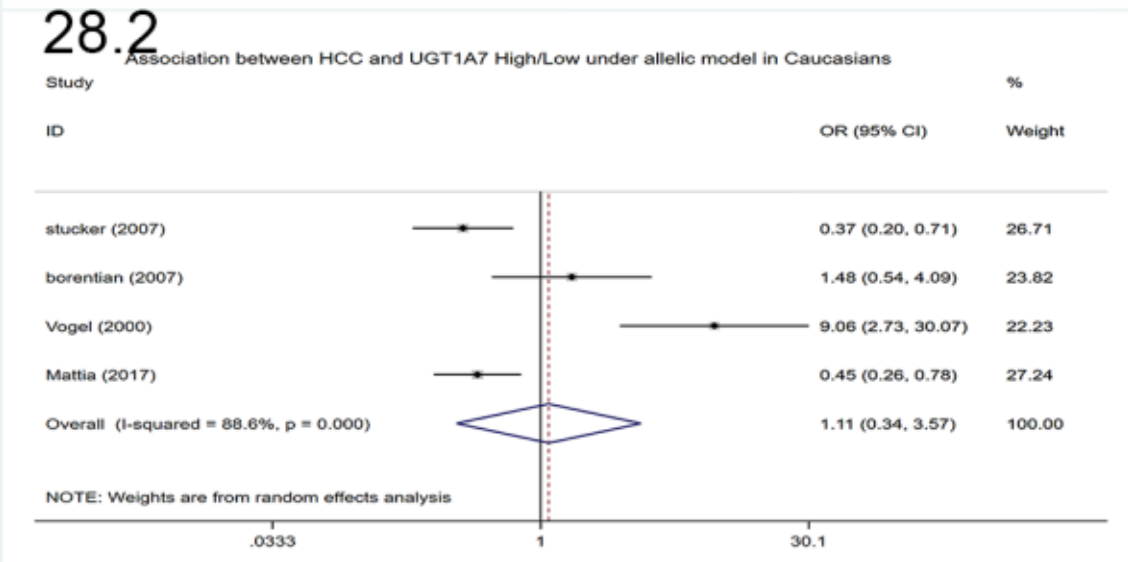

29

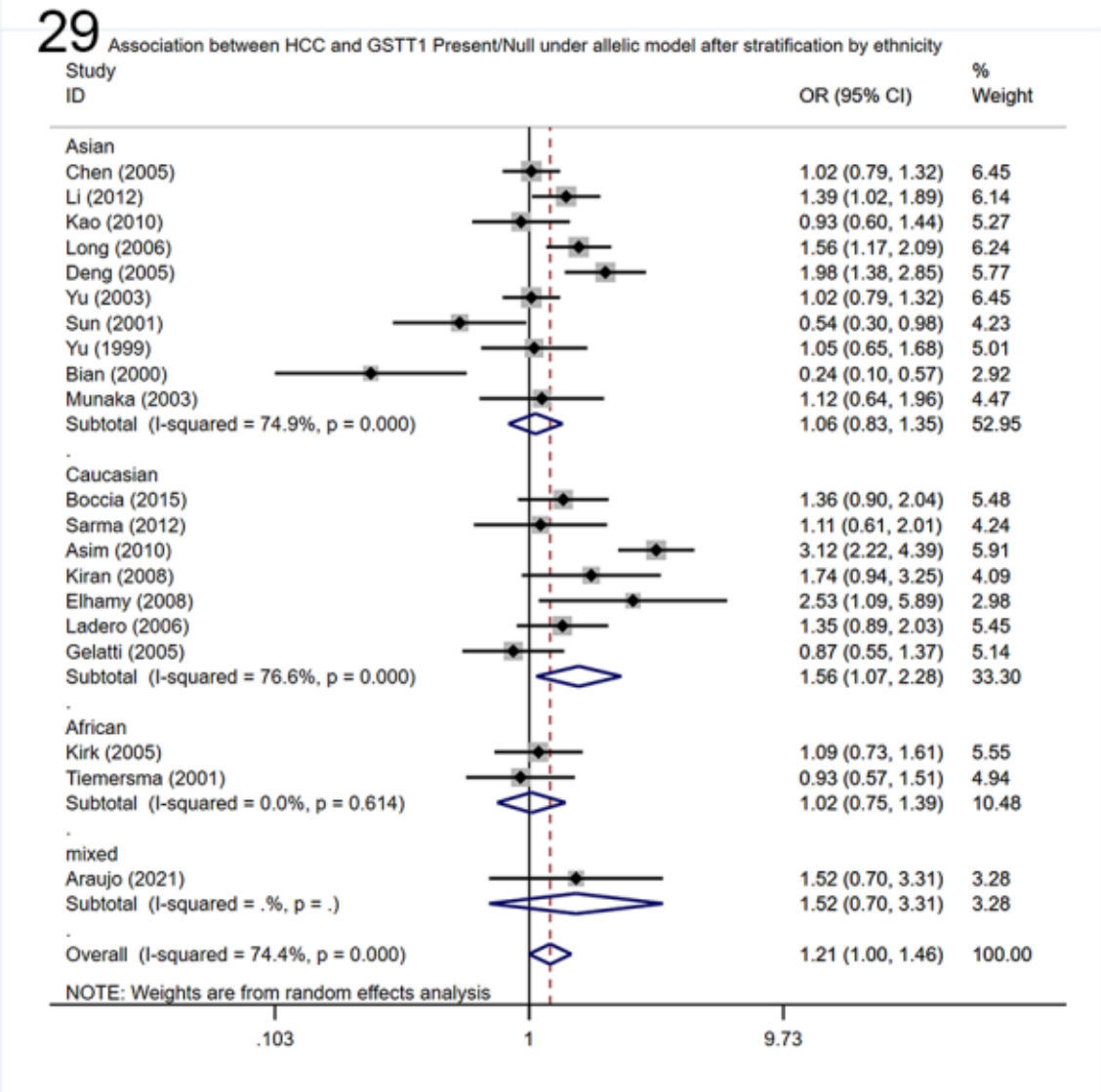

30

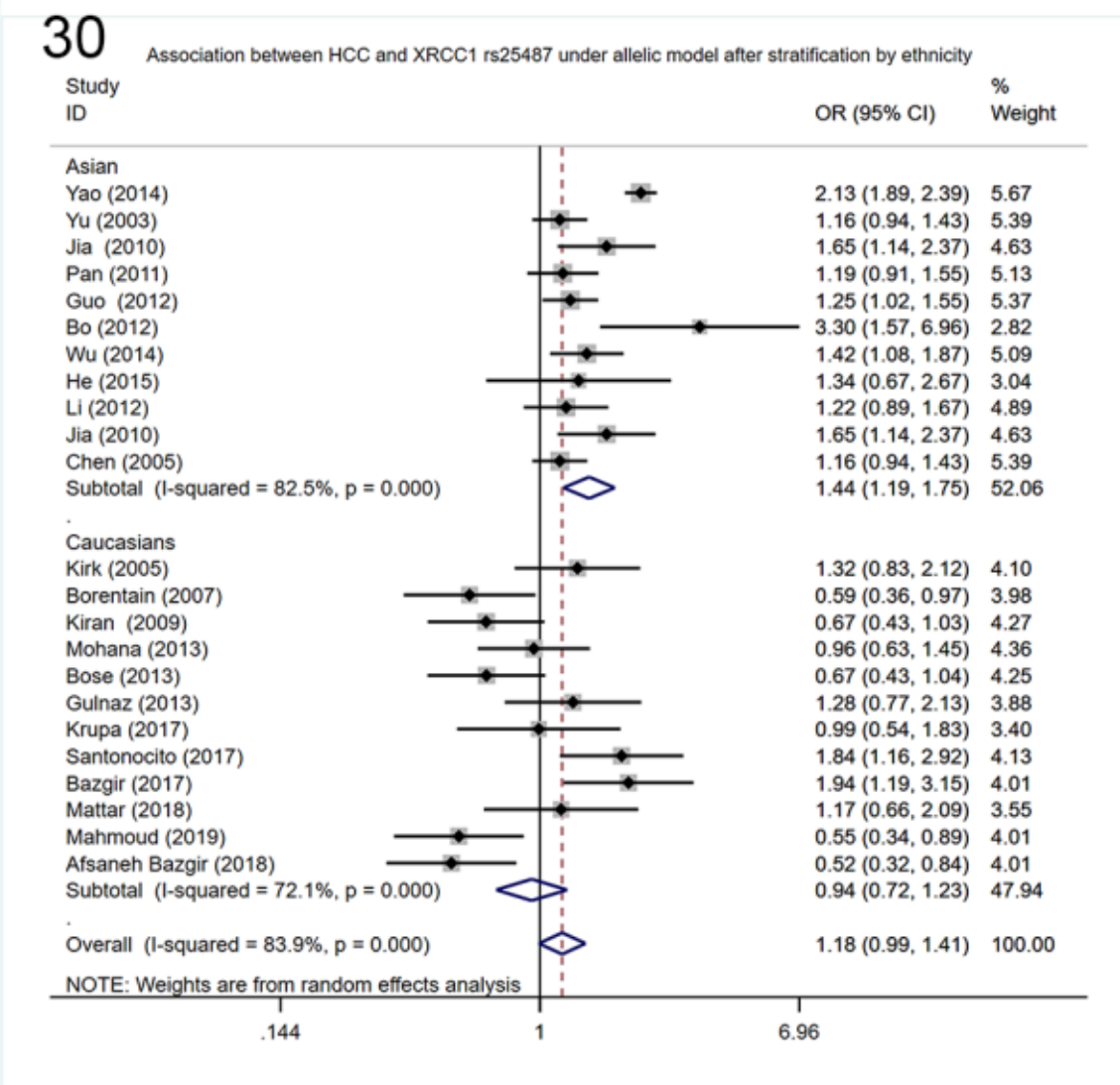

12. Figure S1 Forest plot of additional genetic variants significantly associated with risk of hepatocellular carcinoma as confirmed in meta-analyses in the recessive or dominant model.

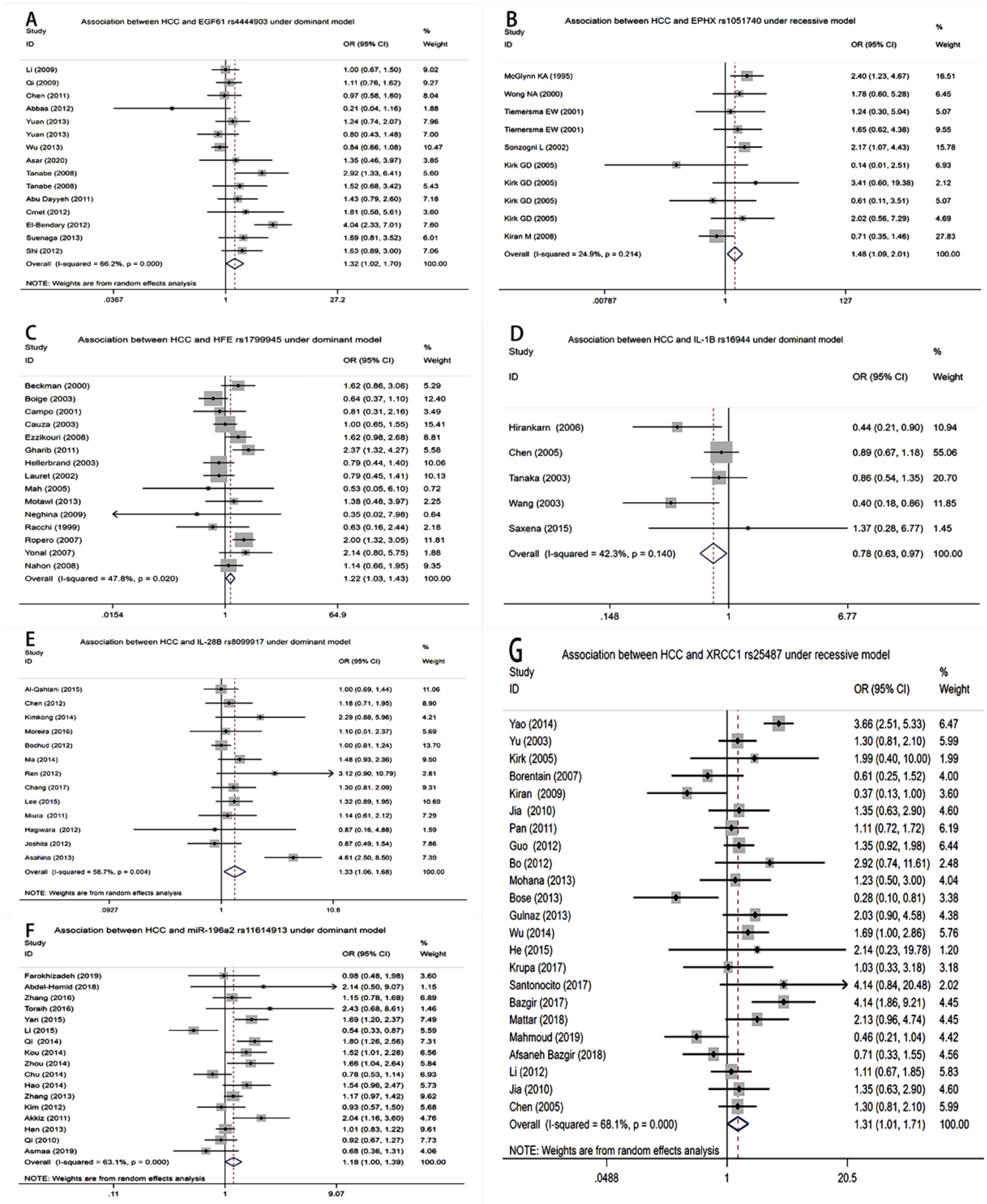

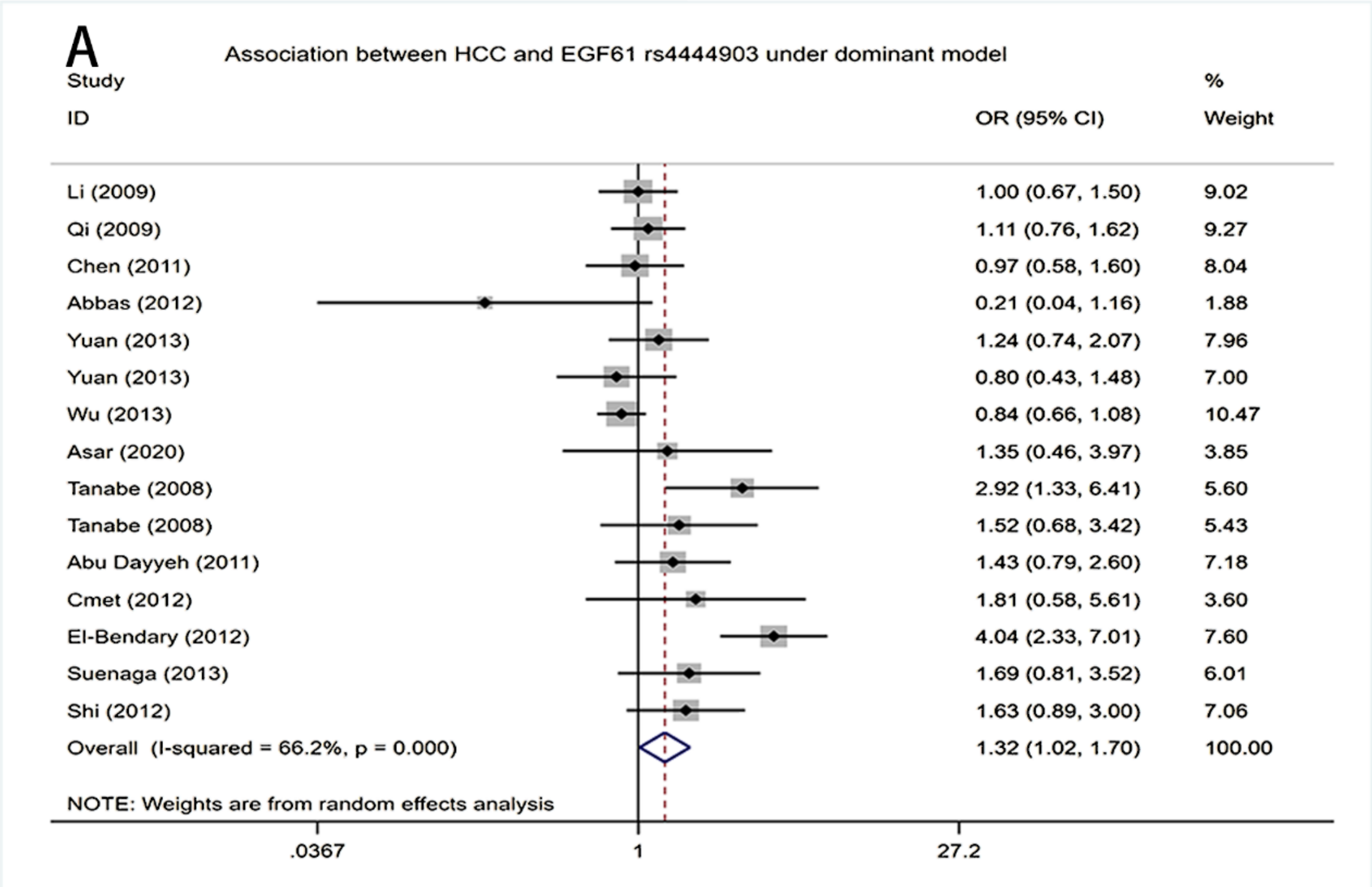
A
Association between HCC and EGF61 rs4444903 under dominant model
Study
ID
OR (95% CI)
%
Weight
Li (2009) 1.00 (0.67, 1.50) 9.02
Qi (2009) 1.11 (0.76, 1.62) 9.27
Chen (2011) 0.97 (0.58, 1.60) 8.04
Abbas (2012) 0.21 (0.04, 1.16) 1.88
Yuan (2013) 1.24 (0.74, 2.07) 7.96
Yuan (2013) 0.80 (0.43, 1.48) 7.00
Wu (2013) 0.84 (0.66, 1.08) 10.47
Asar (2020) 1.35 (0.46, 3.97) 3.85
Tanabe (2008) 2.92 (1.33, 6.41) 5.60
Tanabe (2008) 1.52 (0.68, 3.42) 5.43
Abu Dayyeh (2011) 1.43 (0.79, 2.60) 7.18
Cmet (2012) 1.81 (0.58, 5.61) 3.60
El-Bendary (2012) 4.04 (2.33, 7.01) 7.60
Suenaga (2013) 1.69 (0.81, 3.52) 6.01
Shi (2012) 1.63 (0.89, 3.00) 7.06
Overall (I-squared = 66.2%, p = 0.000) 1.32 (1.02, 1.70) 100.00
NOTE: Weights are from random effects analysis
.0367
1
27.2

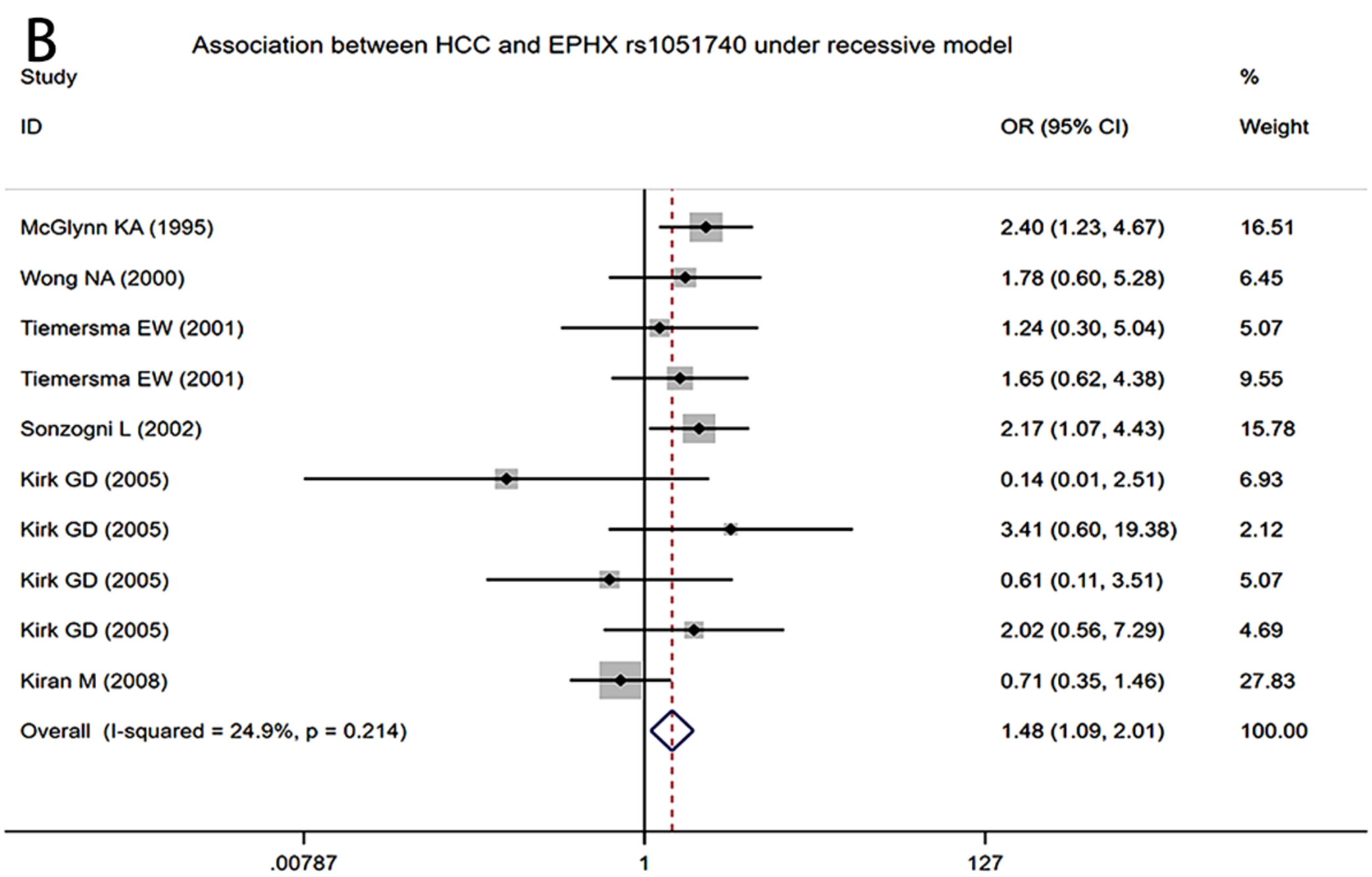
B
Association between HCC and EPHX rs1051740 under recessive model
Study
ID
OR (95% CI)
%
Weight
McGlynn KA (1995) 2.40 (1.23, 4.67) 16.51
Wong NA (2000) 1.78 (0.60, 5.28) 6.45
Tiemersma EW (2001) 1.24 (0.30, 5.04) 5.07
Tiemersma EW (2001) 1.65 (0.62, 4.38) 9.55
Sonzogni L (2002) 2.17 (1.07, 4.43) 15.78
Kirk GD (2005) 0.14 (0.01, 2.51) 6.93
Kirk GD (2005) 3.41 (0.60, 19.38) 2.12
Kirk GD (2005) 0.61 (0.11, 3.51) 5.07
Kirk GD (2005) 2.02 (0.56, 7.29) 4.69
Kiran M (2008) 0.71 (0.35, 1.46) 27.83
Overall (I-squared = 24.9%, p = 0.214) 1.48 (1.09, 2.01) 100.00
.00787
1
127

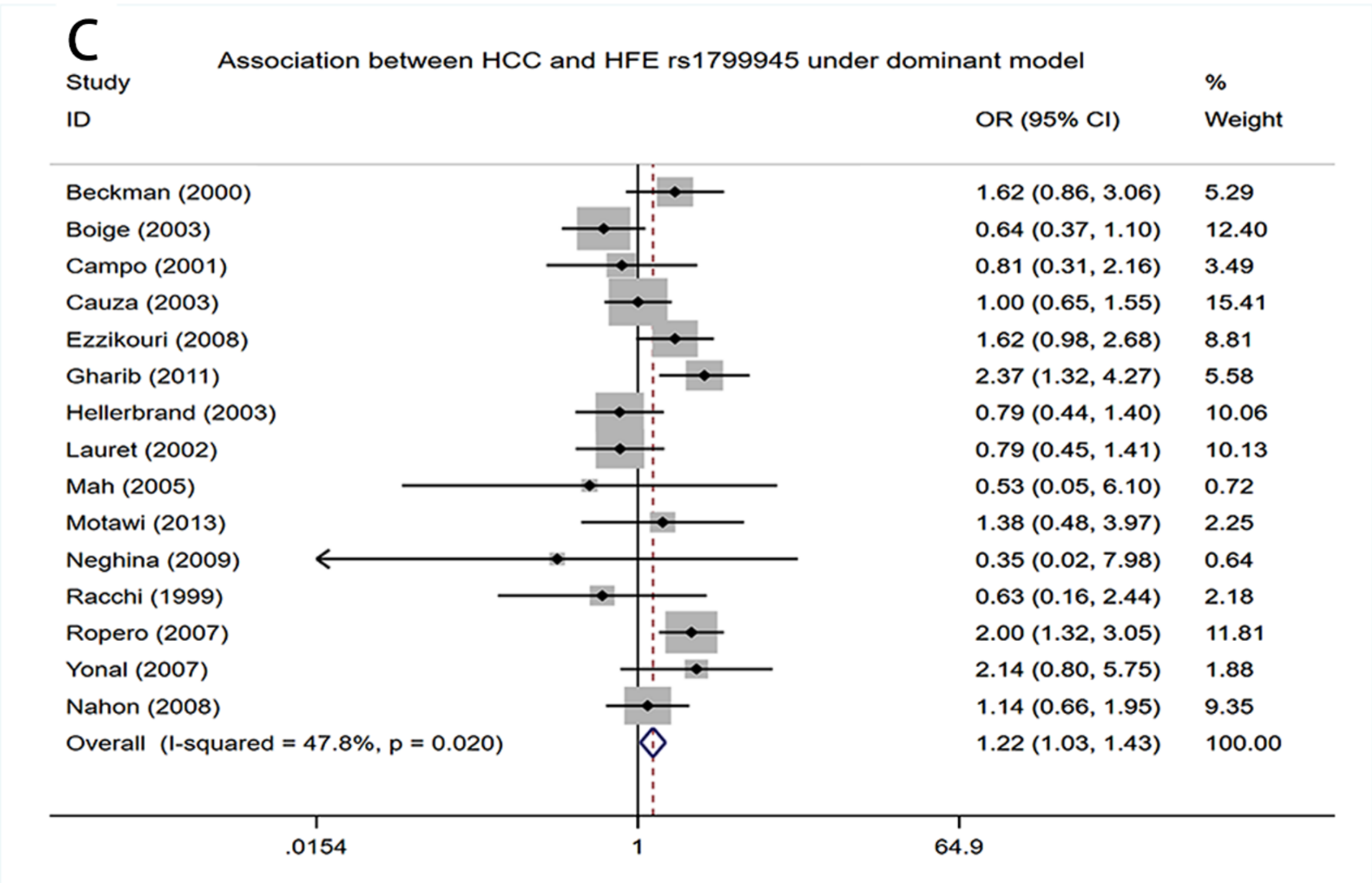
C
Association between HCC and HFE rs1799945 under dominant model
Study
ID
OR (95% CI)
%
Weight
Beckman (2000) 1.62 (0.86, 3.06) 5.29
Boige (2003) 0.64 (0.37, 1.10) 12.40
Campo (2001) 0.81 (0.31, 2.16) 3.49
Cauza (2003) 1.00 (0.65, 1.55) 15.41
Ezzikouri (2008) 1.62 (0.98, 2.68) 8.81
Gharib (2011) 2.37 (1.32, 4.27) 5.58
Hellerbrand (2003) 0.79 (0.44, 1.40) 10.06
Lauret (2002) 0.79 (0.45, 1.41) 10.13
Mah (2005) 0.53 (0.05, 6.10) 0.72
Motawi (2013) 1.38 (0.48, 3.97) 2.25
Neghina (2009) 0.35 (0.02, 7.98) 0.64
Racchi (1999) 0.63 (0.16, 2.44) 2.18
Ropero (2007) 2.00 (1.32, 3.05) 11.81
Yonal (2007) 2.14 (0.80, 5.75) 1.88
Nahon (2008) 1.14 (0.66, 1.95) 9.35
Overall (I-squared = 47.8%, p = 0.020) 1.22 (1.03, 1.43) 100.00
.0154
1
64.9

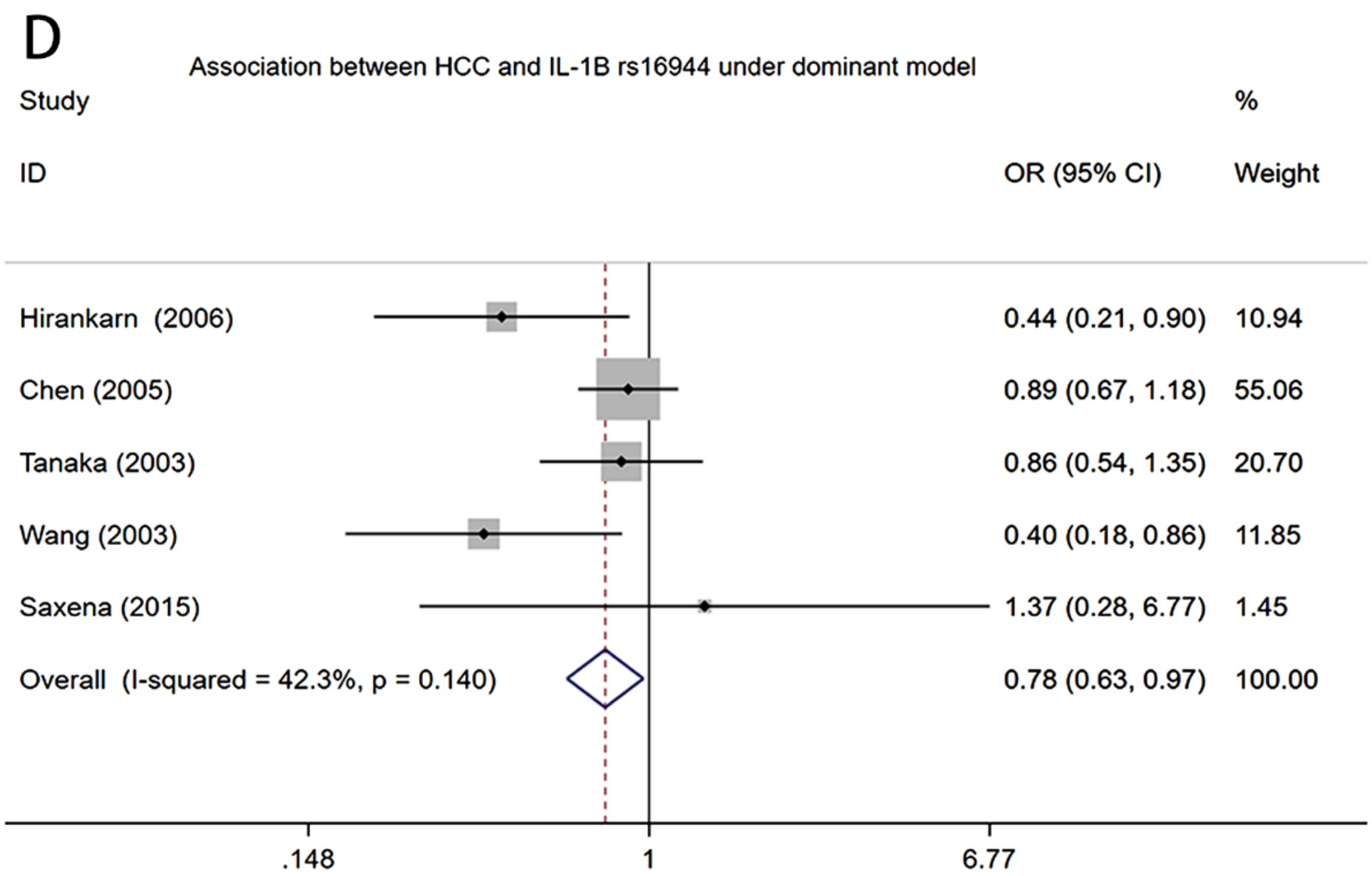
D
Association between HCC and IL-1B rs16944 under dominant model
Study
ID
OR (95% CI)
%
Weight
Hirankarn (2006) 0.44 (0.21, 0.90) 10.94
Chen (2005) 0.89 (0.67, 1.18) 55.06
Tanaka (2003) 0.86 (0.54, 1.35) 20.70
Wang (2003) 0.40 (0.18, 0.86) 11.85
Saxena (2015) 1.37 (0.28, 6.77) 1.45
Overall (I-squared = 42.3%, p = 0.140) 0.78 (0.63, 0.97) 100.00
.148
1
6.77

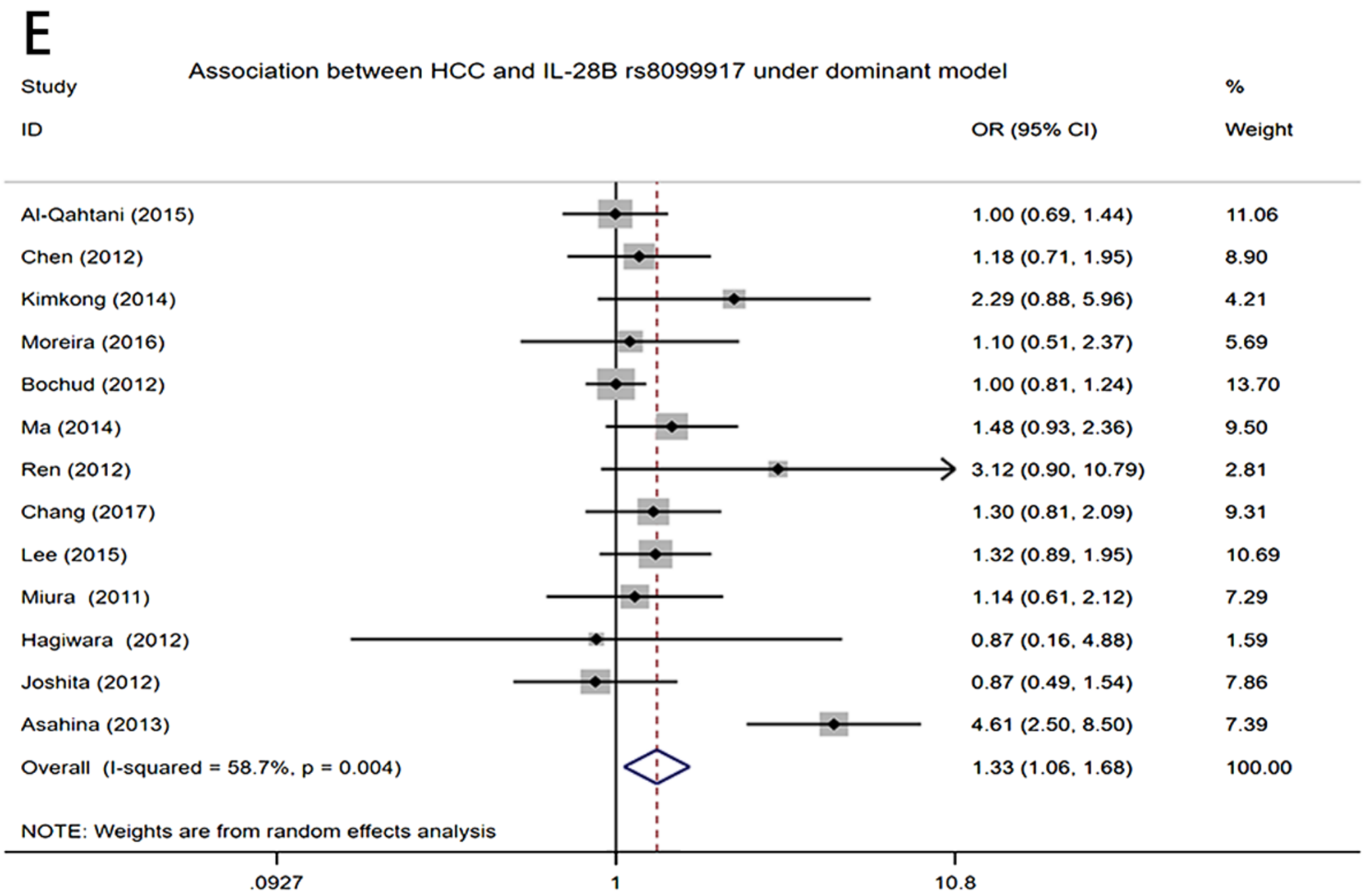
E
Association between HCC and IL-28B rs8099917 under dominant model
Study
ID
OR (95% CI)
%
Weight
Al-Qahtani (2015) 1.00 (0.69, 1.44) 11.06
Chen (2012) 1.18 (0.71, 1.95) 8.90
Kimkong (2014) 2.29 (0.88, 5.96) 4.21
Moreira (2016) 1.10 (0.51, 2.37) 5.69
Bochud (2012) 1.00 (0.81, 1.24) 13.70
Ma (2014) 1.48 (0.93, 2.36) 9.50
Ren (2012) 3.12 (0.90, 10.79) 2.81
Chang (2017) 1.30 (0.81, 2.09) 9.31
Lee (2015) 1.32 (0.89, 1.95) 10.69
Miura (2011) 1.14 (0.61, 2.12) 7.29
Hagiwara (2012) 0.87 (0.16, 4.88) 1.59
Joshita (2012) 0.87 (0.49, 1.54) 7.86
Asahina (2013) 4.61 (2.50, 8.50) 7.39
Overall (I-squared = 58.7%, p = 0.004) 1.33 (1.06, 1.68) 100.00
NOTE: Weights are from random effects analysis
.0927
1
10.8

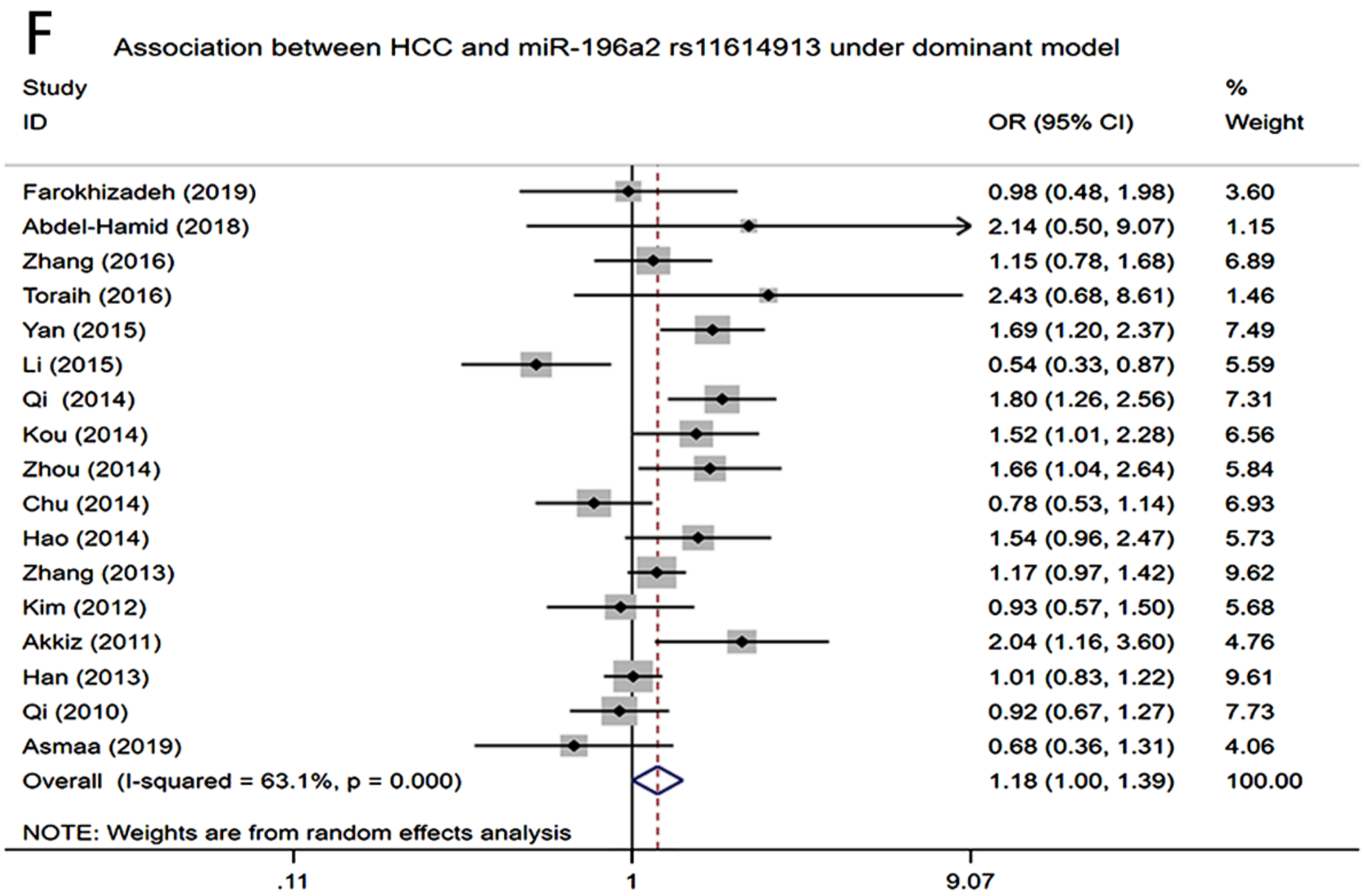
F
Association between HCC and miR-196a2 rs11614913 under dominant model
Study
ID
OR (95% CI)
%
Weight
Farokhizadeh (2019) 0.98 (0.48, 1.98) 3.60
Abdel-Hamid (2018) 2.14 (0.50, 9.07) 1.15
Zhang (2016) 1.15 (0.78, 1.68) 6.89
Toraih (2016) 2.43 (0.68, 8.61) 1.46
Yan (2015) 1.69 (1.20, 2.37) 7.49
Li (2015) 0.54 (0.33, 0.87) 5.59
Qi (2014) 1.80 (1.26, 2.56) 7.31
Kou (2014) 1.52 (1.01, 2.28) 6.56
Zhou (2014) 1.66 (1.04, 2.64) 5.84
Chu (2014) 0.78 (0.53, 1.14) 6.93
Hao (2014) 1.54 (0.96, 2.47) 5.73
Zhang (2013) 1.17 (0.97, 1.42) 9.62
Kim (2012) 0.93 (0.57, 1.50) 5.68
Akkiz (2011) 2.04 (1.16, 3.60) 4.76
Han (2013) 1.01 (0.83, 1.22) 9.61
Qi (2010) 0.92 (0.67, 1.27) 7.73
Asmaa (2019) 0.68 (0.36, 1.31) 4.06
Overall (I-squared = 63.1%, p = 0.000) 1.18 (1.00, 1.39) 100.00
NOTE: Weights are from random effects analysis
.11
1
9.07

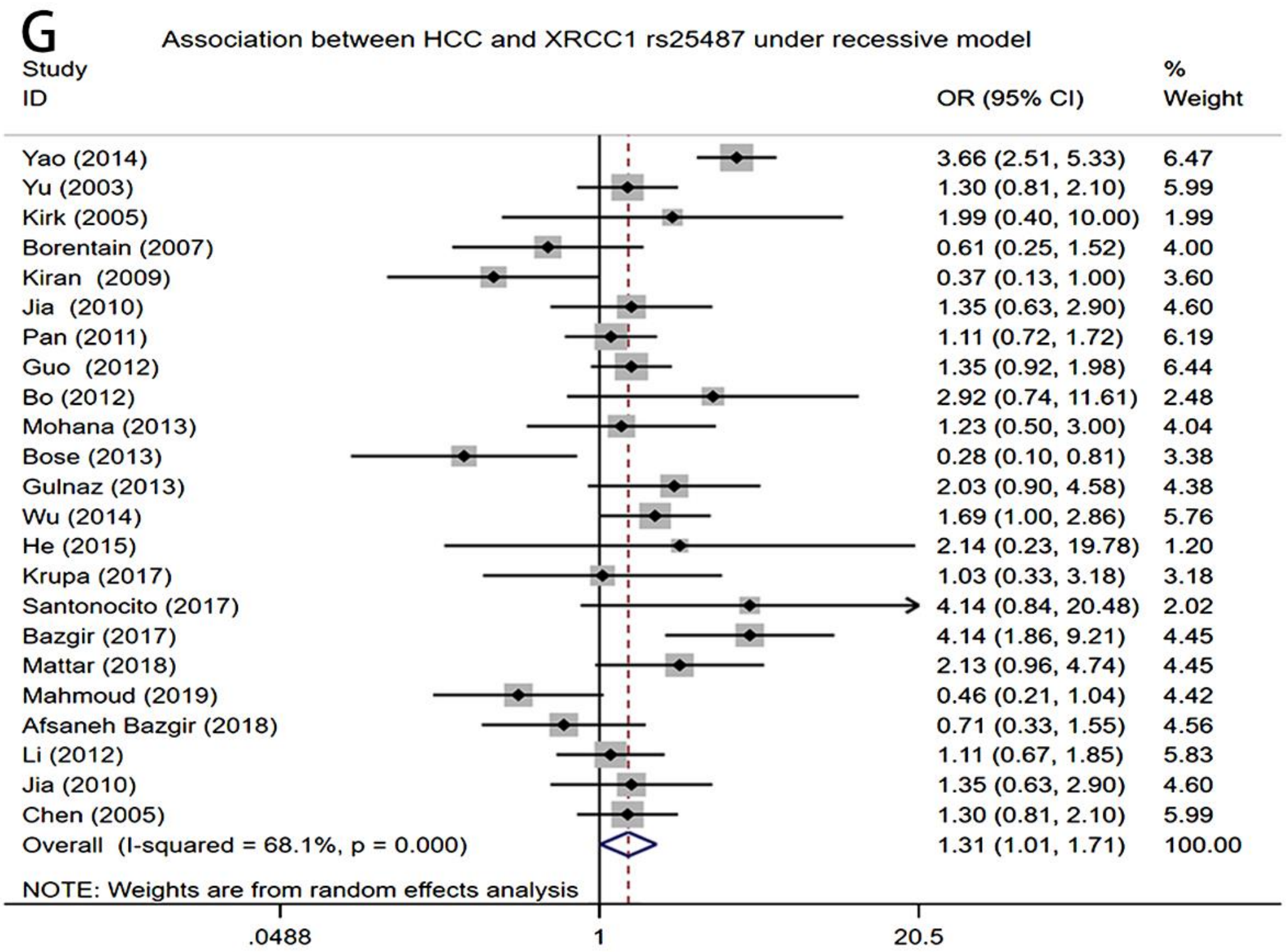
G
Association between HCC and XRCC1 rs25487 under recessive model
Study ID
OR (95% CI)
% Weight
Yao (2014) 3.66 (2.51, 5.33) 6.47
Yu (2003) 1.30 (0.81, 2.10) 5.99
Kirk (2005) 1.99 (0.40, 10.00) 1.99
Borentain (2007) 0.61 (0.25, 1.52) 4.00
Kiran (2009) 0.37 (0.13, 1.00) 3.60
Jia (2010) 1.35 (0.63, 2.90) 4.60
Pan (2011) 1.11 (0.72, 1.72) 6.19
Guo (2012) 1.35 (0.92, 1.98) 6.44
Bo (2012) 2.92 (0.74, 11.61) 2.48
Mohana (2013) 1.23 (0.50, 3.00) 4.04
Bose (2013) 0.28 (0.10, 0.81) 3.38
Gulnaz (2013) 2.03 (0.90, 4.58) 4.38
Wu (2014) 1.69 (1.00, 2.86) 5.76
He (2015) 2.14 (0.23, 19.78) 1.20
Krupa (2017) 1.03 (0.33, 3.18) 3.18
Santonocito (2017) 4.14 (0.84, 20.48) 2.02
Bazgir (2017) 4.14 (1.86, 9.21) 4.45
Mattar (2018) 2.13 (0.96, 4.74) 4.45
Mahmoud (2019) 0.46 (0.21, 1.04) 4.42
Afsaneh Bazgir (2018) 0.71 (0.33, 1.55) 4.56
Li (2012) 1.11 (0.67, 1.85) 5.83
Jia (2010) 1.35 (0.63, 2.90) 4.60
Chen (2005) 1.30 (0.81, 2.10) 5.99
Overall (I-squared = 68.1%, p = 0.000) 1.31 (1.01, 1.71) 100.00
NOTE: Weights are from random effects analysis
.0488
1
20.5
